# The *GRAS* gene family and its roles in seed development in litchi (*Litchi chinensis* Sonn)

**DOI:** 10.1186/s12870-021-03193-1

**Published:** 2021-09-17

**Authors:** Jingwen Chen, Qian Yan, Jiawei Li, Lei Feng, Yi Zhang, Jing Xu, Rui Xia, Zaohai Zeng, Yuanlong Liu

**Affiliations:** 1 State Key Laboratory for Conservation and Utilization of Subtropical Agro-Bioresources, South China Agricultural University, 483 Wushan Road, Tianhe, Guangzhou, 510642 Guangdong Province China; 2 Key Laboratory of Biology and Germplasm Enhancement of Horticultural Crops in South China, Ministry of Agriculture, South China Agricultural University, Guangzhou, China; 3 Guangdong Litchi Engineering Research Center, College of Horticulture, South China Agricultural University, Guangzhou, China; 4 Key Laboratory of South Subtropical Fruit Biology and Genetic Resource Utilization, Ministry of Agriculture / Guangdong ProvinceKey Laboratary of Tropical and Subtropical Fruit Tree Research / Institute of Fruit Tree Research, Guangdong Academy of Agricultural Sciences, Guangzhou, China

**Keywords:** Litchi, *GRAS* gene family, miR171, Seed development

## Abstract

**Background:**

The *GRAS* gene family plays crucial roles in multiple biological processes of plant growth, including seed development, which is related to seedless traits of litchi (*Litchi chinensis* Sonn.). However, it hasn’t been fully identified and analyzed in litchi, an economic fruit tree cultivated in subtropical regions.

**Results:**

In this study, 48 LcGRAS proteins were identified and termed according to their chromosomal location. LcGRAS proteins can be categorized into 14 subfamilies through phylogenetic analysis. Gene structure and conserved domain analysis revealed that different subfamilies harbored various motif patterns, suggesting their functional diversity. Synteny analysis revealed that the expansion of the *GRAS* family in litchi may be driven by their tandem and segmental duplication. After comprehensively analysing degradome data, we found that four *LcGRAS* genes belong to HAM subfamily were regulated via miR171-mediated degradation. The various expression patterns of *LcGRAS* genes in different tissues uncovered they were involved in different biological processes. Moreover, the different temporal expression profiles of *LcGRAS* genes between abortive and bold seed indicated some of them were involved in maintaining the normal development of the seed.

**Conclusion:**

Our study provides comprehensive analyses on *GRAS* family members in litchi, insight into a better understanding of the roles of *GRAS* in litchi development, and lays the foundation for further investigations on litchi seed development.

**Supplementary Information:**

The online version contains supplementary material available at 10.1186/s12870-021-03193-1.

## Background

*GRAS* is a major plant-specific transcription factor gene family among numerous transcription factors that are proved to function in plant growth and development, whose name is termed from the first three functionally characterized members, gibberellic-acid insensitive (GAI) [1], repressor of GAI (RGA) [2], and scarecrow (SCR) [3]. Typically, the GRAS proteins usually encompass 400–770 amino acids (aa) [4] and contain a variable N-terminal region and a highly conserved C-terminal region. The conserved region was composed of five motifs: LHRI, LHRII, VHIID, PFYRE, and SAW [4–6]. GRAS proteins were divided into eight basic subfamilies in *Arabidopsis thaliana* and rice (*Oryza sativa* L.) according to their common feature [6]. Whereas in other plants such as *Prunus mume* [7], *Medicago truncatula* [8], Chinese Cabbage (*Brassica rapa* ssp. *pekinensis*) [9], pepper (*Capsicum annuum* L.) [10], tomato (*Solanum lycopersicum*) [11], sacred lotus (*Nelumbo nucifera*) [12], tea plant (*Camellia sinensis*) [13], *Populus* [14], pine (*Pinus radiata*) [15], castor beans (*Ricinus communis*) [16], Tartary buckwheat (*Fagopyrum tataricum*) [17], and cotton (*Gossypium hirsutum* L.) [18], the number of subfamilies varied from eight to 14, suggesting that specific subfamilies might be present in these species.

GRAS proteins are involved in many physiological processes such as signal transduction, root radial patterning and development, stress responses and meristem development [19]. However, each subfamily may have different functions. For instance, DELLA proteins act as inhibitory factors in the gibberellic acid (GA) signal transduction pathway and modulate the jasmonic acid (JA) signal transduction [20]. PAT1 is participating in phytochrome signaling of *Arabidopsis* [21]. Besides, SCR and SHR proteins are involved in root and shoot radial patterning in *Arabidopsis* [22]. SCL3 acts as an integrator downstream of the GA/DELLA and SCR/SHR pathways, mediating the GA-promoted cell elongation during root development [23]. Moreover, OsMOC1, AtLAS, and SlLS play important roles in axillary meristem initiation, plant tillering, and control of grain yield [24–26]. Additionally, SCL13 with stress-related functions have been discovered in cabbage [27], petunia HAM mediates signals from differentiating cells for functioning in shoot meristem maintenance [28].

It has been mentioned in previous studies that some *GRAS* genes from HAM subfamily were targeted by miR171, which play diverse roles in plant development, such as flowering and phase transition [29, 30]. A miR171-*SCL6* model contributes to embryogenic callus induction and torpedo-shaped embryo formation during somatic embryogenesis in two lily species [31]. In *Arabidopsis*, *SCL6*/*SCL6-IV*, *SCL22*/*SCL6-III*, and *SCL27*/*SCL6-II* are also known as targets of miR171, and play pivotal roles in the proliferation of meristematic cells [32, 33]. Overexpression of a tomato miR171 target gene, *SlGRAS24*, impacts multiple agronomical traits such as plant height, flowering time, root length, fruit set and development [34].

Litchi is a subtropical fruit tree of family Sapindaceae with great economic and nutritional value. Based on fruit anatomy, the fruit of litchi is a drupe with an edible aril enclosing a single seed surrounded by a pericarp [35]. Small seeds or seedlessness is an economically desirable trait of litchi, which lead to great market value. In flowering plants, seed development is preceded by a double fertilization event, which form the precursor cells of embryo and endosperm. The endosperm is essential for the development of an embryo and the rapid disintegration of endosperm lead to the abortion of seed. Coordination in the growth of endosperm and embryo is crucial during early seed development, results in the discrepancy in seed size in a fruit [36]. A bunch of *GRAS* genes were supposed to take part in determining endosperm and embryo development in several species, affecting seed development. In lily, a *GRAS* gene belonging to the LlSCL subfamily, plays a role in the microsporogenesis process of the anther [37]. *GS6*, a unique member of the GRAS gene family, was responsible for the reduction of grain size and weight during the domestication of rice [38]. In *P. mume*, 25% of *GRAS* genes showed higher expression in seeds [7]. In apple, the higher expression level of *MdGRAS126*, *MdGRAS18*, and *MdGRAS79* in seeds in accordance with the finding in *P. mume* [7, 39], indicating the important roles of *GRAS* genes in seed development. However, the biological function of GRAS proteins in seed development of litchi remains scarce. Hence, a comprehensive analysis of *GRAS* genes in litchi would be informative in laying foundation for the characterization of their potential function, especially in seed development.

In our study, two different litchi cultivars (‘Huaizhi’ and ‘NMC’) and a wildtype litchi (‘WL10’) were used to carry out a genome-wide analysis of *GRAS* genes in litchi. As a result, 48 *LcGRAS* genes were identified. Gene structure, phylogeny, chromosomal distributions, duplication events, dual synteny analysis, and miRNA-mediated regulation were characterized. Expression pattern of *LcGRAS* genes was detected in various tissues and four different stages during seed development, including 15, 25, 35, and 45 days after anthesis (DAA), representing near globular-shaped, heart-shaped, torpedo-shaped and cotyledon-shaped embryo stage. Among them, *LcGRAS1*, *LcGRAS15*, *LcGRAS24*, *LcGRAS28*, *LcGRAS29*, *LcGRAS40*, and *LcGRAS48* were found to exert its potential function in seed development of litchi via auxin and GA pathway.

## Results

### Identification and phylogenetic analysis of *LcGRAS* genes

Based on homology analysis, 48 LcGRAS proteins were identified from the litchi genome (Additional file 1: Table S1, Additional file 2), which were renamed from LcGRAS1 to LcGRAS48 according to the chromosomal location. The length of LcGRAS proteins was between 422 aa (LcGRAS47) and 803 aa (LcGRAS34). The predicted molecular weight (MW) of the proteins ranged from 47.24 kDa (LcGRAS18) to 89.89 kDa (LcGRAS34), and the predicted isoelectric point (pI) ranged from 4.62 (LcGRAS30) to 8.67 (LcGRAS7) (Additional file 1: Table S2). The numerical range of the above characteristics is similar to that of other species [7, 10], indicating that our identification of LcGRASs is relatively accurate, and the basic characteristics of GRASs are relatively conserved in different species.

**Fig. 1:**
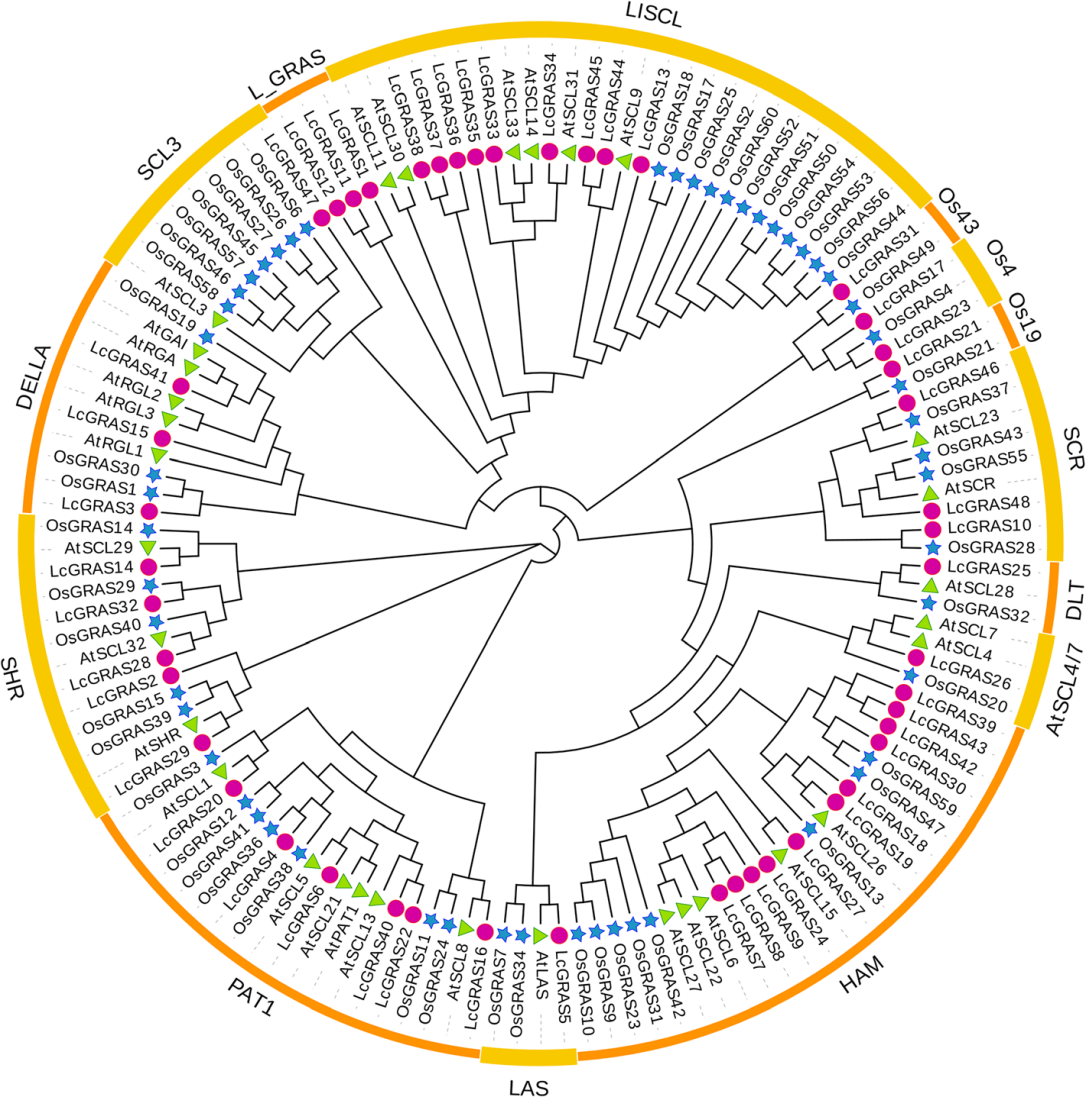
Phylogenetic tree of the *GRAS* gene family in litchi, rice, and *Arabidopsis*. The phylogenetic tree was constructed using Maximam-Likelihood (ML) method by MEGA7.0. Subfamilies were marked by bold yellow lines or orange lines in the external circle. The pink circles, green triangles and blue stars represent *GRAS* genes from litchi, *Arabidopsis* and rice, respectively

To explore the phylogenetic relationship of LcGRAS protein, we constructed a phylogenetic tree based on the amino acid sequences of 48 LcGRAS, 32 AtGRAS, and 53 OsGRAS proteins. According to previous studies [6, 18], 48 LcGRAS members were divided into 14 subfamilies: SCR, SHR, DELLA, PAT1, HAM, LISCL, LAS, SCL3, SCL4/7, DLT, Os4, Os19, Os43 and L_GRAS (Fig. 1). The HAM subfamily possessed the most LcGRAS members (11), followed by LISCL (9), PAT1 (6), SHR (5), DELLA (3), SCR (3), SCL3 (1), LAS (1), AtSCL4/7 (1), and DLT (1). In addition, 2, 1, 1 of LcGRAS proteins were respectively grouped into the Os4, Os19, and Os43 subfamilies, all of which were previously reported as rice-specific [14]. These LcGRAS members may exist before the divergence of dicotyledons and monocotyledons and lost in *Arabidopsis*. Furthermore, the L_GRAS subfamily contained three members, all of which were from litchi, implying that this litchi-specific subfamily may have unique functions in litchi or close species.

### Gene structure and conserved domain analysis

**Fig. 2:**
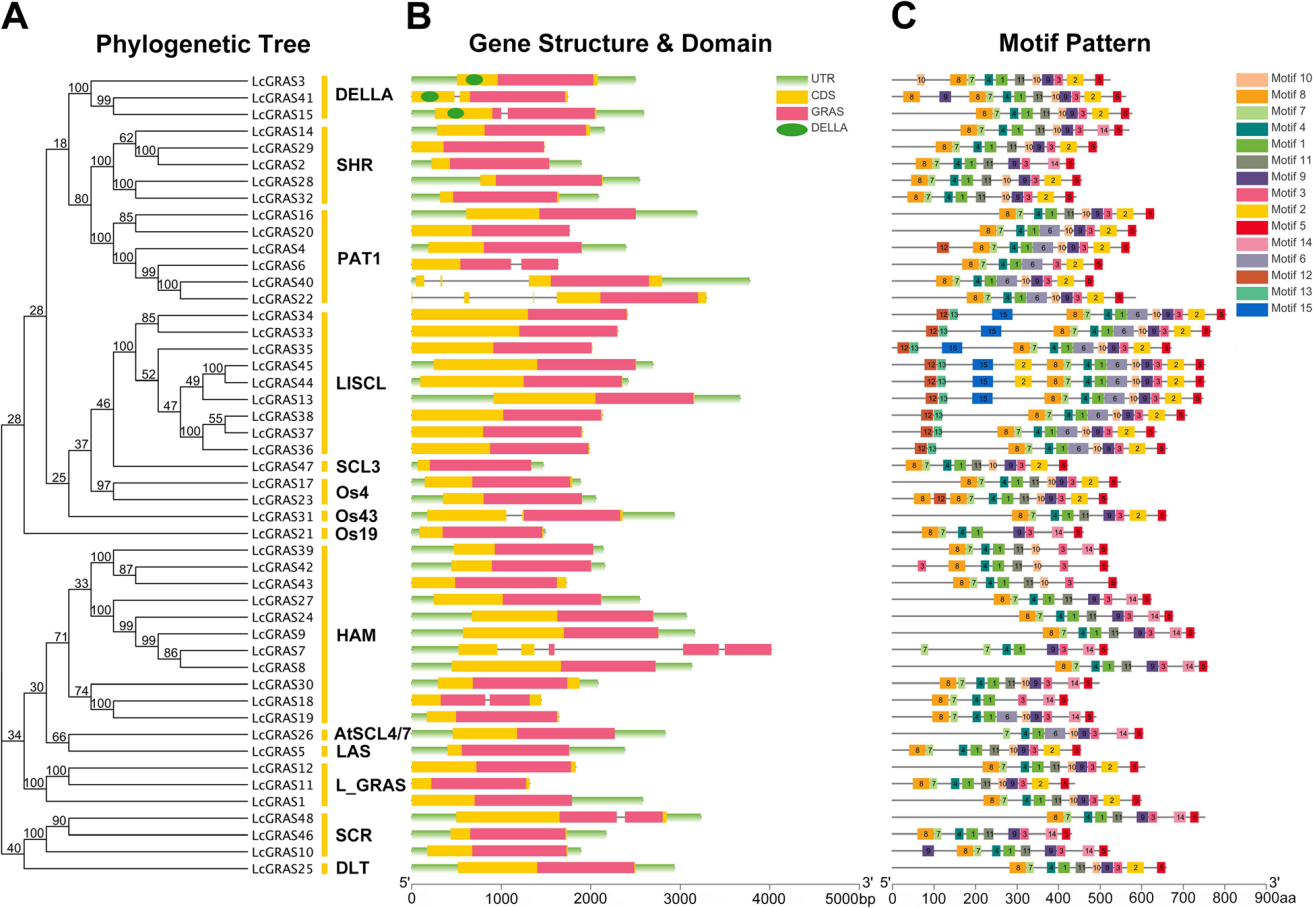
Phylogenetic relationship, exon–intron structure, conserved domains and motif pattern of LcGRAS proteins (genes). **a** Phylogenetic relationship among the litchi GRAS proteins. The unrooted tree was generated using the maximum-likelihood method. The reliability was assessed using 1000 bootstrap replicates. **b** Exon-intron structure and conserved domain regions of LcGRAS genes. Gray lines indicates the position of introns. Information of exon, intron, and functional domain was obtained from model gene annotation and results of NCBI Batch-CD search and visualized by TBtools. GRAS: GRAS domain; DELLA: DELLA domain; bp: base pair. Lengths of exons and introns and domains of each LcGRAS protein were exhibited proportionally. **c** Motif pattern of LcGRAS proteins. aa: amino acid

To further understand the composition of *LcGRAS*s, the gene structures of them were compared. 81.3% of the *LcGRASs* were intronless, only nine *LcGRAS* members had one or more introns (Fig. 2a, b). All LcGRAS proteins incorporated the GRAS domain. Among them, three members (LcGRAS3, LcGRAS15, LcGRAS41) occupied a DELLA domain, which was essential for GA signal perception. A total of 15 distinct conserved motifs (named motif 1–15) were identified in our motif analysis and almost all LcGRAS proteins contain motif 1, 3, 4, 5, 7, 8, 9, 10, 11, 14 (Fig. 2a, c). Interestingly, motif locations exhibited subfamily specific patterns. For example, motif 6 only existed in PAT1, LISCL, HAM, and AtSCL4/7 subfamilies, while motif 12, 13, and 15 were only located in the N-terminal of the members in LISCL subfamily (Fig. 2a, c). In general, different subfamilies embraced various structure compositions, suggesting their great functional diversity.

### Chromosomal distribution and synteny analysis of *LcGRAS* genes

**Fig. 3:**
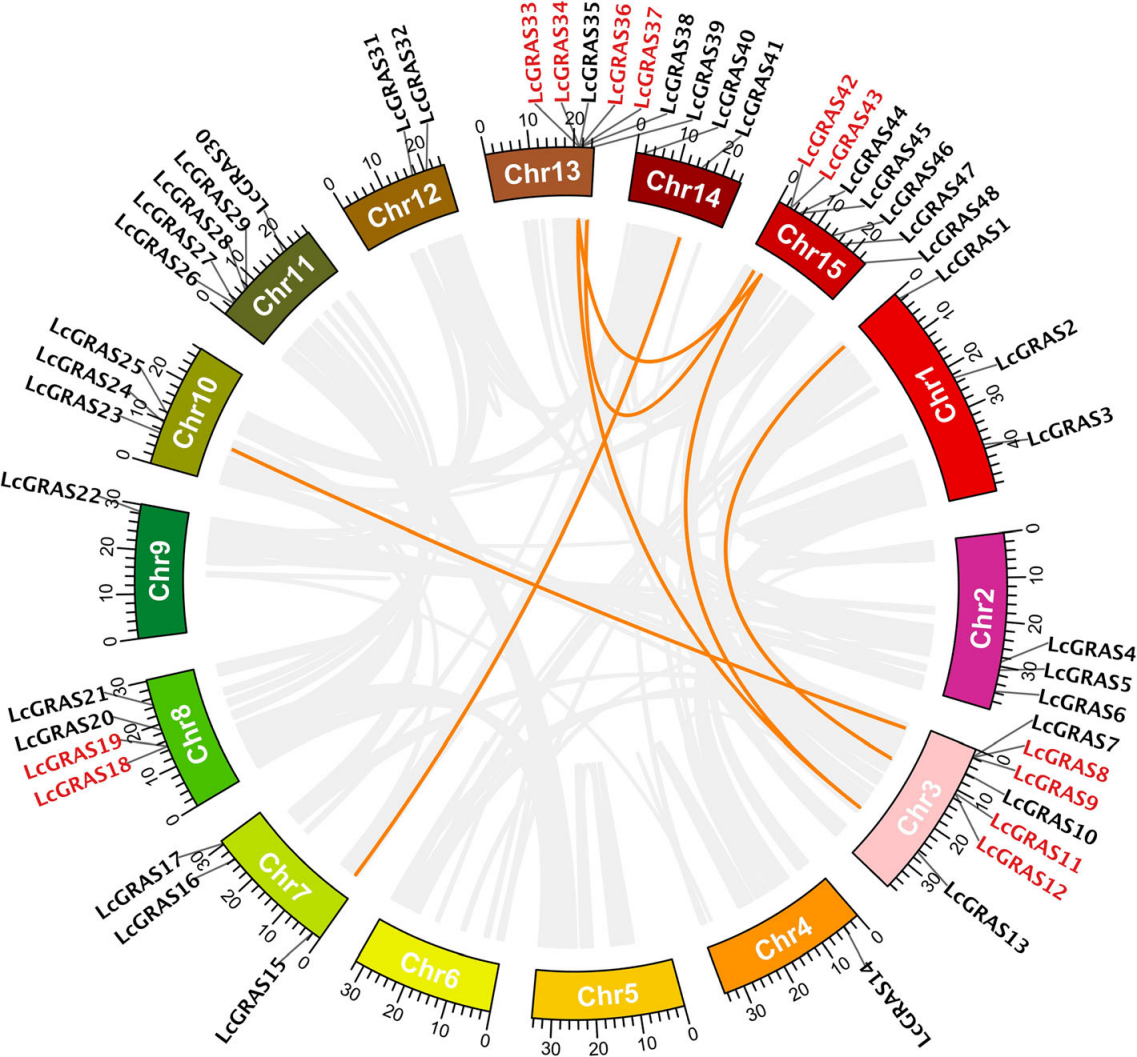
Genomic positions, duplication events and syntenic relationships of *LcGRAS* genes. Distribution of GRAS family genes on each chromosome in litchi. *LcGRAS* genes likely resulted from tandem duplication events are highlighted in red, while those derived from segmental duplication events are connected by orange lines. Gray lines represent syntenic blocks in litchi genome

*LcGRAS* genes were unevenly distributed on the 15 chromosomes of litchi (Fig. 3). There are 7 *LcGRAS* genes located in Chr3, Chr13, and Chr15 respectively, followed by 5 in Chr11 and 4 in Chr8. Four chromosomes (Chr1, Chr2, Chr7, Chr10) had three *LcGRAS* loci, while two of them (Chr12, Chr14) contained two *LcGRAS* genes, respectively. Chr4 and Chr9 possessed only one *LcGRAS* loucs (Fig. 3). Gene duplication contributed to the amplification of the *LcGRAS* family. Tandem duplication (highlighted in red in Fig. 3) was presented in Chr3, Chr8, Chr13 and Chr15, indicating that they were hot spots for *LcGRAS* gene distributions (Fig. 3). Moreover, seven pairs of segmental duplication genes (orange lines) were detected between chromosomes: Chr1/Chr3, Chr3/Chr10, Chr3/Chr15, Chr3/Chr13, Chr7/Chr14 and Chr13/Chr15 (2 pairs) (Fig. 3). Tandem and segmental duplication events of *LcGRAS* genes occurred mainly in HAM, LISCL, DELLA, and L_GRAS subfamilies. Taken together, these results suggested that tandem and segmental duplication may have been the main driving force of the evolution of the litchi GRAS family.

**Fig. 4:**
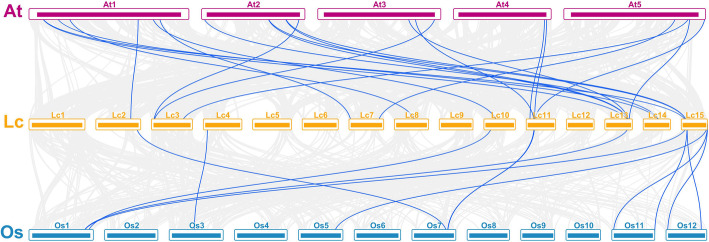
Synteny relationship of the GRAS genes from litchi, *Arabidopsis*, and rice. At1-At5, Lc1-Lc15, and Os1-Os12 represents five chromosomes of Arabidopsis, 15 chromosomes of litchi, and 12 chromosomes of rice, respectively. Gray lines in the background indicate the colinear blocks between three plant genomes (litchi, *Arabidopsis*, and rice), while blue lines highlight the syntenic *GRAS* gene pairs in three species (litchi, *Arabidopsis*, and rice)

To further deduce the relationship of the *LcGRAS* genes, we checked their synteny with *GRAS* genes from a dicotyledonous plant (*A. thaliana*) and one monocotyledonous plant (*O. sativa*) (Fig. 4). A total of 13 *LcGRAS* genes showed syntenic relationships with *AtGRAS* genes, and 8 of them had syntenic loci in rice. *LcGRAS28*, *LcGRAS29*, *LcGRAS33*, and *LcGRAS44* had syntenic loci in both *Arabidopsis* and rice (Fig. 4, Additional file 1: Table S3). These four genes belonged to either the SHR (*LcGRAS28*, *LcGRAS29*) or the LISCL (*LcGRAS33*, *LcGRAS44*) subfamily, hinting their conserved biological function in plants.

### Analyses of miRNA targeting *LcGRAS* genes

**Fig. 5:**
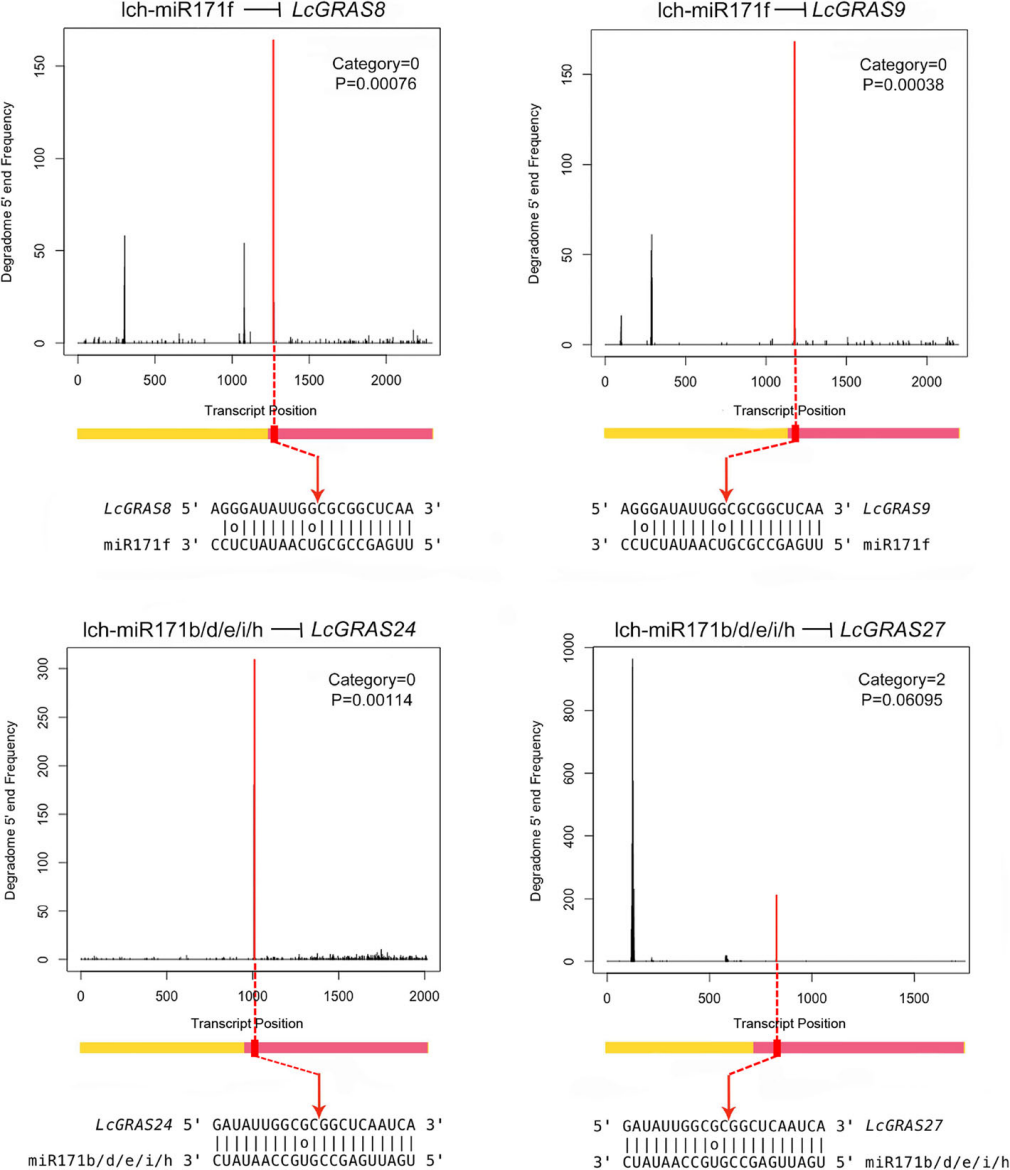
Target plots (t-plots) of identified miR171 targets in litchi using degradome sequencing. T-plots from degradome data were shown in each panel, red lines indicate signatures consistent with miRNA-directed cleavage. The red vertical arrows point to the predicted cleavage sites. P: *P*-vaule. The yellow and pink color indicate the CDS region and the GRAS domain of the gene, respectively

microRNAs are crucial regulatory factors in plants. They regulate the expression of target genes at post-transcriptional level [40]. By combining analyses of degradome data sets from four different libraries, eight miRNAs might have the potential to regulate *LcGRAS* genes (Additional file 1: Table S4). Target genes with penalty score less than 5 and category less than 2 were considered confident [41]. Thus, in total four members (*LcGRAS8*, *LcGRAS9*, *LcGRAS24*, *LcGRAS27*) of *LcGRAS* genes were identified as targets of miR171 (Fig. 5, Additional file 1: Table S4). The miR171-mediated cleavages were verified using degradome data, as presented in the form of target plots (t-plots), showing the abundance of cleaved tags relative to their positions in the transcripts. For each miR171 targeted *LcGRAS* genes, a clear cleavage was detected at the target site of lch-miR171s (Fig. 5). All miR171 target sites were located at the fore end of the GRAS domain (Fig. 5), and all miR171 targeted *LcGRASs* belonged to the HAM subfamily, which were supposed to function in meristematic cell development, root length, and flowering [42–44].

### Expression analysis of *LcGRAS* genes in different tissues

**Fig. 6:**
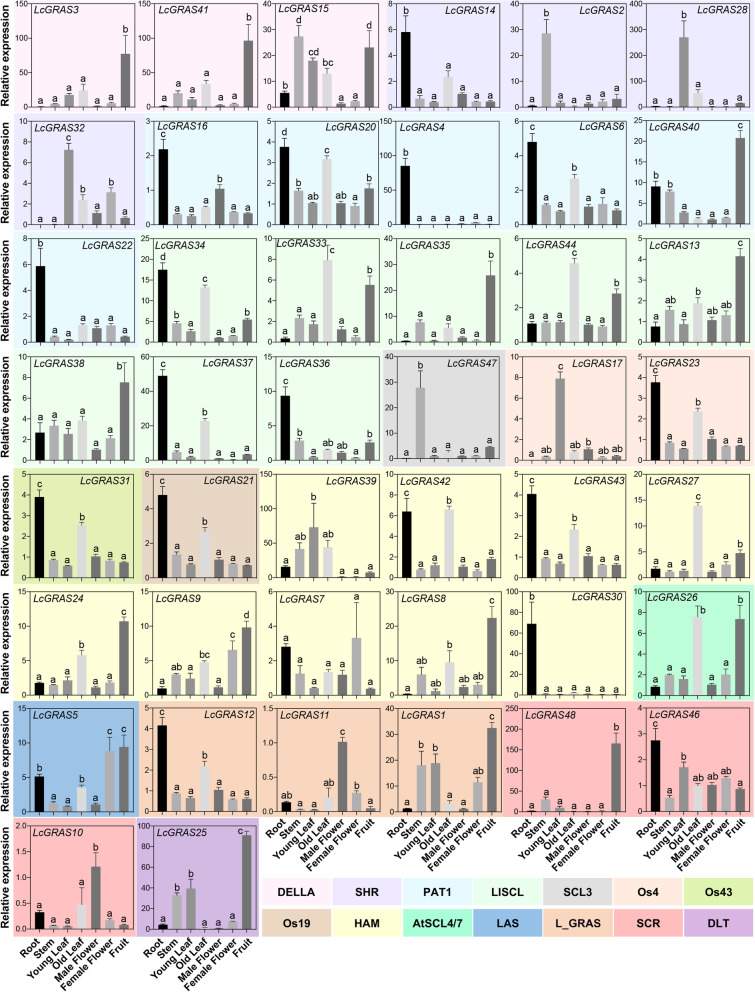
Relative expression of LcGRASs in seven tissues. Data from three independent biological replicates are shown with standard error (SE). Different letters above the bars represent significant differences (*P* < 0.05, LSD) among seven tissues. The control used in the calculations was male flower. The same background color represents members of the same subfamily

To investigate the role of these *LcGRAS* genes, RT-qPCR was used to analyze the expression pattern of 48 *LcGRAS* genes in seven tissues, including root, stem, young leaf, old leaf, male flower, female flower, and fruit. As illustrated in Fig. 6, expression of 44 *LcGRAS* genes was obtained, while four *LcGRAS* genes cannot be detected because of their extremely low expression levels. Most of the *LcGARS* genes were highly expressed in root and old leaf and poorly expressed in either male or female flowers. In addition, most genes in *LISCL* were highly expressed in fruit and old leaf, while some genes were with rich expression in root, suggesting that functional diversification was present in this subfamily. *LcGRAS46* in SCR, *LcGRAS14* in SHR and almost all genes in PAT1 subfamily were abundant in root (Fig. 6); genes in DELLA subfamily (*LcGRAS3*, *LcGRAS15*, *LcGRAS41*) were all highly expressed in fruit; *LcGRAS2* (SHR) and *LcGRAS47* (SCL3) were found to have higher expression in stem (Fig. 6). We also found that gene *LcGRAS11* which belongs to litchi-specific subfamily L_GRAS, was highly expressed in male flower (Fig. 6), demonstrating it would be closely related to the male flower development. In contrast, *LcGRAS5*, the sole member of the LAS subfamily, was highly expressed in female flower and fruit (Fig. 6), indicating its function in the development of female flowers and fruits. Moreover, *LcGRAS25* (DLT) was of high-level expression in fruit, stem and young leaf (Fig. 6), implying its potential function in fruit, stem, and young leaf development.

### Expression profile of *LcGRAS* genes in two varieties with contrasting seed size

**Fig. 7:**
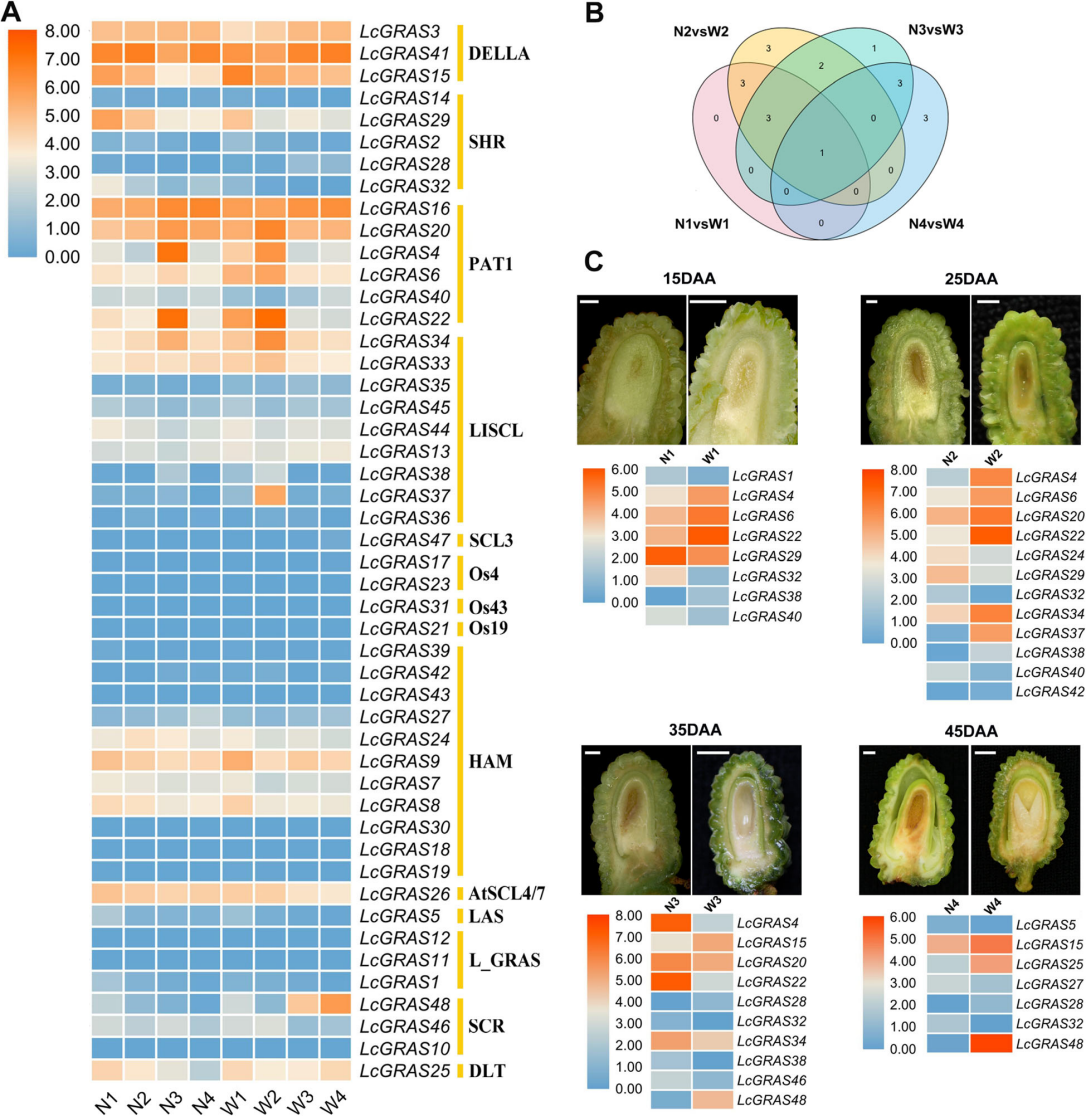
Differential expression of *LcGRAS* genes between the abortive and bold seeded litchi. The heatmap was created based on the FPKM values of LcGRASs from the transcriptome data. In the heatmap, orange and blue were represented higher and lower expression (log2 (FPKM+ 1)), respectively. **a** Expression pattern of *LcGRAS* genes in both abortive and bold-seeded litchi at four development stages (N: ‘NMC’, W: ‘WL10’, 1: globular-shaped embryo stage, 2: heart-shaped embryo stage, 3: torpedo-shaped embryo stage, 4: cotyledon embryo stage). **b** Venn diagram showing number of differentially expressed *LcGRAS* genes (log_2_FC > 1; Padj < 0.01). **c** Differential expression profile of *LcGRAS* genes between abortive and bold-seeded litchi at the four developmental stages. Scale bar in figures of 15DAAand 25DAA: 1 mm; Scale bar in figures of 35DAAand 45DAA: 2 mm

To explore the character of *LcGRASs* in the regulation of seed development in litchi, transcriptome analysis was conducted on a litchi cultivar (‘NMC’) and a wildtype litchi (‘WL10’), representing developing small (abortive) and large (bold) seeds, respectively (Fig. 7a, Additional file 1: Table S5). Seed samples at four developmental stages, including globular-shaped embryo stage (15 DAA), heart-shaped embryo stage (25 DAA), torpedo-shaped embryo stage (35 DAA), and cotyledon-shaped embryo stage (45 DAA), were collected for RNA sequencing. Pairwise comparison of the developing seeds unmasked the common and exclusive differentially expressed transcripts at 15, 25, 35, and 45 DAA between the two varieties. Among 48 *LcGRAS* genes, 8, 12, 10 and 7 *LcGRAS* genes were differentially expressed (‘NMC’ vs ‘WL10’) at 15, 25, 35, and 45 DAA, respectively (Fig. 7b, c, Additional file 1: Table S6). Notably, *LcGRAS32* was consistently high expressed in abortive-seeded cultivar (‘NMC’) during all four stages (Fig. 7b, c), which suggested its potential function in embryo abortive development, giving rise to small seed. The seed development process in litchi could be divided into the cell division stage and the filling stage around 28 DAA when the embryo reached the heart-shaped embryo stage with a rudimentary cotyledon. The cell division stage before 28 DAA was more important for normal seed development [45]. Intriguingly, *LcGRAS29* and *LcGRAS40* were exclusively high accumulated in small-seed during both globular-shaped embryo stage (15 DAA) and heart-shaped embryo stage (25 DAA), implying their possible function in seed abortion. In addition, *LcGRAS1* was specifically highly expressed in globular-shaped embryo stage (15 DAA) while *LcGRAS24* was specifically highly expressed in the heart-shaped embryo stage (25 DAA) (Fig. 7c), indicating that they may be involved in cell division in seed development as well. Furthermore, *LcGRAS15*, *LcGRAS28* and *LcGRAS48* were of high expression in ‘WL10’ (bold-seeded) during torpedo- and cotyledon-shaped embryo stages, suggesting these genes may be linked with important traits during the filling stage of normal seed development (Fig. 7c).

## Discussion

*GRAS* gene family has been characterized in several plant species, and involved in numerous critical development and physiological processes. In our study, 48 *LcGRAS* genes were identified in litchi (Fig. 1). The population of *LcGRAS* members was larger than that in *Arabidopsis* (33) [46], and roughly the same to that in *P. mume* (46) [7], tomato (*S. lycopersicum*) (53) [11], castor bean (*R. communis*) (48) [16], and pepper (*C. annuum* L.) (50) [10], but less than in *Populus* (106) [14], rice (57) [6], and cotton (*Gossypium hirsutum* L.) (150) [18], implying extensive duplication and diversification of the *LcGRAS* gene family among species. In the analysis of the structural compositions of *LcGRASs*, we found 81.3% of *LcGRASs* were intronless (Fig. 2), which was similar to tomato (77.4%) [11] and *P. mume* (82.2%) [7]. Intronless genes have been discovered in gene families DEAD box RNA helicase [47] and F-box gene family [48]. The high proportion of intronless genes in litchi suggests that they may have experienced intron loss events during evolution, which is common in other eukaryotes [49]. Tandem and segmental duplication are the main mechanisms for the expansion of plant gene families [50] and play a crucial role in the adaptive response to environmental stimuli [51]. Tian and colleagues [6] analyzed the expansion mechanism of *GRAS* gene families in *Arabidopsis* and rice. In litchi, six tandem duplication and seven segmental duplication gene pairs were found (Fig. 3), these gene pairs in HAM, LISCL, L_GRAS and DELLA subfamily are like other plant species [52]. Our result indicated that duplication events may be a mechanism for expanding the number of *GRASs* in these subfamilies. In addition, *LcGRAS1*, *LcGRAS11*, and *LcGRAS12* of L_GRAS subfamily, a new subfamily identified in litchi, were also experienced the two types of duplication events. These L_GRAS genes may possess special function for the growth and development of litchi, which need further researches. Moreover, some gene pairs of the two duplication events had similar expression pattern in different tissues, as shown in Fig. 6, which also existed between *PmGRAS16* and *PmGRAS26* in *P. mume* [7].

The analysis of expression patterns can facilitate our depiction of the potential functions of *GRAS* genes [53, 54]. Genes in maize (*ZmSCR*) and rice (*OsSCR)* were shown to have similar expression patterns to *AtSCR* in roots [55, 56], and these two *GRAS* members (SCR and SHR), were involved in several different stages of (root) development [57]. GRAS protein from the PAT1 clade was shown to be associated with the development of the adventitious and lateral root [58, 59]. HAM clade of the GRAS family was vital for root development but involved in leaf development, with a triple-mutant (*scl6*, *scl22*, and *scl27*) leading to reduced root growth and abnormal leaf patterning [44, 60]. In our study, four *LcGRAS* genes were undetectable in all tissues, suggesting a trend to degenerate these genes after gene duplication or the loss of gene functions during evolution. Most of the *LcGRAS* genes were highly expressed in root, including members in SCR, SHR, PAT1, and HAM subfamily, indicating they might function in root development in litchi. Moreover, GRAS protein from the HAM subfamily was supposed to participate in the vegetative to the reproductive phase transition by activating the miR156-*SPLs* pathway [42, 61]. *LcGRAS8*, *LcGRAS*9, *LcGRAS*24, *LcGRAS*27 from HAM subfamily were identified as the targets of miR171 (Fig. 5), and they shared a similar expression pattern, suggested that the relatively conserved functions of the miR171-GRASs regulatory networks in litchi. The four genes especially highly expressed in both old leaf and fruit, which implied their potential roles in leaf patterning, flower organ formation [42] and fruit development in litchi [62–64]. Additionally, several genes were prominently with higher expression in fruit, including three DELLA genes (*LcGRAS3*, *LcGRAS15*, *LcGRAS41*), and *LcGRAS25* in DLT subfamily, which might be involved in the fruit development through GA signal transduction pathway [65–68] or brassinosteroid signal transduction pathway [69, 70], respectively.

GA and auxin are prominently associated with the seed formation during fruit development [71–73]. Moreover, some GRAS proteins function as regulators of auxin and GA in plant development, such as fruit and seed development. For instance, the overexpression of *SlGRAS7* enhancing GA/auxin signaling and improving resistance to abiotic stresses [74]. *SlGRAS24* was characterized to impact multiple agronomical traits by regulating auxin and GA homeostasis in tomato [34]. Additionally, it is evident that *SlGRAS40* acted as a regulator of auxin and GA as the overexpression of *SlGRAS40* led to auxin insensitivity and GA deficiency [75]. Moreover, overexpresseing *SlGRAS24* or *SlGRAS40* in plants would lead to pleiotropic phenotypes such as reduced fruit set ratio, arrested fruit, and abnormal seed development [34, 75]. *SlGRAS24* and *SlGRAS40* were the target genes of miR171 and belonged to HAM subfamily in tomato. Similarly, in litchi, there were 4 genes of HAM subfamily targeted by miR171, which were higher expressed in fruit, indicating miR171-*GRAS* regulatory pathway might play similar roles in seed and fruit development like *SlGRAS24* and *SlGRAS40* through GA signal transduction pathway. Expect that, in the *SlDELLA* deficit model, the tomatoes exhibited GA insensitivity and displayed a GA-constitutive response phenotype, including parthenocarpy [66, 68]. In our study, three DELLA genes (*LcGRAS3*, *LcGRAS15*, and *LcGRAS41*) were higher expressed in fruit, indicating their potential functions in fruit development through GA pathway. *PrSCL1* (*Pinus radiata* SCL1) and *CsSCL1* (*Castanea sativa* SCL1) were shown to regulate adventitious root formation through auxin signaling [76]. In *Arabidopsis*, the collaboration between the SHR-SCR complex and auxin influx carriers (LAX3 and AUX1) could lead to synergistic effect on primary/lateral root development [77]. In pine and cucumber, relatively high expressing *GRAS* transcripts, such as SCR and SHR, were measured in non-differentiated proliferating embryogenic cultures and during embryo development [15, 78]. In our study, *LcGRAS14* in SHR was highly expressed in root, and *LcGRAS48* was highly expressed in fruit, indicating that *LcGRAS* members might function in root and seed development by participating in auxin signal pathway. Hence, based on their similar expression pattern in different tissues and similar conserved domain, *LcGRASs* that belong to HAM, DELLA and SHR were supposed to be involved in different developmental processes via crosstalk with GA or auxin signaling.

‘NMC’ and ‘WL10’ are two litchi varieties that display remarkable difference in seed size after maturity. ‘WL10’produces larger seeds with normally developed embryos and cotyledons, while ‘NMC’, as the seed-aborting cultivar, produces seeds with defect embryos or cotyledons. It has been reported that 28 DAA represented a transition point between the cell division stage and the filling stage during litchi seed development, after which sequential liquid endosperm and embryo development were not observed in ‘NMC’ [45]. In our result, *LcGRAS29* (SHR), *LcGRAS40* (PAT1), *LcGRAS1* (L_GRAS) and *LcGRAS24* (HAM) were exclusively and highly accumulated in ‘NMC’ (abortive seed) before 28 DDA (Fig. 7c), indicating its exceptional function in endosperm and embryo abortion of litchi. In addition, *LcGRAS15* (DELLA), *LcGRAS28* (SHR) and *LcGRAS48* (SCR) were up-regulated in ‘WL10’ (bold-seed) at 35DAA and 45DAA (Fig. 7c), which suggested that they may work in later seed maturation. These genes might participate in seed development by regulating auxin and GA pathways.

## Conclusions

In this study, 48 *LcGRAS* genes were identified in litchi and divided into 14 subfamilies. Members of the same subfamily have similar gene structures. Some *LcGRAS* genes are derived from gene duplication. The expression patterns of *LcGRAS* genes in different tissues were diverse, indicating that they might have different functions during the development of litchi. Four *LcGRAS* genes were regulated by miR171 directly. In addition, our result indicated *LcGRAS* genes are differentially expressed in different varieties of litchi (‘NMC’ and ‘WL10’) and illustrated crucial roles of *LcGRAS* proteins in embryos or cotyledons development which affects seed size. This research was the first comprehensive identification of *LcGRAS* genes in litchi. These results provide the foundation to elucidate the regulation mechanism of *LcGRASs* in plant growth and seed size, showing that *LcGRASs* might have important functions in litchi breeding.

## Methods

### Plant materials preparation

Three 13-year-old ‘Huaizhi’ litchi (one of the main cultivars in China) trees used in our study were planted in the orchard located at South China Agricultural University (Guangzhou, China). Different tissues, including root (root tips approximately 10 cm long), stem, young leaves (leaves approximately 3 cm long with yellow or light green color, and the tip of the leaves is red), mature leaves (green but not leathery leaves), male flower (full bloom), female flower (full bloom), and young fruit (31 DAA) were collected for RT-qPCR analyses. The 30-year-old ‘NMC’ (cultivar litchi with abortive seeds) and ‘WL10’ (wildtype litchi with bold seeds) used in this study were grown in the germplasm resource orchard of Guangdong Province Fruit Research Institution (Guangzhou, China). Seed samples of ‘NMC’ and ‘WL10’ used in RNA-seq analysis were collected in different developmental stages (15, 25, 35, and 45 DAA) from two randomly selected trees. All samples were collected separately from three trees with similar growing conditions, and then quickly frozen in liquid nitrogen and stored at − 80 °C.

### Identification and protein property analysis of LcGRASs

The Gtf/ Gff3 Sequence extractor in TBtools V1.046 [79] was used to extract the coding sequences (CDS) of all *GRAS* genes from a reference litchi (‘Feizixiao’) genome of 15 pseudo-chromosomes (470 Mb) with 96.2% completeness (assembled in house, data unpublished yet) based on the gene structure annotation information, and then CDS sequences were translated into protein sequences using Batch Translate CDS to protein tool in TBtools V1.046 [79]. Thirty-three GRAS protein sequences from *Arabidopsis* were downloaded from TAIR (https://www.arabidopsis.org/browse/genefamily/gras_genefamily.jsp) [5], which were used as baits to identify potential GRAS genes in the litchi genome by BLAST analysis with a relative sensitive cutoff (E-value set at 1e-5) in TBtools V1.046 [79], the resultant protein sequences were then used as queries to search against the UniportKB/Swiss-port (swissport) databases using the BLASTP program with default parameters to avoid false positives. The identified sequences were then validated using CDD (http://www.ncbi.nlm.nih.gov/cdd/) [80] with E-value threshold 0.01 and Pfam (http://pfam.xfam.org/) [81] databases with default parameters. The ProtParam tools from the ExPASy website (https://web.expasy.org/prot-param/) [82] were used to obtain the sequence length, predicted molecular weight, and predicted isoelectric point of the identified GRAS proteins.

### Phylogenetic analysis of LcGRASs, AtGRASs, and OsGRASs

GRAS genes of *Arabidopsis thaliana* and rice together with the litchi GRAS genes were sued in phylogenetic analysis. Thirty-three GRAS proteins from *Arabidopsis thaliana* and 60 GRAS proteins from rice (Additional file 1: Table S1) were downloaded from TAIR [5] and PlantTFDB V5.0 (http://planttfdb.cbi.pku.edu.cn/) [83] respectively. AtSCL16 (a putative pseudogene [46]) and some members in rice (protein length is less than 350 aa [11]) were excluded in the subsequent analysis. Multiple protein sequence alignment was carried out via Muscle [84], and the poorly aligned regions were removed by TrimAL 1.3 in TBtools V1.046 with default parameters [79]. Phylogenetic analysis was performed by MEGA7.0 program by maximum likelihood (ML) method and the bootstrap test was carried out with 1000 iterations [85].

### Gene structure, domain, and conserved motif analysis

Introns and exons of each LcGRAS gene were analyzed using TBtools V1.046 [79]. The conserved domains were defined using the Batch-CD search (http://www.ncbi.nlm.nih.gov/Structure/bwrpsb/bwrpsb.cgi) [86, 87] with default parameters. The MEME-Suite 5.1.1 online program (http://meme-suite.org/) [88] was used to analyze the conserved motifs to investigate the structural differences among LcGRAS members. All results above were visualized by TBtools V1.046 [79].

### Chromosomal distribution and gene duplication of LcGRAS genes

The physical location information was obtained from the litchi gff3 file and plotted by TBtools V1.046 [79]. Multiple collinear scanning toolkits (MCScanX) with default parameters were used to analyze gene duplication events [89]. The syntenic relationship between *LcGRASs*, *AtGRASs*, and *OsGRASs* was determined using MCScanX and visualized by multiple synteny plot tool in TBtools V1.046 [79].

### Identification of transcripts targeted by miRNAs

Six sRNA and four degradome data sets of litchi (*L. chinensis* Sonn.) were downloaded from accession number GSE98698 which were stored in NCBI [90]. An in-house software, sRNAminer, was used to monitor quality, trim adaptor, and collapse reads with the same sequence of sRNA sequencing data [91]. Subsequently, noncoding RNAs (including rRNA, snoRNA, and tRNA) and sRNAs from chloroplast and mitochondrial genome were removed by mapping against RNA Family (Rfam) database V13.0 [92, 93] and the Plant organelles database [94] via bowtie [95] respectively. Preprocessed reads were mapped to the litchi genome and used to explore miRNAs. Cleveland 4.0 was adopted to identify transcripts targeted by miRNAs and authentic target sites with a confident level of category 0–2 and penalty score no more than 5 [96] were screened. All degradome reads on cleave sites were normalized to reads per 10 million (RPTM).

### Expression analysis of LcGRAS genes by RT-qPCR

Total RNA was extracted using the Hot borate method described by Wan and Wilkins [97], the cDNA strand was synthesized with the HiScriptII Q RT SuperMix for qPCR (+gDNA wiper) (Vazyme Cat No. R223-01). RT-qPCR was performed with GoTaq® qPCR and RT-qPCR Systems (Promega Cat No. A6001) using a Light Cycler 480 Real-Time PCR Detection System (Roche, Rotkreuz, Switzerland). Primers of *LcGRASs,* and two reference genes *GAPDH* and *EF* [98] were designed by Primer Premier 5.0 (Additional file 1: Table S7). Each expression profile was independently verified in three biological replicates. The relative expression level of each gene was calculated by the 2^-△△Ct^ method [99].

### RNA-seq and differential expression analysis

The transcriptomic data were generated from different seed development stages (15, 25, 35, and 45 DAA) of two species of litchi (‘NMC’ and ‘WL10’). Trimmomatic software was used to control the quality of raw RNA-seq data and remove the adapter [100]. Afterwards STAR software was used to map clean data to the litchi genome and the expression level of transcripts was normalized into fragments per kilobase of transcript per million fragments mapped (FPKM) by StringTie [101, 102]. Differentially expressed genes were identified using an R package, DESeq2 [103], where adjusted *P*-value (Padj) < 0.01 and foldchange > 2 were set as thresholds. In detail, we took the average of the two biological replicate counts of each sample, and then divided the average counts of all the two sets of samples to be compared with each other to get the fold change value (FC). Lastly, took the logarithm of 2 for the obtained fold change, next got log2FC (log2 fold change). If the log2FC value of a gene was greater than 1, and the Padj was less than 0.01, the gene would be significantly up-regulated. Correspondingly, if the log2FC value was lower than - 1, and the Padj was less than 0.01, the gene was considered to be significantly down-regulated.

## Supplementary Information

**Additional file 1: Table S1.** GRAS proteins of litchi, *Arabidopsis*, and rice. **Table S2.** Information of *LcGRAS* genes. **Table S3.** Syntenic gene pairs among litchi, *Arabidopsis* and rice. **Table S4.** Information of miRNA targets in litchi GRAS gene family. **Table S5.** Gene expression profile of 48 *LcGRAS* members among four seed development stages (Normalized as FPKM). **Table S6.** Information of differentially experssed *LcGRAS* genes. **Table S7.** Specific primers of 48 *LcGRAS* genes used for qPCR in this study.

**Additional file 2.** Sequences of litchi *GRAS* genes.

## Data Availability

Six sRNA and four degradome data sets of litchi (*L. chinensis* Sonn.) were available from accession number GSE98698 which were stored in NCBI. The litchi genome data and RNA-seq data that support the findings of this study have been deposited into CNGB Sequence Archive (CNSA, https://db.cngb.org/cnsa/) of China National GeneBank DataBase (CNGBdb) with accession number CNP0001024 and CNP0001865 respectively, which will be released after the publication of the related paper. Review links are available as https://db.cngb.org/cnsa/project/CNP0001024/reviewlink/ and http://db.cngb.org/cnsa/project/CNP0001865/reviewlink/ respectively. Other data sets supporting the results of this article are included within the article and its additional files.

## Abbreviations

GAI: Gibberellic-acid insensitive
RGA: Repressor of GAI
SCR: Scarecrow
aa: Amino acids
GA: Gibberellic acid
JA: Jasmonic acid
DAA: Day after anthesis
MW: Molecular weight
pI: Isoelectric point
T-plots: Target plots
CDS: Coding sequences
RPTM: Reads per ten million
FPKM: Fragments per kilobase of transcript per million fragments mapped
Padj: Adjusted *P*-value
FC: Fold change
