# Supplementary material for: The GRAS gene family and its roles in seed development in litchi (Litchi chinensis Sonn)
Source: BMC Plant Biol. 2021 Sep 17;21:423. doi: 10.1186/s12870-021-03193-1 (PMC8447652; doi:10.1186/s12870-021-03193-1)
Supplement: Supplementary file 2 — Additional file 2. Sequences of litchi GRAS genes. [file 12870_2021_3193_MOESM2_ESM.docx]

Additional file 2: Sequences of litchi *GRAS* genes

>LcGRAS41

ATGAAGAGAGAGCATCAGCAGATGGAGCAGACGTCGGGTGGGTCGAGTGCGAGTGGTAAAGGGAAGATGTGGGAAGAGGAGGCGTGTGACGGAGGGATGGATGAGCTTCTTGAGGTTCTAGGGTACAAGGTGAGGGCGACGGACATGGCGGATGTTGCTCAAAAGCTTGAACGGCTTGAAGAAGTGATGGGCAGTTTTCAAGAAGATGGTATCTCTCATCTGGCTTCTGAAACTGTCCATTACGATCCGTCCAATTTGTCAACTTGGCTCCAATCCATGCTCTCCGAGCTTAACCCGGATCCGATTCCAGCACCCGCCCCTGTTCCTTCCTTGGATCCTGATTCCACCATCACCACCTTCATCTCTACTGCTGAACCCTCCACCATGACCACCTCCATCGATTTCTCAAGCCATCAGCGTCTGATCGATGGATCCTGTACATCTGACTACGATCTCAAAGCCATCCCTGGCAAGGCCATGGACCAAAAACGCTTGAAAACCTCTCCCGACATGTACCCACCTGAGTCAACTCGTTCGGTCGTGCTAGCCGACTCACAGGAAAACGGAATCCGACTGGTCCACGCATTGATGGCGTGCGCGGAGGCTGTCCAGCAGCACAACCTGAGCTTAGCGGAGGCCTTCGTGAAGCAGATTCGGTTCCTGGCGATGTCTCAAGCGGGCGCGATGGGGAAGGTAGCCACCTACTTTGCCGAAGCATTGGCTCATAGAATTTACGGGCTCTACCCTCAGGAACCCATCGACCACAACTTCACAGATCTTCTCCAGATGCACTTTTACGAGACTTGTCCTTACCTCAAATTCGCTCACTTCACTGCGAATCAAGCCATTCTCGAGGCCTTTGAAGGTAAAAAACGTGTTCATGTCATAGATTTCTCGATGAATCAGGGCATGCAGTGGCCGGCTCTAATGCAGGCCTTGGCTCTCCGGCCTGGTGGGCCGCCGGCCTTTAGGCTGACCGGAATCGGGCCGCCGGCGGCTGACCACACCGACCATTTGCAGGAAGTGGGTTGGAAGTTGGCTCAGTTGGCTGAAACCATTCATGTTGAGTTTGAGTATAGAGGGTTTGTAGCCAACAGCTTGGCTGATCTCGATGCCTCCATGTTGGAACTCAGGCCCACCGAGGTCGACTCGGTTGCTGTCAACTCCGTCTTCGAGTTGCACAAACTTTTGGGTAGACCGGGTGCCATTGACAGAGTGTTTTCTGTTGTCAAGGACATTAGGCCCGACATTTTCACTGTCGTCGAGCAGGAAGCCAACCACAACGGTCCGATTTTCCTCGACCGGTTCACGGAGTCACTTCATTATTACTCCACCATGTTTGACTCGCTCGAAGGATCGGTCAACACCCCGGACAAGGCCATGACGGAGGTTTACTTGGGCAAGCAGATTTGCAACGTTGTCGCCTGCGAAGGTATGGACCGGGTCGAAAGGCACGAAACGCTCACTCAGTGGAAAGCCCGGTTCGGCCCGGCCGGATTCGCCCCGGTTCATCTGGGGTCGAACGCTTATAGACAAGCGAGCATGTTGCTGGCGCTGTTCGCGGGCGGGGAAGGGTACAGGGTGGAAGAGAACAACGGATGTCTGATGTTGGCATGGTACACTCGGCCGCTCATCGCCACCTCGGCTTGGAAACTCGCCCACAAACCGCTGGTCGTTTCTCAGTGA

>LcGRAS27

ATGCACATATACACACGCAAAGATAAAAAGCCCGCAATCATAACCCCGAAAAGCCACTCCTTCTCTCCCCACCACGCTTTCAAGCAATCAACAACCAAAGAGAGAGCTCTATTGGCTACTTTCTCTCTCTCTCTCTCTCTCTCTCTTCTCCAAGCTATGAGAGTTCCTGTTCAACCACCACAAATCGACCAATCTTCCACCCCAAAACCACCTTGCAACGTCAACAACAACAACAACGCCATCAACGTGGCACCACCAAACATAAGCCTGGCAGGTTCGTGTTACGAACCCACTTCGGTTCTTGAACTCCGGAGTCCAACCCCTATACCTGAAAAACCAGCACCCACTGCCACGTCAGACGTGTCAACCGCCGTCGTCCAATCGGACCCTCCTCTGGTTGAGTTGGACGAGCATGCAATTCGTAGCATGGACTGGGATTCCATCATGAGGGAGCTGGGCTTGGATGACGATTCTGCCCTCACTTCCTTCAAGAGTGGTACCAATATCAATATCCATCCTCAGTTGATCACCCGTTGTGACCCTCATCAAATCCAACACAGCCTCAACGAGTTCTCTCCTTATCATCACCATCATCATCATCATCAGCCACCTGATTCCAATTCCCAGTTAATTAATCATTCTGATTTTACTTTTAATGTCCCTGACACGTACTCTAACCAGAACCAAAATTTTGGTCACGTTTTTGACAACATGCCTAGTAACAGCAATAATTGTAACGTGGGTTTGGATTTCATAGACGAACTAATCCGAGCCGCTGACTGTTTCGACTCAAACGAGTTACAACTTGCGCAGATGATATTGGCGCGGCTCAATCAACGTTTAAGAGCACCAGTCGGTAAACCACTTCAACGAGCCGCCTTCTTCTTCAAGGAAGCTCTCAACTCGGTCCTTTACGGGTTAACTCGGCCGATTCGGCTCTCGTCATGGTCCGAAATCGTGCAGTCTATTAGAGCCTACAAGGCTTTCAGTGCTATCTCTCCTATCCCCATGTTCACTCACTTCACGTCCAATCAAGCCCTCCTTGAGTCGTTGGATCGAGATGGAACATCATCGTCACCGTTGATACACATCATAGACTTTGACATCGGATTCGGAGGGCAGTACGCTTCGTTCATGCTAGAAATTGCCGAGAAAGCTGAGTCTTGTAAAATGAATCCGGCGTCTCTTCGAATCACGGCTGTTGTACCAGACGAATACGCAAGTGAAACCAGGCTCATCAAAGACAACCTTATTCACTTCGCGCAGGAACTGAAGATCAGGTTCCACATAGACTTCGTCCTTGTCCGAATCTTCGAGCTGTTCTCTTTCAAAGCAGTCAAGTTCGTGGAAGGTGAGAAGACAGCTGTTGTTTTGTCTCCAGCTATATTTCGCCGCCTAGGTTCGACCAACAACGCCATCGCGTTCGTCTCAGACATAAGGCTGTTGTCGCCGAGCGTCGTCGTGTTCGTCGACAGCGAATGTTCGGCGGAGACGTCCCCAGCGGCGGCGGCGGCAGCCGGGTCTTTCAAGAGGGGCTTCGTGGGCAGTCTCCAGCACTACTTGAGGATGTTCGAGTCTTTGGACGCGGCGATAGGGGGCGGGGACTGGCCGAGGAAGATAGAGATGTCGCTGCTGAGGCCGAGGATAGCGGCTGCCGTGGAGGGGGCGGGGAGGCGGGTGGCGCCGTGGAGGGAGGTGTTTTCTGGGGCTGGGCTGAGGCCGGTGAAGCTGAGTCAGTTTGCGGATTTCCAGGCGGAGTTTTTGCTTGGGAAGGTGCAGGTGGGCGGGTTCCAGGTCGACAGACGCCAAGCTGAGTTGGTGCTTTGCTGGCACCAGTGGATCCTTGTTGCCACGTCAGCTTGGAGGTGTTAG

>LcGRAS29

ATGGATACTTTGTTCAGACTGGTTAGTCTTCAATCCGATCAATCTATCAACTCTAGCAGAACCTCTAGCAGCTCTAGGTCGTCCAGACAGAACAATCACTACCAGCAAGAAGACGAAGAATGCTTCAATTTTTTCATGGATGAAGAAGACTTCTCCTCGTCTTCTTCTAGGCACTACTATCCTTATCATCAACCCCACCCCTCAAACACCACCACTACTCCTACCACGACGACGACCAACACTAGCACCCCTACCCACCAAGCTTTTGAGTCCACTGACTTCTCTTTCTCTCCTGCTCGTGACCTCAATTTCGACTTCTCAGGCAAGTGGGCAACGGATATTCTCATCGAGACTGCACGGGCTATTGCTGATAAGAACAGCGCCCGTGTCCAGCAGTTGATGTGGATGCTCAACGAGCTCAGCTCTCCTTATGGTGATACCGACCAGAAGCTCGCTTACTACTTTCTTCAAGCTTTGTTTAGTAGGATGACCGACTCCGGTGAGCGGTGCTACTCTACTCTAGCTTCTGCTTCGGAGAAAACCTGCTCTTTCGAGTCAACGAGGAAGATGGTATTGAAGTTTCAAGAGGTGAGTCCTTGGACCACTTTTGGACACGTTGCTTCTAATGGTGCAATCATGGAAGCCATTGAAGGCGAAACCAACTTACATATTGTTGATATTAGCAACACTTATTGCACACAATGGCCCACTTTGCTTGAAGCCCTAGCTACCCGCACCGATGAGACACCCCACCTGAGGCTAACCACCGTAGTCACCACCAAGTCTGGCGGCGGTGCCGGCGGCTCAGCTGCTGTTCAAAAGGTAATGAAAGAGATAGGCAACCGAATGGAAAAATTCGCTAGGCTTATGGGTGTGCCTTTTAAATTCAATGTTGTACACCATACCGGTGATTTATGTGATCTAAATTTAGCTGAATTAGATATTAGAAACGACGAAGCGCTAGCTATCAACTGTATCGGTGCTTTGCACACGATCACAGCCGTTGATAATCGTAGAGACATTCTGCTATCGAATTTGAGAAGACTGCAACCGAGAATAATCACTGTCGTTGAAGAAGAAGCTGATCTTGATGTGGGTATTGACGGTCTAGAGTTTGTCAATGGTTTTCAAGAATGTTTAAGATGGTTTAGGGTTTACTTTGAGTCATTAGAAGACAGTTTCTCAAGAACAAGCAATGAACGGTTGATGCTGGAGAGGGCAGCGGGGCGTTCGATCATGGACTTGGTGGCATGCCCGCCGTCCGATTCGATTGAGAGGAGGGAATCCGCGACTCGCTGGTCACAACGTTTGCATGCAGCAGGGTTCAGCCCGAGTGTGTTGAGTGATGAGGTGTGTGATGACGTACGCGCCTTGTTGAGGAGGTACAAGGAGGGTTGGTCAATGGCACAGTGTCCCGACGCTGGAATATTCTTGTCGTGGAAAGACCAGAAGGTGGTTTGGGCAAGTGCATGGAGACCTTAG

>LcGRAS15

ATGAAGAGAGATCTCCAGGAAAGCTGCGGTGGCGGCGGCGGCGGCGGCTGCGGTGGAAGTAGTGGTAATAATAATGTGAAAGGAGAATGCTCATCAAGTAAAGGAAAGATGTGGGACGAGGGCGGCGACATGGACGATGAGCTACTGGCGGTTCTGGGTTACAAAGTCAAGTCCGCCGACATGGCTGACGTCGCGAACAAGCTCGAGCAACTGGAGATGGTGATGGTTTCTGCTCAAGAAGACGGAATCTCCCAGCTTTGCGATACCGTCCACTACAACCCCTCCGATCTCTCCGGTTGGGTCCAGTCCATGTTGTCCGAGCTCAACTGCAGCTCCACTTTCGATGAACCAAAACCAGATTTCCAACCACCACCGCATCAACAACAACAACAACAACAAATGGTCACGAGCCAGTCACGTGGTGTCTTCAACGATGATTCCGAGTACGACCTTCGCGCCATTCCCGGTGTCGCCGCCTACCCACCACAACAAGCCGATTCGGATTCCGATACCACCAGGAAAAGAATCAAAACCGGATCCGGATCCGGATCATCCCCTCTTTCGGTTTCTGAGTCAACTCGTCCTGTGATGGTGGTTGACTCACAAGCAGCAGGTGTACAACTCGTGCACACGTTAATGGCTTGTGCGGAGGCGATCCAACAGGATAACCTGAAGCTAGCTGACGCGCTGGTGAAACACATCGGCTTGCTTGCCACGTCACAAGCTGGCTCGATGCGAAAGGTCGCGACCTACTTCGCTCAAGGCCTGGCGCGCAGAATCTACAAAATCTACCCTCAGGATAGTCTGCTGGAGTCGTCCTACGACGATATCCTCCAAATGCACTTCTATGAGTCCTGTCCTTACCTCAAATTTGCACACTTCACGGCCAACCAAGCTATCCTTGAAGCCTTCGCGACGGCCACGCGCGTCCACGTCGTCGACTTCACGCTCAAACAAGGCGTGCAGTGGCCGGCTCTCATGCAGGCTCTCGCGCTCCGGCCCGGTGGCCCGCCCGCTTTTCGCCTGACCGGCATCGGCCCGCCTCAGCCCGATAACACTGACGCGCTGCAGCAGGTGGGTTTGAGGCTGGCCCACTTGGCCGACGCAATCGGGGTCGAGTTCGAGTTTCGTGGGTTTGTTGCGAAAAGCTTGGCGGATCTTGAACCCGAAATGCTGGATATCCGACCCGAGATCGAGACGGTTGCGGTCAATTCTGTTTTCGAGCTCCACCGGCTGTTGGCCCGACCCGGTGGTTTTGAAAAGGTGGTGTCTTCGATCAAAGCGATGAAGCCGAAAATCATTACGGTTGTAGAACAAGAAGCGAACCACAACGGTCCGGTTTTTCTGGACCGGTTCACGGAGGCTTTGCATTACTATTCGAGTTTGTTCGACTCACTAGAAGGGTCCGCGTTGACTCCACCGACTCAGGACGGGGTCATGTCCGAGGTGTACCTGGGGAGACAGATATGCAATGTGGTGGCTTGTGAGGGAACGGACAGGACTGAGAGACACGAGACGTTGACTCAGTGGAGGAATCGGTTGGAATCGGCCGGCTTCGATCCGGTCCACCTCGGCTCGAATGCATTCAGGCAGGCTAGTATGTTGCTGACTGTGTTTGCTGGCGGAGATGGGTATAGAGTGGAGGAGAATAACGGTTGTTTGATGCTTGGGTGGCATACGCGGCCGCTTATAGCCACCTCTGCTTGGAAACTCGCCACCACTGAGTTGTGA

>LcGRAS16

ATGTCATCCGGGTTTTCGGGTGGTGGTCCGGACTACTACGCGGCAGCTCTCGCCGGCAGATCCATGAATGTTGTTGTTAATAACGGGAACGCTTCACAGCCCTCTTATCATCATCAAAGGATGCAGCCGGGCATTTTCATGGACACCTCCTCGTCGCCTACAGCAGCGGCGGTGGCAGCTTCTCAGATCACGAACCGGGTCGGCCCGAATTGGATCGGGAAACGGACTTTCAACGATTTCCACCCACAACAACTTCAACCTCAGTTTCAACAACTACAACAACAAAATCAGGCTCCCAACAGCTTATATCTTCGGTCAGTGAAGCCGCGGACTTGTCAGAATCTCTCTCCAATCTCTCCATTACCTCCGGTGGATTTATATAACTCTTTACCAAACCGATACGGGTTCCAAAACACTCTGCTTCAACAACAACAACAACAAATACTAAAACAACAGTTACGTCCTCAACAACAACAAAACCAACCGGTCGGGTTTGTTTCTGGTTCGGTGAACCAAATAAACCCGTATTTAAACAAACAAGTTATTGAGGTTGCAGGTCAGGACTCGGAAAAGAAGATGTTGAAGGAGTTGGAGAAACAGCTTTTCGACGATAACGATGATGATGATGGAGATGCGGTTTCCGTTATAACAAACATGAACAGTGAGTGGGGCGAGACAATACAAAATCTCATGAGTCCGAGTCCGAAACCGGTTTCTCCGATATCTCCTTCTCCAACGACGTCGTCTTCTTCTTCTTCGTCGTCCGTGGCTTCTCCGGCCACGAGCTGTTCGAAACAGACGGTGATAGAACTGGCGACGGCGGTTTCCGAGGCGAGAAACGACGTCGCGGCAGAGATCTTAAATCGGCTGAGTCAGGCGTCGAGTTCGAGAGGGAGTTCGGAGCAGAGGTTGATGGAGTATATGAGTTCGGCGTTGAAATCGAGGATGAATATGGTGGAGAGCCCGGCGCCAGTGGGCGAGTTATTCACTCAGCAACACGCTGAGTCGACTCAGTCTCTGTATGATTTGTCTCCATGTTTCAAGCTCGGTTTCATGGCTGCCAATCTGGCAATTCTTGATGCTACTAGTGATTGCAACAATTTTCACGTAGTGGATTTTGATATAGGACAAGGTGGACAGTACATGAATCTCCTCTATGCGCTGGGGAAGAGGCCGAACGGGAAGCCATCCGCCGTCAGCATCACGGCGGTGGCCGACAATGGCGGAGGAGAAGAGAGGTTGAGCTTCGTAGCTGATCTGTTGACAGAAGTCGCCGAGAGAGTTGGTGTTTGTTTGAATTTCAAAGTAGTGACTTCATTAAAACTCGGCGATCTGAGTCGTGCCTCATTGGGTTGTGACGCGGACGAGCCACTCGTTGTGAATTTCGCGTACAAGTTGTTTAGAATGCCTGACGAGAGCGTTTCGACGGAGAATCCGCGTGACGATCTCCTCCGGCGCGTGAAGGCGTTGTCGCCGCGCGTGGTGACATTAGTGGAGCAAGAGATGAATACGAATACGGCGCCGTTTTTGCACCGCGTGAGCGAGGCATGGGGTTACTATGGCGCGTTGCTTGAATCGATCGAGTCCGCGTCGAAGGACCATCAGGACCGAGTCAAGGTTGAGGAGGGACTGGGTCGTAAACTCGCTAACTCAGTTGCATGCGAAGGCAGAGACCGAGTCGAGAGATGCGAGGTGTTTGGGAAGTGGAGGGCCCGCATGAGCATGGCTGGTTTCCAGTTAAAACCAATGAGTCAGACAATCGCCGAGTCCATGCGGGCCAGACTCAATCCGCTTAATCGGGTCAACCCGGGTTTCACCGTTAAAGAAGAAAACGGAGGCGTGTGTTTCGGCTGGTTGGGTCGGACTCTCACCGTCGCGTCTGCTTGGCGTTAA

>LcGRAS18

ATGGCCATCGAGTTTGATGACGCCTTTGAGCTCGACTTCTCTAGTTATAGCTCCACCACCACAACAACTACAACCACCACAGACGATGACCATTGTTGTAACTGGAACGACTGGTCCCCAGTTATCGACTGGGAAGCTTTATCCGGAGGCCAGGATGATTTTCACGACCTCATCGATTCGATGATTGACGAAGGTGGATTATTAAACCCTTGTCGAGTTGCCAATGAATCGTCCAACTCCGTATCAACTGACACCATGACACTAGACGAAGAAACCAACGGCGACGATTTCAAAGGCTTGAGGCTGGTTCATCTTTTGATGGCGGCAGCTGAAGCACTAACCGGAGTGAATAAGAGCCGTGAACTGGCTCGGGTGGTATTGGTTCGGCTCAAGGAGTTGGTTTCTCATAATGACGGAACCAACATGGAAAGGTTAGCAGCTTATTTCACCGACGCCTTGCACGGTTTACTAGAAGGCGCCGGCAATAAGCATTTGATATGTAATGGACCTTATCACCATCACCACCGTGACGAGAATCATCAGACGGACGTGCTAACTGCGTTTCAGTTGTTGCAGGACATGTCCCCATATGTTAAGTTCGGACACTTCACAGCCAATCAAGCGATTTTTGAAGCTGTTGTTCATGATAAGAGAATTCACATAGTGGACTATGATATCATGGAAGGAATCCAATGGGCGTCGTTGATGCAGGCCTTGGTTTCTAGAAAGGATGGCCCTCCAACCCCACATCTTAGGATCACAGCCTTATCAAGGGGCGGTAGTGGTCGAAGATCATTGGCACCGTCCAAGAGACGGGTCGACGTTTGGTGGCATTTGCTGCATCAATCGGCCAACCATTCTCTTTTCATCAGTGTAGAGGAAGTTGGACCCATTGAGGATGGAGGTTTTGTGGGACGTTTCATGGATTCATTGCACCATTACTCGGCAGTTTACGACTCACTCGAGGCGGGGTTTCCAATGCAAAGTCAAGCAAGGGCCTTAGTAGAAAGAGTGTTTTTGGGGCCTCGAATATCCGGATCCATAACCGGCATATATCGAACCCAGGGTGAGCAAAAGGGTTGCTCTTGGGGCGATTGGTTGGGCGAAGCGGGGTTTAAGCGAGTTAACATAAGCTTTGCCAATCATTGTCAAGCCAAACTATTATTAGGCTTATTTAATGATGGGTATAGAGTGGAGGAGCTGGCCAATAATAGGCTGGTTTTGGGCTGGAAATCTAGGCGTTTGCTTTCTGCTTCTGTTTGGACTTCTTAG

>LcGRAS19

ATGGCCATGGAGTTCGATGACGCCTTTGAGCTCGACTTCTCTAGCTATAGCTCCACCACAACAACAACAACCACCACCACCACCACGGACGATGATCTTGGTTGTAACTGGAACGATTGGTCTCCAGTTGTTGACTGGGAAGCTTTATCTGGCGGCCAGGATAATTTTCATGACCTCATCGAATCCATGATGGATGAAGGTGGCTTAAACCCTGCTGGAGTCGCCAATGAAACGTCCAACTCTGTATCAACTGACAGCACGACAGTGGATGAAGAAACCAACGGTGAGGATTTCAAAGGCTTGAGGCTGGTTCATCTTTTGATGGCGGCAGCTGAAGCACTAACCGGAGTGAATAAGAGCCGTGAACTGGCTCGGGTGGTATTGGTTCGGCTCAAGGAGTTGGTTTCACCTAGTGATGGAAGCAACATGGAAAGGTTAGCAGCTTTTTTCACCGATGCCCTGCAAGGTTTACTTGAAGGTGCCGGGAGCAAGCATTTGATATGTAATGGACCTCATCACCATCACCAGCGTGACGAAAATCATCAGACGGACGTCCTATCTGCGTTCCAGTTGTTGCAGGACATGTCCCCATATGTCAAGTTCGGACACTTCACAGCTAATCAAGCTATTTTAGAAGCTGTTATTCATGATAGGAGAGTTCACATAGTGGACTATGATATCATGGAAGGTATCCAATGGGCATCGTTGATGCAAGCCTTGGTTTCTAGAAAGGATGGCCCTCCAACCCCACATCTTAGGATCACAGCCTTATCAAGGGGCGGTAGTGGTCGAAGGTCAATTGGGACCGTTCAAGAGACGGGTCGGCGTTTAGTGGCATTTGCTGCATCAATCGGTCAGCCATTCTCTTTTCATCAGTGTAGGTTGGACTCTGATGAGACATTTAGGCCATCCTCATTGAAGTTAGTTAGAGGGGAGGCATTGATTATCAATTGCATGTTGCATCTCCCTCACTTTAGTTATCGGGCACCTGATTCTATTACTTCTTTTCTATTCGGATCCAAAAGCCTAAACCCGAGGTTGGTAACCCTGGTGGAAGAGGAAGTTGGACCCGTAATTGAGGATGGAGGTTTTGTGGGACGTTTCATGGATTCATTGCTCCATTATTCAGCAGTTTACGATTCACTAGAGGCTGGTTTTCCAATGCAGAGGCGAGCAAGGGCCTTAGTAGAACGAGTGTTTTTGGGGCCCCAAATATCCGGATCATTGGCCCGGATATACCGAACCCAGGGTGAGGAAAAGGGTTGCTCTTGGGGCGAGTGGTTGGGTGGTGTTGTGGGGTTTAAACCAGTTAACATAAGCTTTGCCAATCATTGTCAAGCAAAGCTGTTACTAGGTTTATTTAATGATGGGTATAAGGTGGAGGAGTTAGCCAATAATAGGCTAGTTTTGGGATGGAAATGTAGGCGTTTGCTTTCTGCTTCTATTTGGACTTCTGCAGATTCTGATTTGTAA

>LcGRAS20

ATGTCATTAGTTAGGTCTGCAGAGCCATCTACTACATCGTATGGAAATTCTAAGCTTTATTCGTTAAAGGGGAGGGGTGACAATTCTGCCTTGTCTACTCAAGTATTCAGCTCTGACAAACATAAGATGATGTACATGACTGATTCTTACAGCAGTGAGAGCTACGAGAAGTACTTCCTTGACTCTCCAACAGAAGAACTCGTGCATCCTTCAAGTTCTACTGTCTCAGACAATTACTTTCCCCCGGAAGATGCCTCTTCTTACCAGCTAAGAGTTGGTTCAAACTCCACCATGGCTGTGCAAAACCCACTGAATACTTCTTTCATGTCCATGAGACTTCGTGATAGCTATCAATCCAACTTTGAGTGTGATTATCTGGAAAGTCCAAGCCTAGATCATTTAGACTATGATGAAATAACGATGAGATTGAAGCTTCAAGAACTTGAGAGAGCATTGCTTGATGATAATGATGGTGATGATGACAATGACATGTTTGCTACTGGTCAAAACATGGAAGTTGATGGTGAATGGTCTGACCCGGTCCAGAATGTGCCATTCCACGACTCACCGAAGGAGTCTTCATCCTCAGATTCTAACCTTAGCAGCATCAGCTGCAACAAAGAAGTATCGCAGATATCTCCTCGGAGTCCTAAGCAAATGCTTATAGATTGTGCTGCTGTGCTTTCAGAAGGAAACATTGAGGAAGCAACAGCTATAATAAATGAACTCCGGCAGATGGTCTCAATTCAAGGAGATCCTCCTCAAAGGATTGCAGCCTACATGGTGGAAGGGCTTGCAGCTCGCATGGCTGCTTCTGGAAAGTTTCTCTATAAAGCTCTGAAATGCAAAGAGCCTCCTTCTTCTGATAGGCTTGCAGCGATGCAGATCCTCTTTGAGGTGTGCCCTTGTTTTAAATTTGGATTTATGGCAGCAAATGGTGCAATTATTGAGGCATTTAAAGATGAAAAGAGAGTGCATATAATAGATTTTGACATAAATCAAGGTAGTCAATACATAACATTGATACAAACAATTTCTTCACAGTCTGGTAAGCCACCCCATTTAAGGTTAACTGGGGTTGATGACCCTGAGTCAGTTCAACGGCCTGTTGGAGGCCTTCAAATTATTGGATTAAGACTTGAGAAGCTAGCCGAAGCACTGGGGGTACCGTTTGAGTTTCATATAGTGCCTTCAAAGACTTCAATTGTCACTCCATCAATGCTCGACTGCAGGCCTGGGGAAGCACTTATTGTAAACTTTGCTTTTCAGCTTCATCATATGCCTGATGAGAGTGTTTCAACTGTAAATCAGCGAGACCAGCTGCTTCGGATGATTAAGAGCTTAAATCCGAAACTTGTAACTGTTGTTGAACAAGATGTGAACACAAACACTGCCCCCTTTTTTCCAAGGTTTATTGAAGCCTACAATTACTACTCTGCTGTGTTTGAGTCTCTTGATGCAACTCTCCCAAGGGAGAGTCAGGACAGGATGAATGTTGAACGACAATGCCTGGCACGGGATATAGTTAACATTGTTGCATGTGAGGGAGAGGAGCGAATAGAGCGGTATGAGGTTGCAGGGAAATGGAGGGCAAGGATGAAGATGGCCGGCTTCACTTCGTGTCCAGTGAGTCAGAATGTGATTGATATGATTCAGAAACTTATAAAGCAATACTGTGACAGGTACAAGATGAGGGAGGAAATGGATCTGCTGCATTTTGGGTGGGAAGACAAAAGCTTGATCGTTGCTTCAGCCTGGAGGTAA

>LcGRAS21

ATGGAGGATACAGATGAGGAGGAGTTTCTGAATCTTAGCTTGGCCATCGTTGCGGATTCGTGTAGGCCAAAGAGGAAGAGATGGGCAAGTACTTTGATTAACCCTAATTTGAACCATGCATGTCATGAAGGTTTTGAAGGGAAGATTTTTAGGCTTCTTCAAATGAGAGATCAAATGTTGAAGTTGGAAACCAAGAGAAAAGGAGTGGTTGAAGAAGGAAAAGGCCTTCATTTGATCCATCTACTGCTCATAACCGCCACCGCGGTGGACGAAAACAACCTTGATGTAGCCTTGGAGAACCTCACCGAGCTCTACCAAACAGTGTCCCTTACCGGTGACTCTGTCCAGAGAGTTGTGGCCTATTTCGCCGACGGTCTTGCCGCCAGACTCCTGACCAAAAAATCTCCCTTCTATGGCACGATCATGAAAGAACCAACAATTGAAGAAGAGTTTTCAGCCTTCACGGATCTCTACAGGGTCTCTCCTTACTTCCAATTCGCTCATTTCACTGCCAACCAAGTCATCATCGAGACTTTCGAAAAGGAAGAGGAAAATAACAACCGCGCGTTGCACGTGGTCGATTTCGACGTCTCCTATGGCTTCCAATGGCCTTCTCTCATCCAATCTTTGTCTGAAAAAGCTACAATCAGCAACCGGATTTCGCTTCGAATCACCGGATTCGGAAGAAGCATGGAAGAGCTACAAGAAACTGAGAGCAGATTAACTAGTTTCGCAAAAGGGTTTCGCAATCTAGTCTTCGAATTCCAGGGACTATTAAGAGGTTCCAAGCTGACCAATCTCAGGAAGAAGAAGAACGAAACGGTTGCAGTCAACTTAGTCTTCCACCTAAACACTTTGAATAATTTTTGGAAAATTTCTGACACGTTAAAATCGGTCCATTCTCTCAACCCCTCCATCGTAGTCCTTGTAGAACAAGAAGGCACCAAGAACCCTAGAAACTTCCTCGCAAGATTCATGGAGTCTCTCCACTACTTTGCCGCCATGTTTGATTCCCTAGACGATTGTCTGCCATCGGAGAGCACAGAGAGGCTCGGCATAGAGAGGAACCAGCTGGGTAAAGAGATCAAAACCATGCTCAACTATGACAAAGGAGAAACAAACCATCCAAGATATGAGAAGATGGAGACTTGGAAGACAAGAATGGAGAGCCATGGATTTGAAGGGATCAAAATAAGCTCCAAGTCCTTGATTCAAGCAAAACTTTTGCTCAAAATCAGGACCACCCATTATAGTCCTCTTCAATTCGATGGAGATCAACATAGTGGGTTTAAACTGTTTGAAAGAGATGATGGGAGAGCTATCTCACTTGGGTGGCAAGATAGGTGTTTGCTTAGTGTCTCTGCATGGCTTTGTGTATGA

>LcGRAS3

ATGGTACTGTACGAGTCGTCCACGCCAGCCGCTACTGGAAGCAGCAGTAGTAGGGGTAGCTCATCCTCCTCCGCTACCAAGCCACCTGCCGACATCGACGGCTTGCTGGCGGGTGCAGGCTACAGGGTCAGATCCTCTGAGCTACGTCAGGTGGCTCACCGACTTGAACGCCTCGAAACCGTCATGGTTAACGCTCCTGCCGATGTCTCTCTAATCGCCTCCGACACCGTCCACTACAATCCCTCCGATCTCGCCTCCTGGGTCGACTCTCTCTTGTCTGAATTCAACCAGCCGCCCCTCCCTCTACCGTCCGATCTACCAGACTTTCCGGATGGAAATCCCACGGTTCTTAATTACAACTATTTGACGGGCGAAAGGGTTAGTCAGCAGCAATTGACGGTTGTTACTGCCATGGAAGAGGATTCCGGTATAAGGTTGGTCCATATGCTGATGACGTGTGCTGAGTTGGTGCAACGTGGCGAGCTTCCACTGGCCAGTTCGATTGTTGAAGATATGAAAGGCTTGTTAACTCATGTCAATACTGGTTGCGGCATCGGCAAAGTCGCCGGTTACTTCATCGACGCGCTTAGCCGCCGGATTCTCGGAGTTGGCGGCTCCATCTGCGGCTCAGCTTACGAAAACGAGATTTTGTATCATCACTTCTACGAGGCTTGCCCTTATTTGAAATTCGCTCACTTTACAGCTAATCAGGCTATCTTAGAAGCATTCGACGGTCACGATTGTGTCCACGTTGTCGATTTCAATTTGATGCATGGACTGCAATGGCCGGCTTTGATACAGGCGCTGGCCCTGCGTCCAGGTGGCCCTCCTCTGCTACGGTTGACCGGCATTGGGCCACCATCACCGGACGGACGCGACTCGCTCCGCGAAATCGGGTTAAGACTGGCGGAGTTGGCTCGGTCTGTGAATATAAGGTTCGCTTTTCGTGGAGTGGCGGCTTCGCGTTTGGAGGATGTGAAGCCGTGGATGCTTCAGGTTAGCCCCAAAGAAGCGTTGGCGGTCAACTCAATCATGCAGCTGCACAAATTACTGGGTTCCGACTCAAACCGAAATTCAGCGATGGAGATGGTTTTGGGTTGGATCCGGAACTTGAACCCGAAGGTCATGACAGTAGCGGAGCAAGAAACGAACCATAACCAACCGGAATTCTTGGACCGGTTCACAACTGCCTTGCACTACTACTCAACCATGTTCGACTCACTGGAGGGCTGTTCGCTTCAGCCGGAGAAGGCTTTAGCCGAGATTTACATACAGAGAGAAATAGTGAACGTAGTCTGTTGTGAAGGGTCGGCGCGTGTAGAGAGGCACGAGCCACTGGCTAAATGGAGAGCCAGGCTTGGAGCAGCTGGCTTCAGGTCATTGCCTCTGGGTTCGAATGCTTTTAGGCAAGCCAGTATGCTGTTGACTCTCTTCTCAGCTGAGGGTTACTCTGTTGAGGAAAATGACGGTTGTTTGACTCTCGGCTGGCATAGCCGTCCTCTAATTGCGGCTTCGGCTTGGCAAGCCCTACCGGACCATGCCATCAATCATCACAGTAACAATAATATTTTGTAA

>LcGRAS40

ATGCAATCCCCAGAAGCCCAAGAGCCCCTACCATCTCCTACTCTCTCTTCTGATGAAGGTGATGTTTCCTCATCCACTATGACCGAACTGTCATCAAGTTGGATGAGGGAGCAGGAACTGGTTGACTTATTTGTTGATAACTTACCTCATGATGACTCATCTGTTGATCAAGACGATGACTTGATGAGGTTCAGACTGAGAGAATTGGAGAAAAATTTATTCGGGTCTGAATCAGACATCGATGATAGTGGCTTCAGGGGTGGGGCCAGCCAAGACACTTCAGCTGCCGGGCGGAACTGGAATCAACTGATGGAAATGAGCCCGAGGTTAGACTTGAAACAGGTGCTTGTTGGTTGTGCTCAAGCGATATCTGAGGGCGATATGTCAACAACGGAAGGTATGATGCATGTGTTAGAGAACATGGTTTCAGTCTCTGGTGAACCGATTCAAAGGTTGGGCGCGTACATGTTGGATGGGCTTAGAGCAAGATTGGCTTTTTCAGGGTATAATATCTACAAAGCCTTAAACTGCGATGAACCAGTAGCAGTAAGCTCAGACCTGATGTCTTACACAGGTATCCTTCATCACTTCTGCCCATATTGGAAGTTTGCATACATGTCTGCAAACTTCGTCATAGCAGAAGCTGTTTGGAGTGAACCTAGAATTCACATAATTGATTTTCGGATTGTACAAGGCAGCCAGTGGGTCACACTTATCAAGCTTCTAGCTAAACAGCCTGGTGGACCTCCAGCTATTCGCATAACTGGAATTGATGGTACCCAATCATACTTTGCTCGAGGTGGGGGACTCGATCTTGTAGGGCAGAAGCTGTCAAAGGTTGCTGCGTCATTCAATGTTCCATTCGAATTCCACAATGCTGCTATGTCTGGTTGTGAGGTTGAACGAGAACACCTTAGCATCCAGCCTGGGGAAGCCGTGGTTGTGAATTTTCCTTACGTGCTGCATTGTTTGCCGGATGAAAGTGTAACCACTGCAAATCACAGAGACCGCCTATTGAGACTCGTGAACAGTTTATCACCGAAAGTTGTCACCATTGGTGAGCAAGAATCCAATACCAATACCATCCCATTCTTCCAGAGGTTCCTAGAGACACTAGACTATTATACAGCTATGTTCGAATCAATAGATGCAGCTTCTCCAAGGGAGGACAAGACAAGAATCAATGCAGAACAGCACTGTGTGGCTCGCGACATTATCAGCATGATCGCTTGTGAGGGCCCTGAGAGGGTAGAACGACATGAACTTCTAGCAAAATGGAGATCACGATTAAAAATGGCTGGATTTACCCCGTATCCTTTAGGCCCTTCTGTTATAAAAATTGCTCAGGGTCTATTAAAGAACTATAATGAGAATTACAGAGTCCAAGAGCATAATGGTGCTCTCTATCTTAGCTGGAAGAACAGATGTATGGCAACCTTTTCTGCTTGGAGGTGA

>LcGRAS26

ATGGCATATATGTGCGCCGACAGCGGCAATTTGATGGCCGTTGCAGAACAAGTCATTAAACAAAAGCAAGAACAAGAACAGCAACAGCAACAACACCACGTTAATCAACAGCAGCAGCTCTTCTCTCTCAATCCTTTCTCTCTAAACCCTTGTTGGGCCGCGACTTCTACCACTATCACCACCCACATGTCTAATAGTCCCAACTTCGGGTTCGGGCTCACGGGCTCGGGCTTCCCCGACCCGTTAGATGCCGGAGAGAACGAGTTCCAGTTCCCGCAAATTGACCACCATTCGACTGGGTTTAGGTTCTCAGATTTTGGAGGTGGAGGTGAGTTTGACTCGGACGATTGGATGGATAGTTTGATGAACACCGGACACTCAACGGATAGTTCCACTCTGCCTTCTGCTTGTGACGCGTGGCAAAATAATGCTGATTTCGGTCTGTATACTACCGATCCTTTTGCCACCTGCCCGAGTCGACTCAACGTTGGTTGTTGCTCTTCTCCGTCCGATCTCAACCGTGTTGTCTTCATTGAACCTCCAAAGAGCCCCGTCCAAGCCTGGCCGCCGTCCCCCCCACCTCCGCCGCCGCCTCCACAACCGGTTGTGAAGGACACCAAACCAGCCAATGTTTCGCAGCCAAGTGTCCACCAAGCCTCTACTAGCTCCTCCCCGGAAAATGACCTCTCACCGCCACTTTTAAAAGCTCTACTGGACTGTGCTCGACTCGCCGAGGCAGAACCCAGTCGAGCTCTCAAATCGCTGATTAAACTCAGAGACTCGGTCTCCGAACACGGAGATCCAATCGAGAGAGTCGCGTTTTACTTCATCGAAGCTCTCTACAACAGAGTCTCTCTTCAAGAAGACAAAACACTCTCCGACTTCACTGTAAGTTCTGCTGATTGCATTATCTCGTACAATGCATTGAACGACGCCTGCCCATATTCGAAGTTCACTCACTTAACGGCCAATCAAGCAATATTTGAAGCCACCGATAGTGCAACCAAGATTCACATAGTCGATTTCGGGATTGTTCAAGGAGTCCAATGGGCCGCTTTGTTACAATCTTTCGCGACCAGGTCCGGCGGAAAACCCATCAAAGTTCGGATCTCGGGTGTACCCGCTCCGTCACTAGGTGACTCACCAGCTTCTTCACTTCTCGCTACTGGTATCCGACTCAGCGAATTCGCTAGACTCTTCAACTTGGACTTCGAGTTCCAGCCGATTCTAACACCCATCAACGAACTCAACGTGTCGAGTTTCAAGGTCGAGTCAGACGAGGTGGTTGCAGTCAACTTCATGCTCCAGCTAAACAATCTACTGGACGACACACCAAACGCTATAGAGAGCGCTCTCGCAATGACGAAGTCATTGAACCCAGTTATCGTCACGCTTGGTGAGTACGAATCGAGTTTGAATCGGGTCGGGTTCGTCGCCAGATTCAAGAACGCATTGAAGTACTACATGGCCGTGTTCGAGTCACTTGAGCCCAACATGAGTCGAGACTCAGTAGAGAGACTCCAAATAGAGAAACTGCTTCTGGGTCGAAGAATCGCTAATGTGATAGGAACCGAATCCACCCATAGAAGAAGAGAGCGGTTGGAGGATGGAGAGCATTGGAGTTGTTTGATGAAAAATTGCGGGTTCGAACCGGTACCACTCAGTCATTTTGCTATTAGTCAAGCGGAGATACTACTATGGCACTGTAATTACAGTTCTTCATACTCGTTAATTCATGCTGACACTGAACCTGAATACATAACTCTAGCTTGGAACAAGGTGCCTCTACTTACAGTTTCATGGTGGCGTTAA

>LcGRAS28

ATGATGCAATTCACCGAAACCCCACCACAACCCTTGCATCAAATCGCTTCATTTTCAAACCTCACCATGAACAAGAACCAACTTCAGAGGACTCGTCCATGGCCTGGTTTCCCCACATCAAAGTCCTTGGGCAATTTCGGTGACGCCAATTGCATGGAACAACTTCTGGTTCACTGTGCAAATGCAATCGAAAGCAACGACGCCACTCTCGCCCAGCAAATCTTATGGGTTCTAAACAACATTGCACCACCAGACGGCGACTCAAATCAGCGCCTCACATGCGCCTTCCTTCGCGCTCTGATTGCTCGTGCTGCCAAAAGTGGCACCTGCAAAATGCTCGCTGCAATGGCCAATGCTCACTGCAACCTCGCTATAGACAACCACAAGTTCTCCGTTATCGAGCTCGCTAGCTATGTGGACTTGACTCCTTGGCATCGGTTCGGTTTCACTGCCGCAAATGCAGCCATGCTAGATGCTGTTGAAGGCTACTCAGTCATCCATATTGTTGATTTGAGCTTGACACATTGCATGCAAATTCCTACACTCATCGATGCTATCTCTAATCGGTTCGAGGGACCGCCGAGACTAAAGCTCACAGTAGCTGGCGCCATTGAAGACATACCACCTATGCTTGATCTTTCATACGAGGAATTGGGGTCAAAGTTGGTCAACTTTGCTAGGTCAAGAAATATTATATTGGAATACACGGTAGTGCCTTCAAGTTACGCTGATGGGTTTGCTTCTTTGATCGAACAAATCAGGGTGCAACATCTAGTCTATCCAGAAAGCGGTGAGGCTTTAGTTATTAACTGTCACATGATGCTTCATCATATACCAGAAGAAACCATATCAGCAATTCCAAATGCAATTTCAAATCCTTATTGGCTAGACTCTTCCCCCACTTCCTCTCTTCGAACGTTGTTTCTCAAGTCTCTAAGGAGTTTGGAACCGACTATTGTTATATTAGTAGATGAAGATGCGGATTTGACATCAAACAATTTGGTTTGTAGATTAAGATCTGCTTTTAATTACTTATGGATTCCTTACGATACAGTCGATACATTTCTTCCCCGAGGAAGCAAGCAAAGGCAGTGGTACGAGGCTGACATTTGTTGGAAAATTGAGAATGTTATAGCACATGAAGGTCTCCAAAGAGTGGAGAGGCTTGAGCTGAAAAGCCGGTGGGTGCAGAGAATGAGGAATGCAAACTTCAAGAGTATAAATTTCTGTGAAGAAGCGGTTTCGGAAGTGAAAACCATGCTTGATGAACATGCAGCTGGTTGGGGATTGAAGAAGGAAGAAGATGATCTTGTACTTACATGGAAAGGCCACAATGCAGTGTTTGCTTCTGCTTGGTTGCCTACTTGA

>LcGRAS5

ATGTTGCGATCTTTGAATACCACATCTGGTCATGAGCAAGACGAAGAAGAAACCTCGTCGGCCACAGATCTACAGTACCAGCAGATTACACAGCCATCACATTCGAGGGGATTGGACTCACCAGCAGCAGCAATCCAGATGCGGCAGTTACTCATAAGTTGTGCAGAGCTGGTGTCTCAGTCAGATTTCTCAGCTGCCGACCGCCTCATCTCCATTTTGTCAGCCAACTCTTCCCCATATGGTGATTCCACAGAAAGGTTGATCCACCAATTCGTAAGAGCCCTCTCTCTCCGTCTGAATCGCCACCATCATCTTCATGCTTCGGCTACTGGCTTCATGATGAATTTGATTACTACAAACATCGCTACTACTAGTATTAACACCAACAGTAGTAGTACTACTCCAAGCATAAGTACTAGTGCCAACTATATCATCAGCAATGACTCAAGTGCTCTTCAAACATGCTATCTATCTCTAAACCAAATAACGCCCTTTATTAGGTTCAGCCATCTCACAGCCAATCAGGCCATTCTTGAAGCCATACAAGTAGGGCAACAATCCATCCACATCCTTGACTTCGACATCATGCATGGTGTTCAGTGGCCTCCTCTTATGCAAGCATTAGCTGAACGGTCCAACAACACTCTCCACCCTCCTCCTATGCTTCGAATCACTGGAACTGGCCACGATTTGGACATCCTCCACCGCACTGGTGATCGCCTCTTCATGTTTGCTCAATCTCTCGGCCTCAGATTCCAATTCCACCCTCTCCTTCTCCTCAACGATGACCCCACTTCAGTTGCCCTCTACCTCTCTTCGGCCCTCTCTCTCCTGCCAGATGAGGCCTTGGCTGTCAATTGCGTCTCGTATTTACACAGACTCGTGAAGGAAGACAGCCGGGATCTGCGCCTCTTCCTCCACAAAATCAAGTCCTTGAACCCCACAGTGGTGACGATTGCGGAAAGAGAAGCCAACCACAACAACCCCGTCTTCATGAGGAGATTCGTGGAGGCATTGGACCACTACTCGGCCATATATGAGTCGCTAGAGGCAACTTTGCCACCAAACAGCAAGGAGAGACTAGCAGTAGAGCAACTATGGTTTGGAAGAGAAATAACGGACATAGTGGGGGCAGAGGGGGAGAATAGAAGAGAAAGGCACGAGAGGTTGGAATCGTGGGAGGTGATGCTTAGAAGTTCAGGCTTTGCAAATGTCCCTTTAAGTCCTTTTGCTTTGTCTCAGGCCAAGCTCCTTTTAAGACTTCATTATCCCTCTGAAGGTTACCAGATTCAGATTCTTAACAATTCTTTCTTCCTAGGTTGGCAGAATCGTGCCCTTTTCTCAGTCTCTTCCTGGCACTAG

>LcGRAS6

ATGCAAGAATCTGAGCAACCTAAAATTACAGATGAAAGTCGAACAGTCTATAACCAGCCCATGCAACAGCTGGTCGCTTATAGCTGGCCTCCCATTCAGATTGTGGAGAACAACTTATCCTCTGATGATAGCAGTGAAGGAGCGCACCTTTCAGTAGAGCTTTTTGAACAGCACTGCACC

CTTGAATCATCCTCCGGAACGGATGGTTACCATGCTAATATTTCTCCGTCTACTGCCAATTTCATGCCAAAGAAAAGCCCCTTTATGTGGCCGAACTCTCAGTCCTACCCGTCGGAACTGCATCATTCTCCTGACAATACTTGTGGCTCACCAGTCAGCGGTTCTTGTGTAACAGACAAC

AGGAATGACTTGAGGTATAAGATTAGAGAATTGGAAAATGCTATCCTAGGTCCTGAAACAGATGACTTTGATGTATGCAATATCATTCTCCCTGGTGAAGAGCAAATTTCATCGGAGTTAGAGCAGTCGAAATACGTGACGGAGATAATCTCTAGACGGGATTTGAAAGAGATGCTTTGC

ACTTGTGCAAAGGCGGTAGAGAATAATGATATGGTCACAGTCGACTTGTTGATGAAAGTATTACAACAGATGGTGTCGGTTTCTGGTGAGCCTATCCAACGTTTGGGGGCTTACATGTTGGAAGGGCTCATTGCACGGTTGGCCGCTTCAGGAAGTTATATCTACAAAACTTTGAGATGC

AAAGAGCCTGTTGGTACTGACTTATTCTCGTACATGCACTTACTATATGAAGTCTGCCCATATTTCAAGTTTGGATACATGTCAGCAAACGGAGCAATTGCTGAAGCCATGAAAGATGAAAGTAGAGTCCACATAATTGATTTTCTAATTGCGCAAGGTAGTCAGTGGATGATTCTTATT

ATGGCTCTTGCTTCTCGGCCTGGAGGCCCCCCGCATCTTCGAATCACTGGCATTGATGATTCCATATCAGCTTATGCCCGAGGCGGAGGACTTGAAATTGTAGGGAAAAGACTAGCGAGGCTTGCTGAGTCATATAAGGTACCCTTCGAGTTTCATGCCACTGCTATGTGTGGCTCCGAG

GTTCAACTTGAAAACCTTGAAATTCGCCTGGTTGTAACTCTTGTCGAGCAAGAAGCAAATACCAATACAGCCCCTTTCTTTCACCGTTTCCTTGAGACAATGAATCATTACATGGCCATCTTTGATTCCGTTGAGGCTGCTCTGCCAAGAGAGCACAAAGATAGAATCAACGTTGAGCAG

CACTGTCTGGCTCGGGAGATTGTCAACATCATTGCATGTGAAGGACCTGAAAGAGTGGAACGTCATGAGCTTCTGGGCAAGTGGAGATCACGTTTTACAATGGCTGGCTTTACACCATATCCATTAAGCCCTTTCGTGAATGCAACCATCAAAACTTTGCTGGGATCTTACAGCAACAAT

TACACTCTTATAGAGAGGGATGGAGTACTCTTGCTTGGGTGGAAAAATCAAGTTATAGTCGTTTCTTGCGCATGGAGGTGA

>LcGRAS43

ATGGAGGGTTTTGAGATAAATGATCACAATCATGAATCCTTCTCTCCTCCTTTTAGCACAACTGATGAGAACAATTCCCTTGAAACTTCACCCACAGAAGTTGTTAATAATTTTTATGCCAATTCTCATGAAAACATCTACTATGAGAACCATCATGACATTTTTTCTTTTCATCATGTCAATGAAATCGTTTCCCAACTCACTACTTCTCCTCGTGAAAATGTTGTCAAATCTTCTTCTCTCGATAGTTTGGAGGACTTGAATAGTTTCGAAATGGAAGAGGTGAGAGATTGGTTGGCGGTTGATGACGATGATCATGAGGAAAACACATGTACTTCTTCTCATAATCATGATCAAGTTGTATTGTCTAATGTTGTCTCCTCCGTGAATGTTGCATCAATCAAGTCATCTCTTATTTCACCAACAGAAGATATGGAACTCGACCACCAACTTGGCTCGAACCATCTAGTCAAAGCTTACACTGAAGCCATCGAGAACCAAAATAGAGAGCTTGTGGAGGTGATCGTGAGACGCATTAATGAGAAGGTCAGTCCCGTTGGTGAGGCTCGAGAGAGGCTTTTCTATCACTCATTTCATGCCGTGGACAAGCAAGCCGATGGCTACATGAAGCAAGAGTCAAGCAAGGTCTTCCAATCAGCTTTCGAAGCCATTTATCGAATCGTCATAAACGGAATGTTCGCTCATTTTGGAGCTAATTCCACGATTTTAGAAGCCATGCCCCGTGACATTGAGCTGCTTCACATAGTTGATTTTGACATTGGAGAAGGAATTCAATGGTCTTCTTTGATTGTTGCTCTTGCATACCAACTACAACAACAACAACAACAACATAGTTTTGGTTTTGTTGCTCAACAAATGTCCTTGAAAATAACATCAATCAAGTGGAAGGAGGAGACTTCTACTCATCTACTTCCACAATGGCCATTTGAAGAAACAAAAAGGAGGCTCCAAGACTATGCAAATTTTCATGGTTTGAAGCTAGAAGTGGAAGAGATTGAAATGGAAGATTTGGTGAAAGAAATGAAGATAAAGAAGAAAATAAGAGAATGGTTGGCTTTCAACTGCATGTGGGCTCTTCCTCACATGGGAAGAAGAAGAAGTAGAAGGAGAGCCACGGAATTTCTAGATGTCGCGACCACCCTTTTGGCAGATTTTGAGAGTTATGGTGTTATCACTCACGGAGAAGGAGAGAGTTTAGAGAGAATCATGAACAACTGCTCTTGTTTCGGGTCATTTTTCGAGAGTTACATGAAGCATTACCAAACATTGTTAGAGTCAATGGAATGGAGCTTCCCGTCACGGCTTGCGGAGGCAAGAGTAGCTATGGAGAGTCTGTTTGTGGCGCCATATGTGTCCTCTCAAGCTCTGCATGATATGTGGAGGGAAATGAGGGAACTCGAATCGGTTTGTCGTGGTTTTTGTCAACCATTGCTTGGGTTTGGCTTGAAGGTGAGCTCTGAGAGGTTGATGGAGGCCAAAGTGATGGTGAGAGAAGGAGAGAGTTTGTATAGTGTGATGGTTGAAGAAGAAAACAACAATTCAAATGAAATGGTTTTGGAATGGAGAGGGACTCCACTGGTGAGGGTTTTTGCATGGAGATTATGA

>LcGRAS44

ATGACTATGGATCCCAGGTTGAGGGGGTTTTCTGGTTCGCTTAATGGGGTCCAATTGAGTAATAAGCCGAATTCAGTCTTTCCAAATCAGAACATTGTTGCTGGAAGAATCGATAATGTCTTTCTAGATCACAATTTTAGGGACGATCGTTACCTTCAGTCTGATCCAACTCCCAGTAGTGTTGCCTCATGTCCAAGTGTGAACCATGAGGAAGATTCTCCAGAAGACTGTGATTTCTCGGATGCAGTTTTGAGATACATAAACCAGATGCTTATGGAAGAAGATATAGAGGATAAGACTTTCATGCTTCAGGAGTCGTTGGACCTTCAAGCCGCTGAGAAGTCATTTTATGATGTTCTTGGCAAGAAGTATCCACCTTCCCCTGAACCGAATTTGAGTTATACTGATCAAAATGGTGGGTACCTAGATGGTAATACCTCTAGAAATTCTGGAAACTGCAACACTAGTGGTTTTGATGTGGGTGGTTACTTGACTGATAATCAATGGATTCAAAATCTTGGTGAAGATTATACCTCTCAATTACAAACTCTTCCTCTTTCTAGCATATCGCAGTCATCTTATAGCTCTTTGAATAGTGTAATCACAAGTGTGGATGGGCTGGTGGACTCTCCAAGCAGTACTGTTCAGCTCCCTGATTGGAATGATGAGAGCCAATCTATATGGCAGTTCAGGAAGGGGGTTGAGGAGGCTAGTAAATTTCTGCCTAGTGGAAATGAGTTGCTTGTTAATATGGAGATGAATCGATTGCTATCTCAGGATTCAAAGGGAGGGACTACTGAGGTGGCTGTGAAGGTGGAAAAAAAAGATGGGGGGGAAGATTGCTTGCCTAATGGATCAAGAGGAAGGAAGAATCCTTACAGGGAGGAAGAAGATTTAGAAGAAGAGAGAAGTAGCAAGCAGGCAGCCATTTTTCAAGAATCCACTGTGCGATCAGAGATGTTTGATAATGTTTTGCTTTGCTTTTTAGGAAAAGGTCAATCTCCTGTTGCAGCTCTTCGTGATGCCTTAAAGGATGGAACGAGCAAGCGTGTGCAGCAGAACGGACAGTCAAAAGGCTCTAATGCTGGAAAGGGTCGTGGTAAGAAACAAAGTGGTAAAAAAGAGGTTGTGGATTTAAGAACTCTTCTGATTCATTGTGCACAAGCTATTGCTGCTGATGATCGTAGGAGTGCAAATGAATTGCTAAAGCAGATTAGGCGGTATTCTTCCCCTTTTGGGGATGGAAGCCAAAGATTGGCTTATTGCTTTGCTGATGGCCTTGAGGCACGCTTGGCAGGTACTGGTAGCCAGATTTACAAAGGCCTCATTAGTAAGAGGACATCGGCTGCTGATATCTTGAAAGCTTATCACTTATATCTTGCTGCATGCCCTTTCAGAAAGATCTCCAATTTTACTGCGAAGCAAACAATAAAGATCTCAGCACAAAATTCCATGAGAGTTCATATCATAGACTTCGGTATCCTTTATGGTTTTCAGTGGCCTCCATTTATTCAAAGTCTTTCATTGAGACCTGGTGGACCTCCAAAGCTTCGAATCACTGGAATAGAATTTCCCTTACCTGGCTTCCGGCCAGCAGAGGGAGTTGAGGAAACAGGACGTCGCTTAGCAGATTATGCAAAGGAATTTGATGTGCCTTTCGAGTACAATGCCATAGCGAAAAGATGGGACACAGTTCAACTTGAGGAGCTCAAAATTGATAGGGATGAGTTTCTTGTTGTTAACTGTTTGTATCGAGCTAAGAACTTACTTGATGAAACTGTTGCAGTGGACAGCCCGAGAAACATATTTCTCAATTCGATAAGGAAGATTAATCCAGATATTTTTATTCATGGGATTGTCAATGGTGCTTTCAATGCTCCCTTCTTTGTCACCCGGTTCCGAGAAGCTCTGTTTCACTTCTCTGCAATGTTTGATATGCTTGAAACTATAGTACCCCGTGAAGATCCTGAGAGGATGCTCATTGAGAAAGAGATCTTTGGCAGGGAAGCCTTAAATATTATAGCCTGTGAGGGCTGGGAGAGAGTGGAGAGACCAGAGACTTACAAGCAGTGGCAAATCCGTAACCTAAGGGCAGGGTTTGTTCAAATTCCTTTGGACCGGGATATAGTGAAGAGGGTAACTGATAGAGTGAGGTCAAGTTACCACAAGGATTTTGTAATTGATGAAGATAGCCAGTGGCTATTGCAAGGATGGAAGGGGCGAATCATTTATGCCCTTTCAGCTTGGAAGCCTGCATAA

>LcGRAS45

ATGACTATGGATCCCAGGTTGAGGGGGTTTTCTGGTTCGCTTAATGGGGTCCAATTGAGTAATAAGCCGAATTCAGTCTTTCCAAATCAGAACATTGTTGCTGGAAGAATCGATAATGTCTTTCTAGATCACAATTTTAGGGACGATCGTTACCTTCAGTCTGATCCAACTCCCAGTAGTGTTGCCTCATGTCCAAGTGTGAACCATGAGGAAGATTCTCCGGAAGACTGTGATTTCTCGGATGCAGTTTTGAGATACATAAACCAGATGCTTATGGAAGAAGATATAGAGGATAAGACTTTCATGCTTCAGGAGTCGTTGGACCTTCAAGCCGCTGAGAAGTCATTTTATGATGTTCTTGGCAAGAAGTATCCACCTTCCCCTGAACCGAATTTGAGTTATACTGATCAAAATGGTGGGTACCTAGATGGTAATACCTCTAGAAATTCTGGAAACTGCAACACTAGTGGTTTTGATGTGGGTGGTTACTTGACTGATAATCAATGGATTCAAAATCTTGGTGAAGATTATACCTCTCAATTACAAACTCTTCCTCTTTCTAGCATATCGCAGTCATCTTATAGCTCTTTGAATAGTGTAATCACAAGTGTGGATGGGCTGGTGGACTCTCCAAGCAGTACTGTTCAGCTCCCTGATTGGAATGATGAGAGCCAATCTATATGGCAGTTCAGGAAGGGGGTTGAGGAGGCTAGTAAATTTCTGCCTAGTGGAAATGAGTTGCTTGTTAATATGGAGATGAATCGATTGCTATCTCAGGATTCAAAGGGAGGGACTACTGAGGTGGCTGTGAAGGTGGAAAAAAAAGATGGGGGGGAAGATTGCTTGCCTAATGGATCAAGGGGAAGGAAGAACCTTTACAGGGAGGAAGAAGATTTAGAAGAAGAGAGAAGTAGCAAGCAGGCAGCCATTTTTCAAGAATCCACTGTGCGATCAGAGATGTTTGATAATGTTTTGCTTTGCTTTTTAGGAAAAGGTCAATCTCCTGTTGCAGCTCTTCGTGATGCCTTAAAGGATGGAACGAGCAAGCGTGTGCAGCAGAACGGACAGTCAAAAGGCTCTAATGCTGGAAAGGGTCGTGGTAAGAAACAAAGTGGTAAAAAAGAGGTTGTGGATTTAAGAACTCTTCTGATTCATTGTGCACAAGCTATTGCTGCTGATGATCGTAGGAGTGCAAATGAATTGCTAAAGCAGATTAGGCGGTATTCTTCCCCTTTTGGGGATGGAAGCCAAAGATTGGCTTATTGCTTTGCTGATGGCCTTGAGGCACGCTTGGCAGGTACTGGTAGCCAGATTTACAAAGGCCTCATTAGTAAGAGGACATCGGCTGCTGATATCTTGAAAGCTTATCACTTATATCTTGCTGCATGCCCTTTCAGAAAGATCTCCAATTTTACTGCGAAGCAAACAATAAAGATCTCAGCACAAAATTCCATGAGAGTTCATATCATAGACTTCGGTATCCTCTATGGTTTTCAGTGGCCTACATTTATTCAAAGTCTTTCATTGAGACCTGGTGGACCTCCAAAGCTTCGAATCACTGGAATAGAATTTCCCTTACCTGGCTTCCGGCCAGCAGAGGGAGTTGAGGAAACAGGACGTCGCTTAGCAGATTATGCAAAGGAATTTGATGTGCCTTTCGAGTACAATGCCATAGCGAAAAGATGGGACACAGTTCAACTTGAGGAGCTCAAAATTGATAGGGATGAGTTTCTTGTTGTTAACTGTTTGTATCGAGCTAAGAACTTACTTGATGAAACTGTTGCAGTGGACAGCCCGAGAAACATATTTCTCAATTTGATAAGGAAGATTAATCCAGATATTTTTATTCATGGGATTGTCAATGGTGCTTTCAATGCTCCCTTCTTTGTCACCCGGTTCCGAGAAGCTCTGTTTCACTTCTCTGCAATGTTTGATATGCTTGAAACTATAGTACCCCGTGAAGATCCTGAGAGGATGCTCATTGAGAAAGAGATCTTTGGCAGGGAAGCCTTAAATATTATAGCCTGTGAGGGCTGGGAGAGAGTGGAGAGACCAGAGACTTACAAGCAGTGGCAAATCCGTAACCTAAGGGCAGGGTTTGTTCAAATTCCTTTGGACCGGGATATAGTGAAGAGGGTAACTGATAGAGTGAGGTCAAGTTACCACAAGGATTTTGTAATTGATGAAGATAGCCAGTGGCTATTGCAAGGATGGAAGGGGCGAATCATTTATGCCCTTTCAGCTTGGAAGCCTGCATAA

>LcGRAS46

ATGCTTCAGAGCTTAGTCCCACAATCTTCAATAAATTCTACTGCCTCCTCCGCCATGAAAACCAAGCGCCTCGACCGGGAAAGCGACGACGCCAGCGACGATCGTTCCAGCAAACGCCCCAAGGAGACTCAGGAGGAGCAGGTGCAAGTCGTGGAAGAGCAAGCGGTGGTGATGGAAGGAGAATCGACGGGACTGAGACTCCTAGGGTTGTTGCTTCAATGCGCCGAGTGCGTCGCCATGGACAATCTCGATGACGCCACCGAGCTCTTGCCAGAGATCTCCGAGCTGTCGTCTCCGTACGGGTCGTCTCCGGAGCGCGTCGGAGCGTACTTCGGGCACGCGCTCCAGGCGCGCGTGGTGAGCTCCTGTTTGGGTAGCTACTCGCCTCTTACGACGAAGTCGCTGACTCTGTCTCAGTCTCAGAAGATCTTCAACGCTTTGCAATCCTACAATTCCATCTGTCCGTTGATCAAGTTCTCTCACTTCACCGCCAATCAAGCCATTTTTCAAGCGTTGGAAGGCGAGGATTGCGTCCACGTCATCGATCTGGATATCATGCAAGGCCTTCAATGGCCGGGATTGTTCCACATCCTCGCCTCCCGGTCTAAAAAGATCCGGTCCATGAGAGTAACCGGGTTCGGTTCGTCGTCAGAGCTACTCGAATCGACAGGCAGACGACTGGCCGACTTCGCTACCTCGCTCGGGCTGCCATTCGAGTTTCAACCACTGGAGGGCAAAATAGGAAATGTGAGGGATTTGAGTCAACTCGGGGTGAAACCGAATGAGGCTATAGTCGTACATTGGATGCATCATTGTTTATACGACATAACGGGGAGCGATTTGGGGACGTTGAGATTGTTGACTTTGTTGAGGCCGAAATTGATCACGACCGTTGAACAGGATTTGAGTCATGCGGGTAGTTTTTTGGGTAGGTTCGTGGAGGCCTTGCATTATTATAGTGCTATGTTTGATGCGCTGGGAGACAAATTAGGTGCGGACAGTGTGGAGAGGCATACGGTGGAGCAGCAGCTGTTCGGTTGTGAGATTAGAAATATAGTGGCAGTCGGTGGGCCCAAAAGGACGGGTGAAGTGAAAGTGGAGAGATGGGGTGATGAGTTGAGACGGGTCGGGTTTAAACCCGTTTCGCTTAGTGGTAACCCGGCTGCGCAAGCCAGTTTGTTACTAAGTATGTTCCCTTGGAAAGGGTATACACTGGTGGAGGAAAATGGGTGTTTAAAATTGGGTTGGAAAGATTTGTCTTTGTTGACTGCCTCTGCTTGGCAGCCGTCCGATTAA

>LcGRAS47

ATGAGTTCATTTAAACCTCCATCATTGAAGCCTGATGTGACATTGTCCCTGACATTGTCATCACCGTGCCCAAGTCCACAGGAAGCCCTGAAGCCTGAAGAAAGAGGCATACGTCTAATCCAACTCCTCCTAACATGTGCCAAGCATGCATCCTCCGGCAACCTCCACCGCGCCGATGAGTGTCTACAGCAGATATCGGAACTTGCCTCGGTGTCCGGGGACTCCATGCAAAGGCTTGCAGCCCGTTTTGCATCTGGCCTTGCCGTCCGTTTAGTCAAGCGTTGGCCTGGCCTCTACAAGGCCTTGAACCACATGCAACAGCCTAAAGAAGAAGAACATGAAGATCGCCCTCGGCGTCTCTTTTCTCAATCCTTCCCATACCTTGGGTTCTCATACGCTATCATCGCTCGAACACTGTTCCAGCTCGTGACTACTGAACGTGTGATCCACATCATCGATTTGGGCTCCGGAGACCCAAAGTTTTGGATTCCGCTTATCAGGAACTTCTCAAGTTTGGCAGAGGGACCTCCCCATTTGAAGGTCAGTTGTGTTAGTTGTAACAAGCATGTTTTAGACAAACTAGGCCCAAGACTTGTAAAAGAGGCTGAAGCCCTGGACATGGCTTTTCAATTCAACCCCATCAATGTCAACTTAAGAGAGTTGAACATAGACATGCTCAATGTTAGATCAGGAGAAGCATTGGCTTTCATTTCAGTATTAAATCTTCATGCTTTATTAGCCCAAGACGATAGAGTTGATGCACATTTTGGGTTCAATAATAAGAGTGACAATGTCAAGGATTGTAAGCAGTTGGGTCAATTTTTGGGCATGATTAGATCAATTGCACCCAAGTTATTCTTTTTGGTGGAGCAAGAAGCTGACCACAATCTAAATCGGTTAGTGGACAGATTTGTTGAAGGGTTGCATTATTACAGTGCAATTTTTGATGCAATAGATGCCACATTTGGGTCCAAGACAAGCGAGGAAAGACTTATATTAGAGGAAATGTTTGGGAGAGAAATTGAAAACGTTGTTGCAAGTGAAGGATTAGAGAGAGAGGAGAGGCATGAGAGATATGGTCGGTGGATGGTTCGGTTCGGTCAAGCAGGGTTCAAGCCTGTGCCTCTTTGGGCTGACCCTATAGAGCATGGCAAGCAAGTGGTCGAGGCCTATGGTAAGGATGGTTATAAGATAATAAATGAGAGGGCCAGTTTGATGATTTGTTGGTATGACAGACCCCTGTATGCTGTGTCGGCATGGACATGTTGA

>LcGRAS32

ATGAAAACTGAAGTTAGAGGAAACACTACGTCCATTTCTCTGCAGAACCCTAGCCTTTTCAACGCCCCACAAAGCTCTCTCTCAGGAGCACTCAGAGGGTGTCTCGGCAGCCTGGACGGAGCATGTATAGAGAAGCTGCTGCTCCACTGCGCCAGTGCCTTGGAAAACAACGACGTCACTTTGGCACAGCAAGTGATGTGGGTGCTCAACAATGTTGCTTCTTCGGTTGGTGACCCTAACCAGAGGCTCGCCTCATGCTTTTTAAGGGCACTCGTTTCCAGGGCATCTAGGGTTTGCCCAACAACGATGAGTTTCAATGGTAGCGGTACGATTCAGAGAGGTCCAATGAGCGTGACCCAGCTAGCCGGGTACGTTGATCTAATCCCTTGGCACCGATTTGGGTTCTGTGCGTCAAATAGTGCTATCTTTAAGGCAGTTCAAGGTTGCCCAAAAGTTCATATTTTAGATTTTAGCATCACTCATTGTATGCAATGGCCTACTCTCATAGATGCTTTAGCGAAAAGGCCAGAAGGGCCTCCTTTGCTTCGAATCACCTTGCCTTCTTGTAGACCACCAGTCCCTCCTTTGCTCAACGTGTCCACCGAAGAGGTCGGTCTCCGTTTGGGGAATTTTGCAAAGTTTAAAGATGTTCCCTTTGAATTTAATGTGATGGATGACTCTGCTTCCACATCAGGTGAGATTATTGTGTCCAAAGAATCCTCTTCTTTTCACTTTGAATCACTTTTGAGTCACTTGACTCCTTCCATGTTGAACCTTAGAGATGATGAGGCTTTGGTGATAAACTGCCAAAATTGGATTCGATATTTTTCTGATGAAACAAACGCTCTTGATGCTTCTTTGAGAGATGCTTTTCTCACTATAATTAAAGGACTTAACCCTAGTATCATCGTAATAGTCGATGAGGATTCTGATTTAAGTGCTTCTAGTCTCACTTCAAGAATCACTACTTGCTTCAATTATCTTTGGATACCCTTTGATGCTCTGGAAACTTTCTTGCCTAAGGATAGTTGCCAAAGGCTAGAATACGAGTCCGATATCGGTCACAAGATCGAAAACATCATAAGCTTTGAAGGGTTTCAAAGAATAGAGAGGCTAGAATCCGGGGCCAAACTGACTCAAAGAATGAAAAATGCTGGCTACATCAGTCTCCCGTTTTGTGAGGAGACTGTTAGCGAAGTTAAGTCTTTACTAGATGAACATGCTGGTGGATGGGGCATGAAGAGAGAAGAAGAGATGTTGGTGCTCACATGGAAGGGTCACAACTCAGTTTTTGCAACAGCTTGGGTCCCAAATGGGCTGGAGGATTGA

>LcGRAS25

ATGTTGGCAGGGTGTTCTAGTTCTACATTGCTGTCACCAAGGCACAGATTGAGGAGTGAAGCACCTGCACAATTTCAAGCTTGCCATTTTCAGTTACCTTCGATGAGCACACAGAGACTGGACTTGCCATGTAGCTTCTCTAGAAAAGAATCTTCGCGATCGCAGCCCATTAGGCCTGTTGGTCTCTCTGTTGAAAAAGCTATTGAACCAAAGACTAGCAGTTGTTCTCTGAAGCAGAATATTCGCCTACCGCCTTTGGCTACTAGCACTCCGAGAGAGGTCACAGATGAGTTCTGGGAGAAGGGTAAGAGTCTGAAGAGGTTCGCTGAGGGAGGCCTGGTTGATGATTCTTGCATTAATAGGGCTAAAAGGAAAAAGGGAGGCAGTGATGATGCCAGACAAGATGATATTCATGAAGGTGGTGGTGATAGTTTGAGTTTAGGCCAATTGGGTTCTGGCAACTTCTGGTTTCAGCCAAGTTTCCCTGGCCAAAATATCCCTCAAGTTCCCTTTTCTCTGACTTCCTCAGGAGGGGAAGACAGGGTTTGTTTTGTGCCTAGTGAATTGATTTCACCACCTTTGCCATTGTCAAACAATCCTTGGGTGGAATCAGTCATAACTGAGATCACCGATTTTGGTGAAAAGGATGGTGATGAGACTAGTCAGGGGCTAGGAAAGGAGGTTTCGGGTTCTAGTACCTCATCGGAGAGTCATAGCTTAAGCCTCAGGCTACATGAGAATCCGGTGGAGCATGATGTTGGTAATGGTTCTAGGAATCCCTATCCACATCAAGGTGCTGGTGTGGGTGTGGGTGTGGATGCTAGTGAAGAAGATAACAACCAAGAAGAGACTCGAGGATTGGAGCTTGTCAGCTTACTTACAACTTGCGTGGAAGCAATTGGGTCAAGGAATATAGCAGCCATCAACCATTTTATTGCTAAGTTGGGGGATCTTGCTTCTCCAAGAGGAAGCCCGATAAGTCGTCTAACTGCTTACTATGCGGAAGCTTTGGCTCTTCGAGTCACAAGGCTTTGGCCTCACACATTTCACATTACTCCTCCTAGAGATCTTGATCGGTTGGATGATGATTCGGGCACTGCATTGAGGCTTCTAAACCAGGTGACCCCAATTCCAAAGTTCATTCATTTCACGTCAAATGAGATACTGTTGAGAGCTTTTGAAGGAAAAGACAGGGTTCACATTATTGATTTTGACATCAAGCAAGGGCTTCAATGGCCTAGCTTCTTTCAGAGTTTAGCTTCCAGGACTAATCCTCCAAGCCATGTTAGAATCACTGGTATTGGTGAGTCCAAGCAAGAACTAAACGAAACAGGAGATAGGCTGTCTGGATTTGCTGAGGCATTGAATTTGCCTTTCGAGTTCCATCCAGTTGTGGACAGGTTGGAGGATGTGAGGCTGTGGATGCTTCATGTGAAGGAGAAAGAAAGTGTGGCAGTGAATTGTGTTTTTCAGTTGCACAAGACGCTGTATAGTGGGAATGGAGGAGCATTCAGAGACCTTTTGGGACTTATCAGGAGCACAAATCCTACACTAGTCCTTATGGCAGAGCAAGAAGCTGAGCACAATTCCCTTAACTTGGAGACAAGAGTTTCCAACTCATTGAGATACTACGCTGCCATTTTCGACTTGATTAATTCTTCCCTCCCACTAGACAGTCCAGTCAGGATCAAAATAGAAGAGATGTTTGCACGGGAGATCAGGAACATTGTTGCTTGTGAAGGAAGTGATCGGTCTGAAAGGCATGACTGTTTTGGCAATTGGAGGAAGATGATGGAGCAAGGAGGTTTTCGAAGCATGGGGATCAGCGAGAGGGAATTTCTTCAGAGCCAAATGTTATTGAAGATGTACTCTAATGAGAATTACAGCGTTAGGAAGCAAGAGCAGGATGGAGCAGCAGCGCTTACTTTGAGCTGGCTAGATCAGTCTCTTTACACAGTCTCAGCGTGGGCACCTGTTTGA

>LcGRAS33

ATGGATTCGCAGTTCACTGAATTTAACAGTTCAATATATGGTTTCAATGGGGAGCGTGATACTGTTTTTCCCAATTCGAATGAGTGCCTGGATGTTGCTAATGAGTTCAAATTCAATGATTCTAATTTAGATGCAAGTTTTGTGGATGATTTGCTAGTAACACCTGAACGGGACTCTGGCTTTTCTGCTCAGTCGTTTAGTTTGAGTCCAGAGGGAGAGTCTTCTTCTCCCTCTGATGATAATGACTTCTCTGAAACTGTTCTTAATTACATCAGCCAGATGCTTATGGAAGAGGACATGGCAGAGAAACCGTGTATGTTTCATGATCCGTTGGCTCTCCAAGCCAAAGAGCAGTCACTATATGAAGTCCTTGGTGAGAAGGGCTCTTATTCTCCCGATCAACCCCCGTTTTACAACACTGAGAGCCCAGATGGTTATTTTTCTAGTGGTTTTAGTGATCATGGTAGTAATAGTAGTTCTAGTTCCGGTACAAACCACTTGGTTGAACCGTTCTGGAATGGTGATTACAGGGAATTCAGGCCCGCATTGTTGCAAATGCCTATTCCTATTAACTTTGTGTTTCAGTCCACAGCCAAGTCTAGCTCGCAGACTACAGCCAAGTCTAGCTCGCAGACTTCAAATAAATTTCAAAATGGATTTGCTAATAATGGGAATGTGTTTGCAGCATCTGCTGCAAGTGAACTTCAGATTCCGGACTTGTTTAGTCAGAGTGACTTGATGTCTCAGTTTAGGAGAGGCGAAGAGGAAGCTAGTAAGTTCCTTCCTAAAGTTACTTCATTGGTTATTGATCTGGAGATTGATAAGTTTGACCCAGAATTGAAGGAAACGGCTACAAAGGTAGTTGTGAAGGCTGAGAATGATGAGAGAGAGCATTTGTCTTCAGGGTTGAGGGAAAAAAAGAATCGTGAACGAGAAGATTTGTATTTAGAAGAAGAGAGGAGTAACAAACAATCAGCAGTTGACCTGGAAGAGACTGAGCAGCTATCAGAGATGTTTGATAAGTTGTTAATCTGTCACGAAAAGAATGGTTCTACATGTTTTCCTGATAATACCCTTCCACATGATGGATCAAGAAAGGCAGTGCAGCAAAATGGGCCAATGAAAGGGTCTAGTGCTGAGAAGAACTACACGAAGAAACCTGTGAATAAAAAAGAAGTGGATTTGAGGACTCTACTGATCCAGTGTGCACAAGCAGTCTCAGCTGATGATCGCAGGACTGCTAACGAGATGCTGAAGCAGATTAGGCAGCATTCTTCACCTTTTGGTGATGGAACTCAGAGAGTGGCCCATTGCTTTGCCAATAGCCTTGAAGCACGCTTGGCTGGCACTGGAGCCCAGATTTATACTGCTCTGTCTTCCAAAAGGACATCAGCTGCTGAGATGTTGAAAGCTTATCATGTTTATATGGAAGCCTGCCCATTTAAGAAGATTGCGATCGTCTTTGCAAACCATACTTTTCTGGAGTTAGCTGAGAAAGCAACAACACTGCATATCATTGATTTTGGTATCTTGTATGGTTTCCAATGGCCTGCCCTCATTTTCTGTCTTTCAAAGAGACAGGGTGGACCTCCTAAGCTACGTATTACAGGGATAGAGCTTCCTCAGCGTGGTTTCCGGCCAGCAGAAAGGGTTGAGGCGACGGGGCGTCGCTTGGCAAAGTACTGTGAGCGCTTCAATGTTCCATTTGAGTACAACGCCATAGCCAAGAAATGGGAAAATATCCGAATTGAAGACCTTAAAATTAAGGAAAATGAAGTCGTTGCTGTGAATTGTTTGTTCCGGTTTAAGAATCTGCTTGATGAGACCGTTGTGGTGAATAGTCCTAGGAATTCTGTGCTAAACTTAATCAGAAAGATCAAACCAGATATTTTTATCCAAGCTATTGTGAATGGATCCTACAATGCCCCTTTTTTTGTCACACGGTTCCGGGAGGCACTATTCCATTTCTCTGCATTGTTTGATATGTGGGATACGAATATTCCCCGCGAAGATCAAATGAGATTAATGTTTGAGAAAGAATTCTATGGGCGGGAAGTTATAAATGTTGTAGCATGTGAGGGCTTGGAGAGGGTGGAGAGACCTGAGACCTACAAACAATGGCAGGTTCGAAATACAAGGGCTGGGTTCAAGCAGCTGCCCGTGGGCCCTCAACTCATGAAAAAGTTGAGATGTAAGGCTACTGGTTATCACGATGACTTCATGGTCGATCAGGATGGACAATGGATGCTGCAGGGGTGGAAAGGCCGAATTATCTATGCTTCCTCTGCTTGGGTACCTGCATAG

>LcGRAS34

ATGGATCCAAATGCTACTGGATTCTCTGATTCTGTTAATGGTTTCAAGCTTTATGATGGAACCCTTTTGGCCTATTCTAATCAATTTTCGGACATCGAAAATGGATCTAAGTTCACCATCCCTTCACCAGATTTCAACCTTTCAGATGTCCCTTATAGTGCTCTTGATCCAGATCCTGGTATATTTACTCCATCTTCAACCCTGAGCCAAGATTTTGACTCTGTGGGGTCTGTCAGTTTGAGCCCAGATGGAAGCTCTTTCGCTCCGACTTCGGGCTGGAGTCCGGAGGGAGTAGCTTCTTCTTCCTCTGATGATAGTGAATCCTCTGATCCCCTTCTTAAGTACATCGGCCAGATGCTTATGGAAGAGGACATTGAAGACAAGCCATCTATGTTTTATGATCCGTTGGCTCTACAAGCCACTGAAAAATCTTTGTATGATGTGCTTGGTGAGCAGCAGCAAAACTATCCTTTGCTGCAAAATCAACCACAATTATATGTCAATGATAGCATAGGGAGTTACTCTAGTTTTTCTGGTAGTATCATTGATTTTAGTGGTAATTCTGGTGGAGTTACTAGTACTAATAGTGGTACTAGTGATTTTGTTGATACCCTATTTAATGGTGATGTTGGAGAGCTTAATCCCCTGTTGTTGCGAGGCTCTTTGCCTGGTAACTATCAACTCGAGTCCAATTCGCAGCAGCCCAACTCACAATATTCTGTCAGTTTGCCGGAAAGTGCGGCAAATATGGGCGATGGGTTAATGAGTTCTTCTGTGAATGAGCTTCTGGCTCAAAATATGTTTAGTGATAAGGAGTCTATGCTGCAGTTTAAGAGAGGGTTGGAGGAAGCTAGCAAGTTCCTTCCTACTAGTAATCAGCTAGTTATTGATGTGGAAAACTACAATTTTTCCGAGGAGCAAAAGAAAGAGACTTCCATGGTGGCTGTCAAAGTGGAGAGAGATGAAAGGGAAAACTCACCTGAACGGTTAAGGAGTAGAAAGAATCATGCACGGGGGAATCCAGATTTAGAAGAAGAGAGGAGTAACAAGCAGTCAGCTGTCTCTAGGGAAGAGAGTGAACTGTCAGAAATGTTTGATAAGGTGTTGCTGATTTGCACCGATGCAAATGGTAAGCCTTTGTTGTGCCATGCCGAGGATCCTGTTGAAGCTGCGCAGAATGGGGCGAGCAACAGTTTGCAGCAAAATGGACAAACAAATGGATCTAATGGTGGGAAATCCCGTGGCAGGAAGCAGGGAAAGAAGGAAACAGTGGATTTGAGGACTCTTCTCATTCTCTGTGCACAAGCCGTGTCCACTAATGACTTCAGGACTGCTAATGAGCTACTAAAGCAGATTCGGCAACACTCTTCCCCTACTGGTGATGGATCCCAGAGGCTGGCTCATTTCTTTGCCAATGGTCTTGAAGCGCGCTTGGCTGGCAGTGGTATGGCAAATAAAACTTTCTTCACTTCCTTTGCTCAAAAGACTACGACTGCTGATATTTTGAAATGTTATAAGGTTCATCTTTCTGCTTGTCCATTTAAGAAGCTGTCCAATTTGTTTGCAAACAAAATGTTTATGCATATGGCTGAGAATTCACCAACTCTTCACATTGTTGATTTTGGTATCCTATATGGTTTCCAATGGCCATCTCTCATTCAGCTTCTGTCAATGAGACCTGGTGGCCCTCCTAAGCTACGGATTACTGGGATAGAGTTTCCACAACCTGGTTTCCGGCCAGCAGAAAGAATTGAGGAGACAGGTCATCGTTTGGCAAAGTATTGTGAGCGCTTTAATGTTCCATTTGAGTACAATGGCATAGCATCACAAAACTGGGAAACCATCCGAATTGAGGACTTCAAGATTAGAACTGGGGAGGTGCTTGCTGTGAACTGTCTGTCCCGGTTTAAGTACCTACTTGATGAAACTGTTGAAGTGGACTGTCCAAGGAATGCTGTCTTGAGCTTGATCAGAAAGATGAACCCAGATGTTTTCATCAATGCTATTGTCAATGGTGCCTATAATACTCCCTTCTTTGTTACACGATTCCGGGAAGCACTGTTCCACTTCTCCTCGCTGTTTGATATGTGGGATACTACTATACCCCGTGACAACCAAGAGAGGTTGATGTTTGAGAGAGAGTTTTATGGTCGAGATATTATCAATACCATTGCATCTGAGGGCTTGGAGAGGGTTGAGAGGCCTGAGACATACAAGCAGTGGCAGGTGCGGATCACGAGGGCTGGGTTCAAGCAGCTGCCATTGGACCGAGAGCTGCTGAATAAGCTTCAAATGAAGTTGAAGGCTTGGAATTACCACAAGGACTTTGTGATTGATGAAGACAAGCATTGGATGCTGCAGGGATGGAAGGGAAGGATTGTGTGTGCCACCGCCTGTTGGGTACCTGCATAG

>LcGRAS35

ATGGAGAGTGACTTCCCAGATGCTGTCCTGAAGTACATAAACCAAATTCTCATGGAAGAAGACTTGGAGGAGGAAACCTGCACATTCCAGAACTCAGCTCTTCAGGCCGCTGAGAAATCCTTTCATGATATTCTTACCGTGAAATATCCTTCAACCAGTCGAACACCTTCAAGCCGATGGGAAGAGAACCGAATTTACAATTGTAATCGCTGTTGCAATTGTAACGAAACAGATTTTGTTAAATCCCGCTTGATTACTAGTCTTGGAGGTGGTGAACTAGGTGCACCAGTTGAGTACTCCTTCCAGTTCAATCTGCAGTCCAGTCGAAGGTGCTTCCTGCCTAGCAGCCTAAGCAATGCTGTTGAAAGGAAAGTTGATTTCCCCATAGACATACCCATAGTGTCTAATTACTTTGGTGAGTGTGAAACAGGTTTGCAGTTCAAGGATTGCACACAATATGGTCAAACTTCCCTTCCACAATCCAATCATGTCATTGATTTTCAAAACAACAGAACCAGCGTGCTGGAACTTAATGGAGAAAATCCTGCTGTGGTGGCTGAGGCTAAGAGACCTAAGAGGGTTCACTTACTTCGTAAACTGAGGGATCCTGATCAGCTAGAGAGTGAGCTAGAAATGAGGAGTAATAAGCATTTGAAAGTTTATGCTGACGAACCTGAAAAACTTGAGGTGTTAGATAAGATGTTGTTGCCAATTGCTGGAGATGAGGAGTCTACTAGGTTTGCAACTGATGACAGCCTGAAAAGGAAGCCAATCAGGACTTTGCATCAGTATAGAGTATCAAGAGTATCGAATAGTGGAGCCAACTGCAGGACGAAACGAGTTAAAAACAGAATGATTGTTGATTTGAGGACTCTACTAATTCATTGTGCAGAAGCTGTGGCGGAAAATGATTGCCAAAGTGCGAATGAACGATTGGTGCAGATCAAACAGTATTCTTCTCCTTTTGGAGATGGATCACAGAGGCTTGCTCATTGCTTTGTCAATGCGCTTGAGGCTCGCCTTGCAGGCATAGGAAGCGAGGTATATGCAGCTCTTGCGGCTAAGAGGGTGGAGGCCACTTACATGCTGAAAGCCTGCAGGTTCTATATCACAACATGCCCTTTCATGAAGATCTCAAATTTCTTTGCTACCCAAACAATCATGAAACTGGCTGAGAAAGCAAGCGTGCTCCACATTATACATTTTGGTATAATGTATGGTTTCCAATGGCCCTGTCTTATTCAGTGTCTCGCAACAAGACATGGTGGACCTCCAGTGCTTCGCATTACAGGAATAGACCTTCCACAGCCTGGTTTCTGTCCAGCAGCCAGGGTCAAGGAAACTGGACGTTACTTGGTGAAATACTGTGAGAGATTTGGCGTTCCGTTCAAGTACAATTTCATAGCACAAAAGTGGGAGAACATTCAACTTGAGGACCTAAAGATTAACAGAGATGAGGTAACTGTTGTTAACTGTTTGTACATGTTGCGGTATCTATTGGATGAGACAGTAGTGCCTAACAGCCCCAGAGATGCTGTTTTGAATTTGATTAAGAGGATTAACCCAGATGCTTTCATTCATGGGATCATAAATGGAGCCTACGATGCCCCCTTCTTCATGTCACGGTTTAGGGAGGCACTTTTCTACTTCTCTGCAATGTTTGACATATTCGATGCCAATGCAGGCTGCGAGAGTTGGGAGAGGATGGTGTTTGAGGAAGAGATATATGCAAAGGAGATAATGAATGTGATAGCATGTGAAGGAACAAAGAGGATTGAGAGGCCGGAGACATACAAGCAGTGGCAGCTTCAGAACTTGAGGGCCGGTTTCAGACAGCTTCCACTAAATCAGGAGATCATGAAGAAAGCAAAGGCCCAAGTGAAGTTAAATTATCACAAAGATTTTGCGCTGGATGAAGATAGCCAATGGATGCTACAAGGATGGAAGGGGAGAATTCTTTTTGCTATCTCCTGCTGGAAATCTGCTTAG

>LcGRAS36

ATGAATGGATTTAGTTTGCGCCATGAGAGCTTAGATGGGGTCGGTTCAAATGATGAACCTGTTGATGTGTCAGACTCAATAATGAATGGATCCATGGATCCAACCAATCAACACCCTACCTGCAGAGAATTGGATGCATCTGCAATCAACTATGAAAATACCAACTTCTCCTTTGCTGTTGGCAAGTACATTTATGATATGCTCATGGAAGAAAATGTAGAGGATCAGACCTGTATGTTTCAGGATTGTTTAGCCCTCCAAGCCACTGAAAAGTCCTTCTATGACGTTCTTGGCCAGAAATATCCACCTTCTTCTGATCAATCTTCTCTTTTTGCCAATCCAAATGACAACACCCCAGATGATGTTTTCATTTGTCGTACAAATATTGATACCAACAGTGGCTATACTGCTTCCAACCACCTTGTTGATTACAGTTCAATTTGTAACCAAGCCGAGCCTGGATCCTGTCATCCACCAACTTCTCTGGTTGACTCTCCTGAAAGTGCTTTTCCAGCAGCAAGTGGTGATGGTGAAAATGAGATACTTAAGCCAGTAGAAGTGCCAGAGAAAGAGGGGAGGGGAAGAAAAAATCGTCACAGTGAAGATATCAATGACTTGGAAGGGAGGAAGAGCAGCAAGCGTTCAGTGCCTTCAGTTGAGGAATCTGAGCAATTAGACCTGTTTGATGAGGTGATGATTGCTAAAGGTGGGAGCTATGATGCTGTAGAATGTCCTCTCTTCAACGCAGGGCGCAATAGAGCCTGTATGGAATTGGACCGCGTTGAGCGAAGACATGGTTCCAACAGCAGAATAATGCATATGATGAGACAAAATAATACAAATGAAACTGTGGATTTGTGGAGTCTACTAACTCAATGTTCACAAGCTGTCGCAAGTGATGACCAAAGGACTGAAACTGAGCTACTAAAGCAAATTAGGCTGCACTCTTCTCCCCTTGGTGATGGAACCGAAAGATTGGCCCATTACTTTGCTGAGGGCCTTGAGGCACGCATGGCAGGCACCGGGACTCCATTCTATTCGCCTGCCTTTCTTAATAGATCTTCTGTTGCTGATATGTTGAAAGCTTACCTTGCATATGTCTCGGCAGCTCCTTTCAAAAGAGTCTCCAATTTCACTGCAAATCGTGTCATTGGGAAGCTTGCAGAGAAAGCAACAAGGGTGCACATTATAGATTTTGGAATTTCTTATGGTTTTCAATGGCCTTGTTTCATCCAGCGCCAGTCATTCAGGCCTGGTGGACCTCCTAAGATTCGGATAACAGGAATCGAGTTTCCTCAACTAGGCTTTAGGCCTGCAGTGAGGGTTGAAGAGACGGGGCATCGCTTGAAAAGAGTTTCTGAGGCATGTAATGTGCCATTTGAGTACAATGCCATAGCTCAGAAATGGGAAACTATCAAACTTGAGGATCTCAGGATTGACAAAAATGAAGTGATTGTTGTTACTTGTATGTATAGGTTAGACAACCTACCTGCTGACACAGTTGTGCTCAATAGCCCTAGAGATGCTGTCCTGAAATTGATCAAGAGAATTAATCCTGATATGTTTGTCCATGGAGTCACCAATGGGACACACAATTCTCCATTTTTCAAAACAAGATTCAGGGAGGCACTTTTCCATTTCTCTGCACTGTTTGACATGTTTGAGGCTACTGTACCAAAAGAAGACCAAGGGAGGCTGATTTTTGAAAGAGAAGTAATTGGAAGACATGCCATAAATGTTGTAGCTTGTGAAGGCTTGGAGAGAATCAATAGTCCGGAAACATATAGGCAATGGCAGGCTAGGAACCTGAGGGCTGGTTTCAGGCAGCTCCAACTGGACCAGGAGCTCTACAAGGGAGCTCGGAAACTTGTGAAAATGGATTATCACAAGCACTTTGTTGTTGATCAGGATGGCCAATGGTTGCTGCAGGGATGGAAGGGAAAAATAATCCAAGCTCTTTCCTTCTGGAAACCAATTCAAGACTAA

>LcGRAS37

ATGGATACCCTTTTTGAAGGATTTCCAGGATCAGTGAATGGATTCAAGTTTGATTGTGTCAGAGAGTTTACACAGAGTCATGATTTTGGAGGTCATCCTCATCCACCAACACCGCCACCTTTACTTCAAACCAATCCAAATCCTGTAGCTAATTGTTTCCAGGTTTCAAGCACCAGCTCTGATGGGGACTCCCCGGAGAGCAGTGACATCACTAATGAGGTTCTCAGGTTCATAAACGAGATGCTCATGGAAGAAGACCTGGAAGGAAAAACCTGTATGTTGCAGGATTGTTTGGCTCTCCAAGCTGCTGAAAAGTCCTTATATGATGTTCTTGGCCAGAAATACCCTCCTTCACCAAGCCATGTCCTGCCTTGTTTGAACCAAAATATTGACACCCCGGATGAGATTTTCATCAGTGAGAGCTATGATACTGCTGGATACTTGGTTGATTCCCCAGAAAGTGTCTCCTTCAGTGGAGGACTTGGAGAAAGTAGCAGTTCTGTTTCGAATAGATCAAGAGGAAGGAAAAACCATCAAAGGGACGATAGTGATCATATTGAACAAGGGAGAAGCAACAAGCATTCAGCGATTTCGCTTCAGGAGCCTGAGGAATCAGAGATGTTTGATGAAGTACTGCTCTGCAAGAGTGAGGATGATGAGCCTGGATCACGTTCTCACCGCGAAGAGTTGCTGAATGGATCAAACAGGAAGCTGAAGAGCTCAAGTAGTGGAAAAACAAGCACAAAGAAGAGAGGTAAGAGAAGGGAGGTGGTTGATTTGTGGTCCCTATTAACTCAATGTGCACAGGCTGTGGCTAGTCTTGATCAAAGAAGTGCAAATGAGTTATTGAAGCAAATCAGGCAACACTCTTCTGCCTTTGGCGATGGAACACAAAGATTAGCACATTACTTTGCTAATGGACTCGAGGCACGTTTGGTTGGAATTCAAACACCTATACATACACATATAAGTAGCCGGGCATCAGCAGCTGATATCTTGAAAGCTTACAAAGTCTGTGTCTCAGCAACTCCTTTCCATTTTGTGTCATTTTTTATGGCAAACCAAATGATCCTCAAACTTGCAGAGAAAGCAACAAGGCTGCACATTATTGATTTTGGTATCTGCTATGGTTTTCAATGGCCTTGCTTCATTCAACACCTCTCCAAAAGGGCTAATGGACCTCCTATGCTTCGAATCACAGGCGTTGAGCTTCCCCAGCCAGGATTTAGGCCTGCAGAGAGGGTTGAAGAGACAGGGAGACGGTTAAAAGGCTATTGTGAGAGATTCAAGGTTCCATTTGAATACAATGCCATAGCAAAGAAATGGGAAACTATCAAGTTGGAAGATTTCAAGCTTGACAAAGATGAGGTGATTGTTGTTAACTGCTTGAAGAGAATGAAGAACCTGCCTGATGACACAGTACTGGCAAGCAACAGCCCGAGGGATACTGTCTTGAACCTGATCAATACAATCCATCCTGATGTGTTCATCCATGGAGTTGACAATGGTACTCACAGTGCACCTTTTTTCCTCACAAGATTCAGGGAAGCACTCTTCTATTTCTCGGCATTCTATGACATGTTTGAGGCTACCGTGTCCCGCGAAGATGAGGGCAGGATGCTGTTTGAGAGAGAAATATTTGGAAATGACGCCATGAATGCCATTGCCTGTGAGGGCACAGAGAGGGTTGAAAGGCCAGAGACATATAAACAGTGGAGGGTCAGAAGCCTGAGAGCTGGTTTCAGGCAGCTCCCATTGGATCAGGAGCTTGTGAAGAAAGCTAAGAAAAAGGTGAAATCACACTATCATCAGGATTTTGTTATAGATGAGGATGGCCATTGGATGCTCCTGGGTTGGAAGGGAAAAATTCTCCAAGCTCTATCTTTCTGGAAACCTGTCCTGGATTGA

>LcGRAS38

ATGAATCATTTGCAATTTGACCATGGTTTAGGACCTTTAGGTTCAGTTTATTCAAACCAAAATCACGTTGATGGGTTCAAAAATGATGGTGATAAGACTCATCATGAAGACCCTTCTCACCCACTTGAAAACAATACACACCAAACTAAGGACTCAGGTCCATGTTCTGGTGGGAGCTCAGAGGGGGTTTCTTCGGATTTTTGTATTGAGCTGAACCATGCAACTCTTAAGTACATAAGTGATATGCTTATGGAAGAAGATCTGGAGGGTAAGACATGTATGTTGCAGGATTGTTTGGCCCTCCAAGCTGCTGAGAAACCCTTCTATGAAATACTTGGCCAGGAATACCCGCCTTTGCTGAATCAAATGTCTTGTTATCTGAATCAAAACTTGGAGAATGCGGATGATTGTTGTACGACTAATAGTCGTGTTGATAGTAGTAACAGCATTGTTACTGCTAGCAACTTGGTTGATCCCGATTGGGTTACTAGTCAGGGCAAATTCAAGTCCTCTTGGATGATTCAGACCTCCCCTATTGACTCTCCTGACAGTGCTGCTCTGGTGCCAAATTTATGTGGGGAGAGACAGATAAGTGGACAGCTAGGTGGGCCTAGAAAGTCCCTTCCAAAGGATGATTCTTTGAGCAATCCATCAAGGCCTCGAGAAGAGGCGGTTGACAGTTCAGCAACAGCAGGGAGTTACAATTTGTCGTATGGGTCAAGGGGAAGGAAAAGTTATCAGCTAGAGGATAGTGATAATGGGGAACAAAGGAGGAGTAATAAGCAGTTGGCAACAGCTGTTTCTGAATCTGAGCCATCAAAGATGTTTGATGATGTGTTGCTATGCAACTGCGAGAACAAAGATGCTGTACAATGTCTAGTTCACGGAATCAAGTTGAATAAATCAGATGAAAAGGTGCAGCAGAATGAGCGACCAAAGTCGTCTGGTGGTAAGACAAGACGTGGGAAGAAGAAAGGTAAGAAAGGCGAACTGGTGGATTTGTGGACTCTGTTAACTGTTTGTGCACAAGCTGTGGCTAACTATGATCAAAGGACAGCAAATGATCTATTGCAGCAGATAAGGCAACACTCTTCAGCTTTTGGTGATGGAACACAAAGATTGGCGCATTACTTTGCTCATGGCCTTGAGGTGCGCCTGGCAGGGACTCAGACGCCAATTGCAATACATCTTAGTACTAGGGCATCGGCTGCTGATGTTCTACAAGCTTACAGAGTGTATATATCATCATGCCCTTTCAATAGGATGTCATTTTTCATGTCAAATAGAACAATTTTGAAACTATCAGAGACAGCAACAAGGCTCCATATTATTGATTTTGGCATTGGATATGGTTTCCAATGGCCTTGCTTTATTCACCGCGTCGCTCAGAGACCTGGCGGTCCTCCCAAGCTTCGAATTACAGGTATTGAGTTTCCTCAACCAGGGTTCCGGCCTGCAGAAAGGGTTGAAGAGACAGGGCATCGATTAAAAGCCTATTGTGATAGATTTAGGGTCCCATTTGAGTACACTGCCATAGCCAAGAAATGGCATACAATCCAACTAGAGGATCTCAAGATTGACAGCGAAGAGATGACAGTCGTTAGCTGTATGTACCGAATGAGGCACCTGCCTGATGACACAGTGGTTGTTAGCAGTCCAAGAGATACTGTCTTAAAACTGATCAAGAGTATAAATCCAGACATATTCATCCATGCAGTTGTTAATGGTACTCACAATGCACCTTTCTTTCTCCCACGATTCAGGGAGGCTTTATTCCATTTCTCTACATTGTTTGATATATTTGAGGCTACTTTATTTCGCGAAGATCAAGGGAGGATGGTGTTTGAGAGAGAAATATTTGGGAAAGACGCCATGAATGTTATCGCCTGTGAGGGTGTAGAGAGGGTTGAAAGGCCGGAGACATATAAGCAATGGCATGCAAGGAATCAGAGAATTGGGTTTAGGCAACTCCCACTAGATAGGGGTTTATATGAAATGGTCATGAATTTGGTGAAATCAAACTATCATCAAGATTTTATTATTGATGAAGATGGCCAGTGGCTGTTGCAGGGGTGGAAAGGAAGAATATGCTATGCTCTTTCTGTCTGGAAACCTGTCCAGGAACAATGA

>LcGRAS13

ATGGTTATGGATCAAGTTGTAGGAGGTTTATATGGTTCTGGCGATCAATACAAACTCAATGGTGAGAATCAGACCCTTTCGGTCGTGTTGAGTCAGAATCATGTTAATGGATTCAAAACCAGTGATAGTTTTGTTAATCAGAACTATTTAGATATGCAACTTTTGCCGTGTGATCCAATCCCCAATTATTCGACTCCAGTTTTGGTTGTGAGCGAAGAGGGAGATTCACATGAGGATTATGATTTTAGTGATGTTGTTCTGAAGTATATAAATCAGATGCTTATGGAGGAGGACATGGAAGAGAAGATCTGTATGTTTCAAGAGTCTTCAGTGGCGTTACAAGCTGCTGAGAAATCGTTTTATGAGCTTATTGGGGAGAAGTATCCTCCTTCACCTGATGGTGACTTGAACACTCGTATAGATAAAAACAATTGTGGACCGGATGGAAATGATGATATCAGTTGCACGCTCAGTGCCAGTAGTACTAGTTTGGTGGATCACGGGTGTAATAGTGATGTGAGTGATTGTAGGTCTTCGAATGCTGTTTCTCAGTCTACTTCTACTTCTCAGTCATCTTATGGCTCCGTAAACAGCACTGGCAATGTGGTTGATGGGTTTGTGGATTCTCCGGTGAGCACTCCTAGAATTCCTGACTTTTTTGGTGACAGTGAGTCTGCGAAGCAATTTAGAAGAGGGTTTGAAGAGGCAAGTAAGTTCCTGCCTAATGGTAGTGCTTGGTTTGGTGATCTGGATATCAGTGAATTGTTCATGAAAGAGTTTAAAGGGAAACAAAAGGATGTGGTTAATAAGGCGGGGGAGAAGCCAGAGAACGAGTGCTTTCTTGATGAGTCGAGGGGTAGGAAAAATCCTCTATCGCAGGATGCAAATTTAGGGAGTGAGAGAAGTAACAAGCTGTCAGCATTTTATACTGAATCAACTGTGAGCAAGGAAATGTTCGATATGGTGCTGTTAAATTGCGGACAAAGTGAATCTGCACTTCGTGAAGCTTTGAAGAATGAGACTAGCAAAAGTACTAAGGAAAAGAAACAATTAAAAGGATCCAATGGTGGAAAGGCCCGTGGAAAGAAACGAGGAGGTAAAAAGGTTGTGGTTGATTTGAGGACTCTCCTGTCCCTTTGTGCACAAGCTGTTGCAGCCAATGACCAGAGGAATGCACATGAGCTACTCAAGCAAATCAGACAGCATTCTTCTCCTACCGGGGATGGGATGCAAAGGATGGCCCAGTGTTTCGCTGATGGCCTTGAGGCACGCTTGGCTGGCTCTGGAACCCTGATTTATACTGCTCTTATATCCCAGCGTACGTCTGCTGCTGATGTCTTGAAAGCTTACCATCTTTTTCTTGCTGCATGTCCATTTAGAAAGCTCTCAAATTTCTTTTCAAACAAAACAATTATGAATTTAGCTGAGAAAGCAACAAGACTTCACATAGTAGATTTCGGTATCATGTATGGTTTCCAGTGGCCATGCCTCATACAACGTCTTTCGTCTAGGCCTGTTGGACCTCCTAAGCTTCGGATTACTGGGATTGATCTGCCACAACCAGGTTTTCGACCAGCAGAAAGGGTTGAGGAGACAGGAAGCCGCTTGGCGAACTATGCAGCAACATTTAAAGTTCCATTTGAATTCAATGCAATAGCACAGAAGTGGGACACCATTCGAATTGAGGATCTCAAAATCGACAGCGATGAGGTACTTGTTGTGAACTGTCTGTATCGCTTTAGATATCTGCTTGATGAGACTGTGGTGGTAGAGTCTCCAAGGAACATTGTTTTAAACCTGATTAGGGAGATGAATCCAGATGTTTTCATACAAGGGATTGTCAATGGGGCACACAGTGCACCATTCTTTATCACGAGATTCAGAGAGGCTCTCTTTTTTTACTCCACTTTGTTTGACATGCTGGAGACCAATGTGCCTCGAGAGATTCCAGAGAGGATGCTGATTGAGAGAGAGATATTTGGGCGGGAGGCAATGAATGTCATTGCTTGTGAGGGCGCAGAGAGGATCGAGAGACCAGAAACATACAAGCAATGGCAGGTCCGGAACATGAGGGCTGGGTTTACTCAGCTTCCGCTAAATGAAGAGATCATGAAGATGGCAAAGGAGAGGGTGGGTACAAATTACCACAAGGATTTTGTCATTGATGAAGATAGCCAATGGTTGCTTCAAGGCTGGAAGGGACGAATTGTTTATGCCCTCTCTTCATGGAAGCCTTCTTATTAA

>LcGRAS22

ATGCAAACATCCCAGAATCATCAAAGTTCTGCCAGTATCCATAGCTTGTACCACCAGCCTGTGCAAGGGATCGATCCCTATTGTTTGTCTCATTTCCAAATCATAGATGATAACATGTGCTCAGATGGTGGTAGCCAAGGAACCAGTGTATCCTTTCAGGCATACAAGGACCAGTTCTATACCCTCGAATCATCCACAGGAACTACCGGTTTTCTTATGTATGATTCCCCGTCTGTTGTCAGTAACTCGTCTAACCGGAGTCCCTTTTCACCACAAGGTTCTCATTCGTACCAATCTGATCCTCATCACTCTTCTGATAATACTTATGGATCACCTATGAGTGGACATTCCACTGTTGATGACGACAATGACACAAGGCTCAAACTGAAAGAATCGGACAGACTCAAATTGAAAGAACTGGAGAGATCGTTGCTGGGGCCCGATTCAGACATCATTGATAGCGGCAACTGCTGCTTCAAGGGTGTGGCTCACCAAGACACTTCTGCTGCACGTTGGAACTTGAATCAGTTGATGGAAATGAACCTGAGGTTGGACTTGAAACAGGTGCTTCTTTATTGCGCTCAAGCAGTATCTGAGAGCGATTTCTCATTAGCTGCTAGTCTGATGCATGTGATGGAGCAAAGGGTTTCGGTCTCTGGCGAGCCGATTCAAAGGTTGGGCGCATACATGTTGGAAGGGCTTAGAGCAAGATTGGAGTTTTCGGGGTATAAAATCTACAAAGCCTTAAACTGTGAACAACCAGTAAGCTCAGACCTGATGACTTACATGGGTATCCTTTATACCATCTGTCCATATTGGAAGTTTGCATACACGTCTGCAAACGTTGTCATAGCAGAAGCTGTTCAGAATGAGCCTAGGATTCACATCATTGATTTTCAGATTGCACAAGGCAGCCAGTATATCGAACTTATCCCAGTTCTAGCCAAACAGCCCGGTGGACCCCCGGTTGTTCGCATAACGGGGATTGATGATTCCCAATCATATCATGCTCGAGGTGGGGGACTTAGTCTTGTAGGACAGAAGCTGTCAAAGGTTGCCGCCTCATACAATGTCCCATTCGAGTTCCACAATGCTGCTATGTCTGGTTGTGAGGTTGAACGAGAACACCTTAGCATCCAGCCTGGGGAAGCCGTGGTTGTGAATTTCCCTTACATGCTGCATCACATGCCCGATGAGAGTGTAAGCATAAAAAATCACAGAGACCGCCTACTGAGACTTGTAAAGAGTTTGTCACCAAAAGTTGTCACCATTGTTGAGCAAGAATCCAAAACCAACACCAACCCCTTCTTCCAGAGGTTCCAAGAGACACTAGACTATTACACAGCCATGTTCGAATCAATAGATGCAGGCTCTTCAAGGGATGACAAGAAGAGAATCAGCGCCGAACAGAACTGTGTGGCTCGTGACATAGTCAACATGATAGCTTGTGAGGGCCCGGAGAGAGTAGAACGACATGAACTTCTCGGGAAATGGATGTCACGACTATCCATGGCTGGATTCGCCCAGTACCCTTTAGGTCCTTCCGTTACAAATGCCATTCTGGATCTATTAAAGGACTATGACAAGAACTATGCAGTTCAAGAGTATTGTGGTGCTCTCTATCTTGGCTGGAAGAACAGATGCATGGCAACCTCTTCTGCTTGGAGGGTGTCGAACCCTGCCCCCTTCAAAGGGAGGCAGGGCTGGCTGCTGGCGCTACCACTGCGCCTGCAGCCAGTTGACGATACAGTACAATAA

>LcGRAS14

ATGATTATTGAACCGGAATCGAATACTGGCGCTGATCACATATTAGATTGGCTTGAAGATTCGGTGTCATTCTTTCCATCGTTCTTGGATGATCCGTACAACCCAGGCGATCTCAACGGCTATCAATGGTGGGATACAGGTCCGGATACAAGCCAAGAACATGTGATTGGTGTTGGTTTTGGTGCTATTTCTTCTGTCAACAACGTAAACACAAGTATAGATACCAGTACTGCTAGTGTGAAACCTGTTGAACATTCTTGTGTTACTAGCGATCATCCACCAACGTCTTCGGATTCTTCAAAGAAACGTCGAAACACCGATGACAACCCAATTCCAAGATCATCACAGACGCATCATCAGAGGAAGAATCATGGCCGTCGGATCAGCGAAACGGAAGAGGGTGATGATCAAGAAGTGGTGACAGCTAAAAGGTCAGTTGGTAACAAGAGAAACACAAGCAAGTCTACAGGAAATAACAACAATAACGGTAACAACAAGGAAGGCAGATGGGCAGAGCAGCTGCTCAACCCTTGCGCGGCAGCCATCACAGCCGGTAACCTGACACGTGTCCAACACCTATTGTATGTCCTTCACGAGCTCGCCTCCCTTACTGGAGATGCGAACCACCGGCTAGCAGCTCACGGCCTCCGAGCCTTGACTCAACACTTGGCTTCTTCATCAACCTCCTCATCCACCTCATCAATTAGTCCTCCAACTTTTGCTTCGACAGAACCAAAATTCTTCCAAAAATCACTACTCAATTTCTATGACAAAAGTCCATGGTTTGCCTTCCCAAACAACATTGCAAACTCTTCTATCCTCCAAATTTTAGCTCAAGGAAAAGACCTTAATCGCGGTCTTCACATTCTCGATATAGGGGTATCTCATGGTGTGCAATGGCCTACACTTCTTGAGGCCTTGAGTAATAGAAGGTCAGGTGGCCCTCCACCTCTCGTACGCCTTACGATCATCGCTCCAACCTTCGAAAATGATCAAAACACAGAGAGCCCATTTTCGGTTGGTCCACCAGGAGACAACTACTCCCTACAACTCCTAAGTTTTGCCAAGACGATGAACATAAATCTCCAAATCACTAGGCTAGATAACCACCCTTTACAAAAACTTAGTGCACAAACGATCGACATCTCCCCAGAAGAGACCTTGATTGTTTGTGCACAATTCAGACTCCATCATCTGAATCACAACAACACCCCAAACGAAAGAACCGAATTCTTGAAAGTGTTGAGAGGCTTGGAGCCTAAAGGAGTCATACTAAGCGAAAACAACATGGACTGCAGCTGCAACAATTGTGGGGACTTTGCCATAGGCTTTTCGCGAAGTGTGGAGTACTTATGGAGGTTCTTGGACTCAACAAGCGCGGCATTCAAAGGGCGAGAGAGCGAAGAGAGGAGGATGATGGAGGGAGAGGCCGCGAAGGCGCTGACAAACAAGAGAGAAATGAATGAAGGCAAGGAGAAATGGTGCGAGAGAATGAGAGATGCAGGCTTCGTTGGAGAAGTTCTTGGAGAGGATGCCATAGATGGAGCTAGAGCTTTGTTGAGGAAGTATGATAGCAATTGGGAGATGAGAGTGGAAGAGAAAGACAAGTGTGTTGGACTGTGGTGGAAAGGGCAGCCCGTGTCTTTCTGTTCATTGTGGAAACTAGATGATGTCAAAGTGATTGATCAAAGTATTAGATCAACTTTGTAA

>LcGRAS30

ATGGAATATGAGCAAGTCCATTGCCTCATTGAACCAAGCTGCCTGGAAGAATCTCAGGATTATAGCTTGGAAGCCACATTTTCTTTACAAGTAGAAGATTACATCTCCCCAAACACTACCACTGTAGATGACATCCTGAGCAATGATCAAAATCTGGAAAGATTGCTCCAGATAGAGACAGACCTCAGCGAATTTGATTCCCTCAATCAAGATACAGAGATGGGTCCTGAAATATGCAAAGAAAGTACTGAACTGGGTCATGAAATGATTCAAGAAGCACAGGAGAAAGTACATGCATTAGAAGAAGGAAATGATTCTCCATTGAAGGGAATCCAAGAAGAATTAATGGAGGACAGCTGTGTAACCGACCTTCTATTGATGGGAGCTGAAGCTGTTGAAGCACAAAACTGGTCTCTCTCTTTAAGCATCATCACAAAGCTCAGAAGTCTCTTGATTGATGGAGAAAATGGCGATAACCTGTTCAATAGATTGGCTTTGTTCTTCACTCAAGGCCTACTATATAAGGTCAATGATGTTCCTGGAATCATGCAGGATCCTGTTTCTAAGCAGGGTAATAATGCTATGTCTTCCTTCCAGATTCTTCAAGAACTTTCCCCTTGTGTAAAATTTGCACATTTTACGGCCAACCAAGCAATCATAGAGGCCACACAGGGTGATTCAGAGATTCATGTCATTGATTTTGACATCATGGATGGAATTCAATGGCCCCCATTGATGGTTGATCTTGCAATGAGGAAAGATGTTTCTCTCAAAGTGACAGCCATCATCACTGACCCTCAAAATCAAGTTTTTGTTCATCAGACTGGAAGAAGACTAAAAGAATTTGCAGACTCAATTGATTTTTGTTTCGAATTTGATTGGATGACAATGGAGAAAGAAGAAGATTTTGAAGGGATCAAAGTTGGTGAAACTCTGATAGCCAACTGTATGATCCATCAGCTTCACGTGCCTAAAACAAGTTTCTCATTAGTGAAATCCTTCTTGGGTGGTATGGCCAAACTATCACCTAAAATGGTTGTTTTAGTAGAGGAAGACTTATTTAGTTTCACAAAACTCCCATCAATGTCCTTTGTAGATTTCTTCTGTGAGGCCCTCCACTATTACACTGCACTTTCTGATTCTCTTCTAAGCAGTTTTTCCACAAAGTACAAAATCAGATTGAGGCAAATAGAGGAGTTTTTGGGGGTTAGAATTTTAGATTGTCTGAGGCAATTTCCTTGTGAAAAGAAAGAGAGAATGTCTTGGGGAGATGGTTTTGCTTCCTTGAGAGAATTCAAACCAAAAACTTTGAGTTCTTGGAACATCTCTCAAGCCAATTTCTTGGTTACTCTTTTTAGTGGGGGGTATTTTGTGCAACATGAAAAATGCAAGCTGGTCTTGTGCTGGAAGTCAAGGCCCTTGACTACCGCATCCATTTGGGTGCCAATATCAAAATCAAGCTTAAATAGGATGCAAACGTGCTCTACCTTATAG

>LcGRAS17

ATGGAAACCATGAATGGTTGGCTCTTCTCTCCCATCAATGAGAGCTTCAATCATGACCTGGCCATCAGAAGGTTTTGTCCTGCAAGAATTGAGCAAGAGCAGAAGGAGTGGGAGGAGACAATGAACTACGGTGAAAAAAATTTGTTTTCTCCTTTTGACATTGATGCAATTAGCACTCCTGACTTGGCATCCAATTCTGAGGTGGATTATGAGTTTGTTGATAGCTTCATCAACATGGATGATGATCATGATGAGAAAGTTGAAGACCCCAACTCATCTGAGAAGCACAAGATGTTAGAAAATGATGATCAACATCAGATTAATGAGACTACCTTCTCAATTGGAGTGGATGATGTTTATGATAATAACATTAATGGAGATCATGATCAAGTTCCAATCATGACAGAAAAAGGAGCTCATATTCATGAGGATGACCAAGTTGGTGTGGGCACTGAAATGGTGGTACCTAGAATGGAGGAGGTGAATCATGGTGTGGACCAAGGGCTTCACTTGGTTCACCTTTTGTTAGCCTGTGCTGAGGCTGTGGGGTGTAGAGACACACAACTGGCAGATTCAATGCTCAGCCGAATTTGGGCCTCAGCCAATCCATGGGGAGACTCATTGCAAAGAGTGTCTTATTGCTTTGCATTGGGGTTGAAGTCCAGGCTCTCACTTCTCCACAACAACATCAATGCAAACGGTACATTCTCTAATGGAGCTACAAATGTGTCATTGATCAATAGAGCGGAGAAGATGGAAGCTTTTCATCTTTTGTACCAAACAACTCCTTACATTGCTTTTGGTTTCATGGCTGCAAATGAAGCTATACTTCAAGCAAGTCAAGGTAAGGACTCTTTGCACATAATTGATTTTGGTATGGAGCATACACTCCAATGGCCTTCTTTGATTAGAAGTCTAGCTTCAAGGCCTGAAGGCCCTCCAAAAATCCGAATTTCGGGACTAATTCATGATCATAACATGTTGGAGCTTGAGAGCAGCAAGAAAGCACTTGTTCGGGATGCAATTTCACTAGGAATTGCATTGGAGTTCACATTGATATTAGAGCCTGTCACAACCTCACTTTTGACTGAAGAGAAGCTGAAATTGAGAGAAGGTGAGACATTGTTTGTTAATAGTATTATGAATTTGCACAAGTATGTCAAAGAGAGTAGAGGCTCTCTCAAGGCAATTCTCCAAGCAATCAAGAAACTCAATCCAACATTGGTCACAATGGTGGAACAAGATGCCAACCACAATGGTCCATTTTTCCTTGGTAGATTTCTTGAATCTCTCCACTACTACTCTGCCATTTTTGACTCCCTTGAAGCTTGCCTCTCAAGGAACAGCCCTCAAAGGATGAACATTGAGAGGCTACACTTCGCAGAGGAGATACGCAACATTGTGGCCTACGAGGGGTCTGATAGGATTGAAAGGCACGAAAGAGCAGATCAATGGAGAAGGCAATTTGGGAGAGCAGGATTTCAAGTCGTGGGGTTGAAGTGTTTAAGCCAAGCTAGAATGATGTTGTCTGTTTATGGGTGTGATGGTTACACTTTGGGCAGTGAAAAGGGGTGTCTTTTGCTTGGATGGAAAGGCAGGCCTATAATGCTTGCATCTGCTTGGCAAGTGCACAATGTTTCTTCTTGTTGA

>LcGRAS4

ATGCGGTGTTACTGGTGCGGGCTTTACCTACTCATCTTATTCATCCGTCCCTTCGAACCTAACAGGTTGCTTGGATCACTAAAATTTGATATAGGAAACTCACCCAACTCGCCCTTTCTGACTCCAAGTGATTGTGATACTGTTACAACATTGAGTGACAGCCAGGAGCAGCATAGCTCA

ACTGAGAATATTTCTGGTCTCAGCCCTTCTTGTAATTCTTCGCTGGAATCCAACACTTATTTTCATCAGTTTAGTCCTCCCGCAGATAGTCTAGTTCTGTGTTCTGGTGGGAATACGTATCCACAGAATGCAAATTGTAGCCCAATTGTGAAACACGTTTTGCAGGAATTGGAGACTGTG

CTTATGGAGCCTGATGAAGATGAAGATAAAACTGCCCTGATTAGTTCTTCAGCACATACTAGGGTGCAGGCATCGGGCCAGAGATCTAGAGCATGGGGCCAGGAATGCCAGGGTGCAAGTGTGATTCAACCGAGTCCTTCCTCTAGTTCTAGGTACAGGCAGTCAGGTGAAGGTGCTCAA

ATTGAGAACCGTCAGAGGTCAATGGAGGAACTATCTCTGCAGGGTTCTCCTTCAGGCAATTTGAAGCAGTTATTGATTGCATGTGCTAAAGCTTTGTCCGAAAACAACCATAGTGATTTTGATAATCTGATTGAAAAAGCTAGAGGTGCTGTGTCTATTGCTGGAGAACCCATCCAGCGT

CTTGGTGCTTACTTGGTAGAAGGTTTAGTTGCCAGGAAGGAGAAATCGGGCTCCAATATATACCGTGCCCTTCGCTGCAGGGAGCCTGAAGGGAAGGACTTACTGTCTTACATGCACATCTTGTATGAAATCTGCCCCTACTTGAAGTTTGGTTACATGGCAGCCAACGGGGCCATTGCT

GAAGCATGCAGAAATGAGGATCGCATCCATATTGTCGATTTCCAAATAGCTCAGGGCGCACAGTGGATGACTCTCTTACAGGCTCTTGCAGCTAAACCATCTGGGGCACCGCATGTGCGAATCACAGGAATTGATGATCCCGTCAGTAAATATGCCCGTGGTGATGGATTGGATGCGGTT

GGGAGACGTTTGGCGGATATCTCTGAGAAATTCAATATCCCTCTTGAGTTTCATCCTGTGCCAGTTTTTGCTCCAGATGTGACGCTGGAAATGCTTGATGTCAGGCCAGGGGAGGCTCTTGCAGTCAACTTCCCTCTGCAACTCCACCACACCCCTGATGAAAGTGTTGATGTTAACAAT

CCAAGAGATGGGCTCTTGAGGATGATAAAGTCACTAAATCCTAAGGTGGTCACTTTGGTGGAGCAAGAATCGAACACAAACACTGCTGCGTTTCTCCGGAGGTTCACTGAAACTCTGAACTACTACTTAGCAATGTTCGAGTCAATTGATGTGACCATGCCAAGAGACCATAAGGAACGA

ATTAACGTGGAACAGCATTGCTTGGCACGAGACATTGTGAATGTTGTTGCTTGTGAGGGGAAGGAAAGGGTGGAGCGACATGAGCTCTTTAGCAAGTGGAAGTCTAGGTTCATGATGGCAGGCTTCCGCCAGTGCTCGTTAAGTAATTACGTCAACTCTGTCATTAAGAGCTTGCTGAAG

TGTTACTCAGAATTTTATACGCTGGTGGAGAAGGATGGAGCTATGTTGTTGGGCTGGAAAGATAGAGACCTTGTATCTGCTTCTGCATGGCACTGA

>LcGRAS2

ATGGACATAACTCTTTTTTCTCCAAAAGGAAATCTCCCAACTCACTTCTTTCCTTCCAACCAGCAAAGCAATGGTCAGATATCAATAGATATGCAGCATCAAAGCAATCACCAGCCACAAAACAGCCACACATCGACGAGCCGGTCATCGGATTCCGGCGAGCCATGTGCCACAGCCAACAAATGGGCATCAAAACTTCTCAAGGAATGTGCTAGAGCCATCTCCGACAAGGACTCTAGCAAAATCCATCACCTTCTTTGGATGCTAAACGAGCTCGCTTCACCTTATGGAGATTGTGATCAGAAACTGGCCTCTTACTTCTTGCAAGCTCTCTTTTGCAAGGCCACAGAATCAGGGCACAGATGCTACAAAACCCTAACTTCGGTTGCTGAGAAGAGCCACTCCTTTGATTCAGCCAGGAAGTTGATCCTAAAGTTCCAAGAGGTGAGTCCATGGACTACTTTTGGTCATGTAGCTTCCAACGGTGCAATCTTGGAGGCCTTAGACGGAGAAACCAAACTTCATATAATTGATGTAAGCAACACCCTTTGCACTCAATGGCCTACTTTGTTGGAAGCTTTGGCTACAAGAAGTGATGAGACTCCACACTTGAAGCTGACTGTTGTTGTGACTGCCAACATAGTCAGATCGGTCATGAAGGAAATAGGTCAAAGAATGGAGAAGTTCGCTAGGTTGATGGGTGTTCCCTTTGAGTTCAATGTGATAAGTGGGCTAAATCGATTAGGAGAGCTCACCAAGGAAGGACTAGGTGTTCAAGAAGATGAAGCAATTGCTGTGAATTGTATTGGAGCCTTGAGAAGAGTTTCAGAAGAGGAAAGAAGGACTGTGATTCAGATGTTTCAATCACTCGGGCCAAAAGTTGTGACGGTTGTCGAGGAAGAAGCTGATCTTACAAGCTCTAGTTATGACTTTGTCAAGTGCTTTGAAGAGTGCCTTAGATTCTACACATTGTACTTTGAGATGCTAGAGGAGAGCTTTGTCCCAACAAGCAATGAAAGGTTGATGTTGGAAAGGGAGTGCTCCAGGAGCATAGTTAGGGTTTTGGCTTGCGGTGATGAAGATGGTAGTGGAGAAGAGTGTGATAGAAGGGAAAGAGGAAGCCAATGGTCAGAGAGGCTCAGGGAGGCATTTTCACCAGTTGGGTTCAGTGATGATGTTGTTGATGATGTCAAAGCATTGCTAAGGAGGTATAGAGGTGGGTGGGGACTTGTACTCCCACAAGCAGATCACGACTCCTCAGGAATTTACTTAACATGGAAAGAGGAACCAGTGGTGTGGGCTTCAGCTTGGAAACCCTAA

>LcGRAS42

ATGATGCAAACAGACGTTTTCCTTCCATCTTGGACACTCTACAAGGACACGAATCCATCTCCTGATGATCAAGTTTCCATCTATGGTGATTTCAACGTTGATGCTTATGTGGGTGGTTGTGATTTCTCAGCTCCATTTACCATGTCAAATGATTTTTCAAAAATCCCCTCCATCCCCAACTTTGCCTCCTTGTTTTCCAATGAACTCCTCCAGTTTCCAGCTTCTGATGACCAGCTTCAAGCCATGTTACCAGTGGAGGATTTTGATATGGGGTTGGTTGGATTGGAGTCAGTTTTGAGTGACAAAGTTTTTGAAGACAGTGATGCTTGGAGTCCAAGTCAGTCAATGAAATCCAGTGAAGGTTCTATGGACACTGCACTGACACTTCCAGGAGAAGCCATGGAGATAGACAATCAACTTGGTGTTTTCCATCTTCTCAAGGCCTATGGAGAGGCCATGGAGAGGGACCAGAGGGAGCTTGCGGAGGTGATCATGAGATGCCTGAGTGAGAAAGTGAGTCCAGCTGGTGAGACCTTAGAGAGAATTGCATTCAGTTTGTCACCTGATGTTGAAAAACAAGGGGACTATCTATTGCAAGAGTCTAGCAAGAACTATGAGGTAGCTTTCAGGACGTTTTACCAGAGGTTTCCTTATGGTAGATTCGCTCATTTCGCGGCGAATTCAGCCATCATTGAGGCCATGCCTGATGATGTTGAGATTGTACATGTAGTGGATTTCAACTTGGGAGAAGGGGTTCAGTGGCCTTCCATGATTGAGGCCATTGCAAGGAAGAATAAAATGCTAAGATTGACAGCAATGAAGAGGGAGGATGAGGACTTTGCTTGTGCCTCTGTGTCATCCAGTTTCGAGGAGACCAGTAGGCGACTAAGCGATTACGCGGCATCATTTGGATTGAAGTTGAAGGTGAAGGAGATGGGAATTGAGGATTTGGTGAGGGAGATCAAGAAAACAAAGAAAAGAGGGGGCCGTCGTGGTGGTGGTCGTCGTAGTGAAGAGTGGTTGGTTTTCAATTGTATGGTGGGGCTTCCACACATGGGGAGGGTGAGAAGCAGGAAGCTTGTGAAGGAGTTTCTGCAAGTAGCCAAGGAATTGTTATCATGCACAGGAGGCAATTTTACAAAGAACAAAGGGGTTGTAACTTTAGGTGATGGAGATGCTTGTGAGAAGCTGACAGATTGTTCCGGATTCGGGTCATTTTTTGATGGGTATTTGGAGCATTATCAAGCTCTTCTAGAATCAATAAAGTACAATTTTCCTGTACATCTAGCAGAGGCAAGGATGGCACTGGAGACTCTCTTTGTAAAACCTTTCGTCTCTTCCGAAGTTTGGTTCCAAAAATGGGAGGAGGTAAGGCAAGGTTACCATCTCCAAGGAGTTGGGTTGAAGGAAAGTCAAGTTAGCAGAGAAATTTTGATGGAAGCCAGAGAAATAGTGAACGTGGAGAGTTCATTTGGAGTGACACTAGGAGGACAAAGTGAGAACGAGATGGTCCTTGAATGGAGAGGAACACCGTTGGTCAGAGTGTCTAGTTGGAGAAACTAA

>LcGRAS48

ATGGCTGCTTGCGGTTTGCTGGGTGTTAAAGGGCAAGATTCACTTGTTAATGGCAGTAATATTATCGGGAGTGAAAGCCCAACAATGACAAGCGCCTCTAATAGCACGGGAAGCAACGAACGTCGGCCCTGCGTGGACGGGAAGATGATAAGAAAGAGGATGGCTTCTGAGATGGAGGTGCAGTTGAATGGTGATTATAATTACAATAGATTGCCAAGAAGGAATAACCCAATTTCAACCCCGACCGCCATCCCGGTCCTCAACTACTCCACTATCAATAATATCAATATGTTACCTTCTTCCACGAACTTGACCAACATGACGTCAGGCGGGTCTGGATTTTTATCTACCGCTACATCAGCAACAACAACAACAACAACAGCAACGAGCATCATTGATGTGTCCTCTAATCCTCCACAAACCCAACCCCAACCCCAACCTCCTGCTGTTTGTGGCTTCTCGGGTCTTCCTTTGTTCCCACCAGACCGAAATCGGAACAGCTCCATCAGCCTCATCCCGGCCACCACTAATATTAGTACTGTTGTAACTTCGCCGCCTTCCATGGAAGATAGCTCAGCCACGGCGTGGATCGACGGCATAATAAAGGACCTCATCCATAACTCCACCAACGTCTCCATTCCTCAGCTTATCCACAACGTTAGAGAGATCATCTATCCTTGCAACCCCAATCTTGCTGCGCTTCTTGAGTACAGGCTCCGCTCTCTTACTGAGCCATTAGAGAGAAGAAAAGAGACGTTACCCGTGCATCTACTCCAAAGGCAATACAACGTTCAACATGCCTCTTCTGGGCTTACGCTCAATCTGGAATCTGGGCTTGACAACGTCCCTAATTACTCGCTACAGGATTCCCCTTATGTGAACTGGGGACTGACGCAACTGCCCAACCCCACCGGCCAAGTTCACGACCACCGTCAGTCTGTTACATCAGCTCATCAGGTGCAACCTCAGCCTCCCCCTCAAGAACAACAACAGCAAGAAGAACAAGAACACTCCTCGCCCGTAGAGACAAGTACGACGCCAACAACAACAGCACCAACACCAACATCTTCTGCGAGCATCAGAGAGAGAAAAGAAGAGATGCGGCAGCAGAAGAGAGACGAGGAAGGCTTGCACCTGCTGACCTTGCTCTTGCAGTGCGCGGAGGCGGTGTCCGCCGACAACTTTGAGGAAGCAAACAAGATGCTGCTGGAGATTTCTGAACTGTCGACGCCATATGGTACTTCTGCACAGCGTGTGGCCGCGTACTTCTCGGAAGCAATGTCAGCAAGGCTGGTGAGCTCATGTCTTGGAATATATGCAGCGTTGCCTTCGCTGCCCCAAAGTCATACCCAGAAGATGGTATCGGCCTTCCAAGTGTTCAATGGTATAAGCCCCTTCGTCAAGTTCTCTCATTTCACGGCCAATCAGGCCATACAGGAAGCTTTCGAAAGGGAGGAGAGGGTGCACATCATAGATCTAGACATCATGCAAGGCCTTCAATGGCCTGGACTCTTTCATATCCTTGCTTCTAGACCAGGGGGACCTCCTTACGTACGCCTGACTGGGCTTGGCACCTCCATGGAAGCTCTCGAGGCTACCGGCAAGCGTCTCTCTGATTTTGCCGAGAAGCTCGGCCTCCCCTTCGAGTTTTATCCGGTTGCAGAGAAAGTTGGGAACTTGGATCCAGAGAGGCTCAACGTCAGCAAGAGGGAAGCCGTCGCTGTCCACTGGTTGCAGCATTCCCTCTATGACGTTACTGGTTCTGATACCAATACCCTCTGGCTTTTGCAGAGATTAGCACCGAAAGTGGTGACGGTAGTGGAGCAGGACCTGAGCCCGGCAGGTTCATTCTTGGGAAGGTTTGTAGAGGCCATACACTACTACTCAGCGCTATTCGACTCCCTCGGAGCAAGCTATGGGGAGGAGAGCGAAGAGAGGCACGTGGTGGAGCAGCAGTTGCTATCCAGAGAGATACGGAACGTGCTTGCCGTGGGAGGCCCCTCCAGAAGTGGGGAGGTGAAGTTTCACAACTGGAGGGACAAGTTGCAACAGTCAGGTTTCAAGGGTATCTCTCTCTCAGGAAATGCTGCTACCCAAGCTACCCTGCTCCTTGGTATGTTCCCTTCCGATGGTTACACATTGGTTGAGGACAATGGCACCCTCAAGCTAGGCTGGAAAGACCTTTGCTTACTCACTGCTTCTGCTTGGAGGCCTTTCCATACTATTGCTGTTTCCACTACACATCGTTATACCCACTAG

>LcGRAS31

ATGGGTTCTCTTAAGAGTGAGAACTCAAGCACCAAGTTGCTATCAACTTCTCCAAACGAGTCAGGCTGCTCGGAGATCTTAAAGAAGACGGTTTCTTCTTCTGACTTGGAGCAAAACAGCTTGACCCCACCAAGCCTCAACTTCCCACCCGTCAAATTCGAGTTGGATGGAGACGTCGAAGTGCAGTCACCGGACAGTGCGCTATGGGAGACGTTTTTCTCGGATCACTTGGATGGCGGTGACTTCATGGTGTCGTCTCCTGTGAGGAACTCGCCTTCTCGACAGACTTCAACTTACAACTACAATTACAACTTCGGTCAAGCAATGCAAGGCCAGAGTAGTCTTTCAGGGTGTTCTCCTCCGCGTTTCTCGGCGCAGCTGGGGGCTTTTAGTACCTCTCTCAGAGCCAAAGGGCAGAGCCCACTTCACAAGGTGTTTAAATCTCCAAACAACCAATACATGCAACCTGTAGAGAACCTTGCTTTGCCGGCTATCGAAGACTTCTTGGATGACTATCAAAAAGATGGATATGGGGTGTTCTCCCCGATGAGGATATCAAGTAGTAGTTCGTCCCAGCTGTTTGAAAGTCCTAGTTCGGTGCCAGCAATATTGGACTGCTTGACATTGCCGAGTTCTTCAAGGTTCTCTGGACCAGTTAGTGAATCATCATCAACAGCTGGTGGTTCTCAACTGACCCAAGAGAATGATATTTATCAATTAGGCACCATTAATGCAACTGCACCATTATCTGAACAACTCCAACAAGAGCGCCACCACGACAAGCAACAACAGCCTACACAAAACCAAACGCAAAGTATTAACCATAGCTTGATGGTCCCTCTTCCAATTAGCTCCGAACAGGAACAAGACAGTGGACTTCAACTGGTGCATCTCCTACTAGCCTGTGCAGAGGCAGTGGCCAAAGAGGACTACATGTTGGCTAGAAGATACCTTCACCATCTAAACCGTGTGGTGTCACCTTTAGGAGACTCCATGCAACGTGTCGCCTCCTGCTTCACCGAAGCGCTTAGTGCTAGACTCGCAGCCACCCTGACCACAAAACCAACCACTTCTTCACCAAAAACCTTCTCCCCTTTCCCACCAAACTCCCTAGAAGTCCTCAAAATCTACCAAATCGTTTACCAGGCCTGTCCATACGTGAAATTCGCTCATTTCACTGCCAACCAAGCCATCTTCGAAGCTTTTGAAGCAGAAGAACGTGTTCATGTCATAGACCTTGATATTCTCCAAGGATACCAATGGCCAGCTTTCATGCAAGCCCTAGCTGCCAGACCTGGGGGAGCTCCGTTTCTCCGAATAACCGGAGTCGGACCTTCCATCGAGTCAGTCAGGGAAACGGGTAGGTGCTTGACCGAACTCGCTCACTCCCTTCATGTTCCATTCGAGTTCCATCCAGTCGGTGAGGAACTCGAAGACCTGAAACCCCACATGTTCAACAGACGGGTCGGCGAGGCTCTAGCCGTCAACTCTGTTAACCGGCTCCACCGTGTCCCCGGTAACTGCCTAGGCAACCTACTGGCAATGATCCGTGACCAGGCACCAAATATAGTAACCCTGGTGGAACAAGAAGCCAGCCACAATGGACCCTACTTCTTAGGCCGGTTCCTGGAAGCACTGCACTATTATTCAGCGATTTTTGACTCACTAGACGCAACGTTTCCACCAGATTCAGCCCAGAGAGCCAAAGTAGAGCAGTACATCTTTGCACCAGAGATCAGAAACATAGTGGCGTGTGAGGGAGCGGAGAGAATAGAGAGACACGAGAGGTTAGAGAAGTGGAGAAAAATAATGGAAGGGAAAGGATTCAAGGGGGTTCCTTTGAGTGCAAATGCAGTGACACAGTCAAAAATATTGCTGGGTTTGTATTCGTGTGATGGGTATCGATTGACAGAAGATAATGGTTGTTTGCTCTTAGGGTGGCAAGATAGAGCCATACTTGCTGCATCTGCATGGCGATGCTGA

>LcGRAS23

ATGACTCAAGGATTCTTGTCTACTGGTAAGCTTTATAGTAATCAAGTGAGTAATGATGTGATTGGCCTTGAGGCTGGCCGTTCAACTTTGGATTGCTATCCTTCCATGCCAATGTTGGCGAATGCTGCTTCGAGCTGGATCCTCTCACGTCTGGAGGAGACTAGAGACCCAAAGAGGATTAAACGAACTATGAGCATTGCTGAATCCATGGCAAGTACTGACAATAGTCAATGCAGTAGTATTGGAAGCTGTATCAGCCGATGTAGCAGCACTAGTAGCTTGACTAGCACGCGAAGGCTGCAGTTCAGGGATCATATATGGACTTATACTCAAAGATATCTTGCAGCTGAAGCTGTAGAGGAGGCGGCAGCTGCCTTGATCAATGCAGAGGAGGGTGGAAGTACTGAAGAAGATGGAACCGCGGATGGAATGAGGCTTGTTCAGCTTCTCATTGCTTGTGCTGAAGCTGTGGCTTGTAGAGACAAGCCACATGCCTCAGCTTTGTTATCAGAACTTCGAGCCAATGCCTTAGTCTTCGGTTCTTCCTTCCAACGCGTTGCTTCTTGTTTTGTCGAAGGTCTTACGGAGCGTCTTGCAATGGTTCAACCCCTTGGGGCGGTTGGTTCAATAGCACCTACAATGAATATAATAGACATTGCCTCTGAAAAGAAGGAAGAAGCTTTGCAGCTTGTCTATGAATTTTGCCCACATATACAATTTGGTCACTTTATTGCCAACTTGGCAATATTGGAAGCCTTTGAGGGAGAGAGTTATGTCCACATTGTGGACTTAGGCATGACCCTTGGTCTGCCGCATGGTCACCAGTGGCGCCAGCTTATCCAAAGCCTAGCAAACCGTTCTGGCCAACCGCCCAAACGCCTTCGAATCACTGCTGTTGGCCTTTCTGTTAAGAAGTTCCGAATCATTGGTGATGAACTTAATGCCTATGCAAAAGATCATGGAATCAATTTGGAGTTCTTGGTAGTGGAAAACAACCTGGAAAACCTGAGGACTAAAGACATTAAGGTTTCTGAAAATGAAGTTCTTGTTGTCAATAGCATTCTTCAGTTGCATTGTGTGGTTAAAGAAAGTCGAGGAGCTCTAAATTCTGTTCTCCAGATAATCCATGAGCTCTCACCTAAAGTTTTGGTCCTGGTCGAGCAAGACTCAAGCCATAATGGTCCATTCTTTCTTGGCAGATTCATGGAAGCTCTTCACTATTATTCAGCCGTTTTTGACTCCCTAGATGCCATGTTGCCCAAGTATGACACAAAGCGCGCCAAGATGGAACAGTTCTACTTTGCAGAGGAGATAAAGAACATTGTGAGTTTTGAGGGGCCTTTAAGGGTTGAAAGGCACGAGAGGGTGGACCAGTGGCGGAGGAGGATGAGCCGTGCGGGGTTTCAGGCTGCACCTATCAAGTTGCTGACTCAGTCCCAGAAATGGCTCAAGAAAAATAATGTTTGTGAGGATTACACTGTTGTGGAAGAAAAGGGGTGCCTGGTTCTTGGTTGGAAATCCAAGCCAATTATTGCTACTTCCTGTTGGAAATGCTAA

>LcGRAS24 ATGAGAAGAAGCCAAAGTCCACCCACTTCAGCTTCCACTCTCTCCTCCTCTTTCAACAACAACACGCCGAAAATCCCGCAAGAATCGGTGGCTTCTTTGGTTAATAACGAAAGAAAAGATGAGTGGGCCAGTGAGTTGCAAGCGATTCCAGGTGGGTTGGAGTTGATTGGTACAGGGGGTGAAAGATGCGGTTTTGGGGCAGAAGATTGGGAGAGCATGTTGTCAGAATCGAGTCAAGAAGAGTCGATCATGAGGTGGATAGCTGGGGATGTGGAGGACACACCATTTGGTCTAAAACAGCTGCTGCAAAGTGGTGGTGGTGACCCTCTTGTTGATTTTGATGCCAATGCAGCTGGCTTCAGCTTTGTTGATCAAGCTCCGAATTTCCAGCAATTTGGTGGTGATGCAATTTCTACTGCTGTTAACAATTTAGGTGCTTTTTCTGGTTCTGTGTTTGTGTCAAATTACAACAATGGTTATGGAAGGCAAAATGCGATTTTCAGTCCTCAGAACATGCCTGTGGGTAGCGGGATTTACCATGAGTCTCGAAACTTTGTTGGAGCCCCAGAAGAGAAACCACAGATTGTTAACCCACAGATATTGATGAACCAACAACAGCAACACCATCAAAATGCAAGCTTCTTGTTGCCGATAACGTATCCACATCAAAAACAAGAAAACCACCACCAGCAGCCACAACCGAAGCGCCATAACTCTGGAGGAAGTTTCGAGCTTTCCTCTCATAAACCACCGTTGTCTGATCCAGCGCATGAGTTATTGCTGAGAAATCAACAACAACAACAGCAAATGGGGTTTTCTCAGAATGTGGAGTTTCTTCCTCATCATCTTCAAAAGCCTTTGATGGTAACTGCAAAACAGAAGTTGTGTGTATCTGCTCCAGGGAGCGAAGAGATGGCTCATCAACATCACGCTTTGCTCGACCAGCTCTACAAAGCAGCAGAGCTGGTAGGGACTGGGAATTTCTCAAACGCGCAAGGGATATTGGCGCGGCTCAATCACCAGCTCTCTCCAGTGGGGAAGCCCCTTCAAAGGGCTGCTTTCTACTTCAAAGAGGCTCTGCAGTTGATACTGCTTATGAACAACCCAGTCACCAACCCGCCACCGCGTAGTCCTACCCCTTTTGATGTTATCTTCAAGATGGGAGCTTATAAGGTGTTTTCTGAAGTCTCTCCTGTCATTCAATTTGTCAATTTTACTTGCAACCAAGCTCTTCTAGAGGCCCTTGATGATGCTGATAGAATCCACATTGTGGATTTTGATATTAGCTTTGGTGCTCAGTGGGCTTCATTTATGCAGGAGCTTCCAATGAGGAGTAATAGGGTTGCTCCTTCTTTGAAAATCACTGCCTTTGCCTCTCCTTCTAGTCACCATCCTATTGAGCTTGCACTTATGCGTGAAAATTTGGCACAATTTGCTAATGAGGTTGGTGTAAGTTTCGAGCTTGATGTTGTTAACTTTGATTCATTGGATCAAACCTCCTATATGCTACCCATTTTTCGATCGAATGAAAATGAGGCTGTTGCAGTGAATTTCCCTGTTTGGTCTTTTGCAAATCAACCTTCTGCATTGCGTTCTCTCCTTCGCTCTGTGAAGCAACTTTCGCCTAAAATTGTGGTGTCATTGGATAGAGGATGTGCTAGAAGTGACCTTCCATTTGCAGAACATATTCTTCATGCCCTCCAATCATATATAAACCTATTAGAATCCCTTGATGCTGTTAATGCTACACCAGATGCTGTGAACAAGATTGAGAGGTTCCTTCTTCAGCCTAGGATCGAAAGCACTGTATTGGGGAGGGTTCAAGCCCCAGAAAAAATGCCCCATTGGAAAACTCTCTTTGCCTCTGCTGGGTTCTCCCCTGTAACGTTTAGTAATTTCACAGAAACTCAGGCAGAGTGTGTGGTGAAGAGGACTCCAGTGAGGGAGTTTCATGTTGAGAAGCATCAGGCATCGCTCGTGCTTTGTTGGCAGAGGCGGGAGCTTATCTCAGCTTCTGCTTGGAGGTGCTGA

>LcGRAS39

ATGATGCAAGCTGAGCTTCTTCAGTTGTGGCCTGATTACTGCTCTGTGGATTCAACCATGGAGGAGGCAGGTCTTTATTTTGGCGAGAAAGATGCCCAATTGGGCAGCTTTGAGTTCTGTTCTCTGACGGAGAAGTTCTCGTATCCTGATGATTCGCAAGGTCTAGTTGTTTATGGTGAAAACGAATCGCCTGTTGTGTTCCCTACGGAGCAGTGTTTGCCTAGCTTGGAGAAGGCTGAGGCTCCTTTGAGTGGTCAGTTTACGGTTGATGAACTCAAGGGTCTTTGTGAGTGGATGGTGAGTCAGGAGGATGCTAAGAACCTGAATGTGTCACCAGTGTCAAGTGATGCATCTATTTGCGTCGAGTCAGGTTCAGCATCCCTGGTTTTGCCCTGTAAAGATATGGAGATTGATGACCATTTGAGCATTCTCCATCTGTTGAAGGCGTATGGAGAGGCCATGGAGCTGGAACAGGAAGAGCTAGCACGAGAAGTTATAAGGCGATTGAAAGAGAAGGCAAGTCCAATTGGTGCTACCCTTGAACGTGTCTCACATCATTTGATCCAAGCCTTGGAGAAGCAAGTAGACTACCTGAGTACACAATCGAGCAAGAATTATGAGGCAGCCTTCAAAGCCTTCTACCAGATATTCCCCTACGGGAGATTCGCTCACTTTGCCGCTAATTCAGCAATACTAGAGGCCATTCCTGTGGATGCTCGGATAGTTCACATCGTGGACTTTGACATTGGCAATGGCATACAATGGCCTCCTTTGATAGAAGAGCTTGCAAGAAGAGGCAATGTTTTGGTTAGACTGATTTTGATAAGATGGAAGGAAGAGGGCTGCAGCTGCTGCTGTCCTTTATTGGACAGTTTTACGCAGACGAAAAAGAGACTATATGAACATGCACGAATTTCTGCTCTGAGATTGAAAATAGAAGAGATGGACATGGAGGGCTTGGTGAGTGAAATGAAAAAGATAAAGAGAAGAGGTGGGAGACATGAATGGTTATCCTTCAATTGCATGGTGGATCTCCCCCACATGGGAAAAATAAGGAGTGTTAGGCACGTTATAGAATTCCTGAAGGTAGCCAAGGCCTCAATCAATGACCCCTTCACAACTATCAATGGTACTAGTACAGGTATCATCACTGTAGGGAATGTGATTGAGGTGGAAAACCTGATGGTCTCTAGTGGATTTGGGTCCTGCTTTGAAGGGAAGTTACTTCAACTCCAAACATTCTTTGAATCAATGGAGTGGCACTTCCCTGCTGACTTTTTAGAAGCAAGGATGACTATGGAATGTCTCTTCATGTCATCCCAAGTTTCCCACCTTTCTGGCTCTCAAAAATGGGGAGAAACTACCAAGCAAAGTAGGGCACTCGAGGCAATGGGATTGAGAGCTTGGAGGATGAGTAGAGACAATGTTCATGAGGCCAAGCAGCTGGTCAGGGAAGGAGAAAGTCTATATTGGATCAGAACAGATGGAGAGATTGGTAACCAAATGGTTTTGGGATATATGGGGACTCCACTAGTAAGAGTTTCAAGTTGGAGATGA

>LcGRAS8

ATGCCCCTAGCCTTTGAAGATTTTCAAGGGAAGGGGGCGTTAGATTTCTCTTCTTCTTCATATTCTTCTTCAGATTCACTTCTACAACCTCCACTACATCAAAAACAACAACAAGAAGAAGAAAACTGGCTCCTCAACAACAACAACAAGAAACAAGTTTGCTGCTATGTGGGCAGCACTACTACTACTGAGCCCACATCTGTTCTTGACACCACCAGTACCAGAAGCCCAAGCTCACCAACCTCTTCCTCAACACTGTCTTACTCTCTCGGAGGCAACACCAACGGCGGTGGTGGAGGTGGAGGAGGCGGAGGAGGAGGAGCCTCCACCGACACCACCGGCACTGCTTCAGCTGCAGCTACTGCCGCTAATGTCAACACCACCAACAACCCTTCACTAGACCTCGGCTCCACAGAAAAATGTGGGTTGGTAATGGAAGACTGGGAGGAGTTGTTGTCAGGGTCGCCTAGTCAAGAGCCATCCATTTTGAGGCTAATTATGGGTGATATTGATGACACATCTATGGGACTAAACAAGATTTTGCAACCTGGAACCCACCAAGACATTGACTTCAATGCAGGTTTTGGTGTTGTCGATCAAGCTAGTTTCGGTTTCGAGTCCTTGACTGCTCATCATAATAATATTGACCCTTCTTCTTTACATGTTACTGATTTCCCTGTTCATGCCGGCAATGCAAGGCTTGTTGGTTCGGTTTCAAACGCACCCCACATGTTCTCTTCTTCGTCAGCACCTAATCTTCTCTCTGTTTCGCTTCCACCTGGAGTTTTTCCACAGCAACCGATTGACGCCTTGGAAGAGAAACCACAGATATTCAATCCGCAGGTGATTATTAATCAAAGCCAAGCTCAGTTTGGGCAGAATCCAGCTTTGTTCTTGCCGGTTTCGTGTGGTGGTGGTCAATTGCAAGAGCATAATCTTTTGTCTCCTCCGCCACCAAAGCGGCTCAATTTTGGACCCAACAATGTTCAGGTCCCGAAAGTTGCGTTTTCAGATTCCGGTCAACAACAAGAGATGTTTCTTCGAAGACAACACCAACAACAGCAGCTTCAGTTGCTCCAACAACAACAACAACAACAAAGGCAAGCAATGGGAGTGACAGCAGCAGCAACGACAACAACGAAGCAGAAGATGGTAAATGAAGAATTGGCGGCGAACGAGCAGCTTCAGCAGGCTATAATCGACCAGATTTTCAAGGTTGCAGAGCTGATCGGGATGGGGAATCCGGCACTCGCGCAAGGGATATTGGCGCGGCTCAATCACCAGCTCTCACCTATTGGTAAGCCTTTTCAAAGGGCTGCTTTTTACTTCAAGGAGGCCTTGCAGTCACTTCTCCCCATCAACACCACTAATTCTGCTAGTGCAGCTTTCTCTAGTTTCAGTGTTATTTTCAAGATTGGTGCTTATAAGTCTTTCTCTGAAATATCGCCTATACTTCAATTTGCTAATTTCACTTGTAACCAAGCACTTCTTGAGGCATTTGAAGGGTGTGATAGAATTCACATTATAGATTTCGATATTGGTTATGGAGGGCAGTGGGCTTCTCTTATGCAAGAGCTTGTTTTGAGGAACGAGGGTCCTCCTTCGCTTAGGATCACAGCATTTGCTTCCCCGTCCACACATGACGAGCTCGAGCTTGGCTTTACACAGGAGAATCTTAAACACTTTGCTAGTGAAATTAATATGCCATTTGAGCTTGAAATTGTGAATCTTGAATCTTTGAACTCTGCTTCTTGGCCTATGCCTCCCCGCGAATTGGAAAATGAGGTGCTTGCTGTGAATCTCCCTGTTGGGGCGTTTTCGAATTACCCACCAACCCTTCCTTTGGTTCTTCGGTTTGTAAAGCAACTTTCGCCCAAGATCATTGTTTCATTGGATAGAGGATGTGATCGTGCTGATCTTTCGTTTCCTCACCACATGATTCAAGCCCTGTTATCTTACTCAGGTCTGCTTGAATCCCTCGATGCTGTGAATGTGAATTTGGATGCCCTGCAAAAGATTGAGAGGTTCCTGATTCATCCTTCCATTGAAAAGATTGTTTTGGGCCGCCATCGGACTCCTGAAAGATTGCCTCCTTGGAAGAGTCTATTTATGCAAGCCGGGTTCTCTCCATTGACATTTAGTAACTTCACCGAGTCTCAAGCTGATTGTCTGGTGCAGCGTACGCCGGTTAGGGGTTTACGCGCAGAAAAGAGACAGTCCTCGCTTGTCCTCTGCTGGCAGCGAAAAGACCTTATCTCGGCTTCAGCTTGGAGGTGCTAA

>LcGRAS9

ATGAAGGCCATGCCCCTAGCCTTTGAAGATTTTCAAGGGAAAGGGGTGTTGGTAAATTTCTCTTCTTATTTTTCATCTTCAGATTCACTTGCTCCACCACCACAACAGCAACAACAACAATATCATCAACTGCAAGAACAAGAAGATATTTTGCTCAACAAGAAGAAAAAGATTCAAGCTTGCTTCTATGTGGGCTCTAGTACTACTGAGCCCACATCTGTGCTTGAGACCAGAAGAAGCCTCAGTCCATCCATGTCTTCCTCTACACTGTCTTCCTCTCTTGGCAACACCACCACCACCACCACCAACGGTGGCGGTGGCGGCTGCGGCTGCAGTGCAACCGCCACTCCGGCTGCAAATGTCACCAGTGGTAATGTAGACATCATGAGTGGAGAAAAATGTGGTGGGTTGGTGATGGAAGATTGGGGTGGTGTGTTGTCAGGGCCTTCTAGTCACCAAGAGCAGTCCATTTTGAGGCTGATTATGGGGGATATTGATGACCCAGTCATGGGGTTGAACAGGATTTTGCAAAGTGGAAATGGGTCTAATCAAGATCTGGACTTTAATGCTGGTTTTGGTTTGGTGGATCAAGCTGCTGGTTTCGGGTTCGACCACCTGATGAGCAACAATAGCATTGACCCATGTGTTCATGTTAGTGATTTATCTGTTAATGCAAATGCAAGGCTTGGTCCTACCAATCTGATGATGCCTCCAGGAGTAGTGTTTCCACAGCAAGCAATGGAAGGTTTGGATGAGAAGCCCCAGATATTTAACCCACAGCTGATAATCAACCCCAACCAAGCTCAATTTGCCCAGAGTCCAGCATTGTGCTTGCCATTGCAAGAGCATAATAGTCTTTTGTCTCAACCACCAACAAAGCGCCTCAATTTTGGACCAAACCATGTTCAGGTCCCAAAAGTCCAGTTCCCGGATTCGAGTCAGCACGAGCTATTTCTTCGAAGACAACAGCAACAGCTTCAAATGCTTCAACAACACCAAGGGCAAGCAATGGGATTGTCACCACCACCACCCACAATAACAACAACAACGAAGCAGAAGATGGTAAATGAAGAATTGGCGGCGAACGAGCAGCTTCAGCAGGCTATAATCGACCAGATTTTCAAGGCTGCAGAGCTGATCGGGATGGGGAATCCGGCACTCGCGCAAGGGATATTGGCGCGGCTCAATCACCAGCTCTCACCTATTGGTAAGCCTTTTCAAAGGGCTGCTTTTTACTTGAAAGAGGCCTTGCAGTTACTTGCCCACATGAATGCCTCTAATTCTACTGCACCTTTGTCTAGTTATAGTGTTATTTTCAAGGTCGGTGCTTATAAATCTTTCTGTGAAATATCGCCTATACTTCAATTTGCTAATTTCACTTGCAACCAAGCACTTCTTGAGGCATTTGAAGGGTGTGAGAGAATCCACATTATAGATTTCGATATCGGTTATGGTGGGCAGTGGGCTTCTCTTATGCAAGAGCTTGTTTTGAGGACTGAGGGTCCTCCTTCGCTTAAGATCACAGCGTTTGCTTCTCCGTCCACACGTGACGAGCTGGAGCTGACTTTTACTCAAGAAAATCTTAAACACTTTGCTAGTGAGATTAATATGCCATTGGAGCTTGAAATATTGAACCTTGAATCTTTAAATTCTGCTTCTTGGTCACTGCCTTTATGCAAATTGGAAAATGAAGTACTTGCAGTGAATCTCCCTGTTGGGTCCTTTTCAAATTGCCCACCAACCGTTCCTTTGGTTCTTCGGTTTGTAAAGCAACTTTCGCCCAAGATCATTGTTTCTTTGGATAGGGGTTGTGATCGAGCTGATGTTCCATTTGCACACCATATGATTCATGCCCTTCATTCTTACTCCGGCCTGCTTGACTCACTTGATGCTGTGAATGTGAATTCGGATGTGCTGCAAAAGATTGAGAAGTTCCTGATTCATCCTTCAATTGAAAAGATTGTGTTGGGTCGCCATCGCACTCCTGAAAGGTTGCCCGATTGGAGGAGTCTCTTTATGCAATCAGGGTTCTCTCCATTGACATTTAGTAACTTCACCGAATCCCAAGCTGAGTGTTTGTTGCAACGGACTCCAGTTAGGGGTTTTCATGTTGAGAAGAGACAGTCTTCACTTGTTCTCTGTTGGCAACGAAAAGACCTCATCTCAGCTTCAGCTTGGAGGTGCTGA

>LcGRAS10

ATGATGAAAGGAGGTTTTGAAGTGGTTAATGGGGCTCTTGACATGATCCAACCCCATCATGAACCAATATGGGACTACTCCTCTATTGGGTTTCCACCAACTCCTACCTCTAACAACTTGATCCCGGTTAAACCCATGATCGAGAATCGCGTCAACTTTGAGAGAAATGAGCTGTCTGAGTGGGTCGAAAACATCACCAAGCAGCTCATCGATGACTTGCCTGACACTACTGGCTCTGATATCAGCCTCCAACCCGACCAAACAATGGTGTGCGAGGACAATATCAACCCAGTTGCATCTTTTTTGGGTGAGTTAAGGCCAAGAAAAACCATGAGAAGAAACAACTATTTTGATGGTGATCATCATGAATACCTTCAATGGAGCAGTAATATTGAGTTTGGAAACCAAACAAACGTTTGTCAAAACGCGACCCTCTTGAGCAATACTAGAGGGTTGAACAGATTAGACGAGCAAGGGCTAAGCCTGATAACCCTTCTCTTGGAATGCGCGGTGGCCATTTCTGTTGACAATCTCGGTGAAGCTCATCGAATGCTACTTGAGCTGACCCAAATGGCCTCACCCTATGGCCCTTCTTGTGCGGAGAGAGTGGTGGCCTATTTCGCCAAGGCCATGTCAAGTAGGGTTATCAACTCTTGGCTTGGGATTTGCTCTCCTTTGATCAACCACAAGAGCGTCCACTCCGCTTTCCAAGCATTCAACAATGTCTCCCCCTTCATCAAGTTCGCTCACTTCACTTCCAACCAAGCCATTCTAGAGGCGTTCCATCGGTGCAATAGGGTGCACATAATAGACCTCGACATCATGCAAGGCCTTCAATGGCCTGCTCTCTTCCACATCCTCGCTACACGTCTCGAAGGCCCTCCCCACCTTCGAATGACAGGGATGGGAACTTCGATGGACATTTTACTCGAGACGGGGAAACAACTCTCTAACTTTGCCAAACGCCTTGGACTCTCCTTCGAGTTCCACCCCATAGCCAAGAAATTCGGCGAAGTTGATGCCTCCATGCTACAACTCTGCCGCGGAGAGACACTCGCCGTGCATTGGCTACAACACTCTATGTATGATGCCACGGGAGCTGACTGGAAAACAATGAGGCTACTAGAAGAAATATCACCAAGAATCATCACATTGGTGGAGCAAGAAATTTGTCATGGAGGGTCTTTCTTGGACCGGTTTGTGGGGGCTTTACATTACTACTCTACCACATTTGACTCCCTAGGAGCATATTTGCCTAACGATGATGCGGGCAGGCACCGGGTCGAGCATTGCCTTCTTTACAGGGAAATCAACAACATATTGGCCGTAGGAGGGCCAGCAAGAAGTGGAGAGGACAAATTTAGACAGTGGAGACACGAGTTTGCCGCCAGGAATGGTTTTGCTCAGGTGGGGATGAGCAGCAACTCCATGGCGCAAGCGCAACTTATACTAAACATGTTCCCTCCTGCACATGGCTATAGCCTTGTACAAGGAGATGGGACACTCATGCTTGGATGGAAAGATACTAGCTTGTACACTGCTTCTGCTTGGACCTCTCATGCATCTGGATAG

>LcGRAS11

ATGGAGGAGGGTCGCCAGAAACTATCAACAGAAGAAATCATGAGAGTGGCTGGAGCAAGATACATACATTTCTCCGACCAGGGAATTCATGACCTTTCCATAGTAATGCACCCTTTTGGTTATGCTCTCTCGGGCTTATCCGAAGAAGAGACCAGAGATGTGGAACTAGTCCACCTTCTTCTAGCTGCAGCAGAAAAGGTAGGCTACCAACAATATGAACGAGCAAGCAGATTGCTCTCATGTTGCGAACGGATGGCCGCCGAGAGAGCTAATTCTGCGCAAAGGGTCGTTTACTATTTCGCTGAAGCTCTACGAAGGAGGATCGATAAAGAAACAGGAAGGATCACAGCTGAAGCCTTGGCAGATGAAAAGAGTCTAACTTATCATGGACTGGGTTTTAATATCTCATTTCTCAAATGCTATCAAAAGATTCCATTCAATCAGGTAATGTATTACGCAGCCATACAGTCAATATTGGAAAATGTTGCAACTGCAAGTGAGATCCATGTCATTGATTTTGAAATAAGGAGTGGAGTTCATTGGACAACCTTAATGCAGGCTCTTGCAGAGCGTGTCGAGCACCCCATTCGTCTCCTTAAAATAACTGCCGTTGGGCTTAAAGTTCAAGAGAAGGATATAGAGGAGGTAGGTAACATGTTGTCAAGCTTTGCTGAGTCTTTGAGCCTTCCTTTTTCGTTTAGTGCTGTATGTGTCTCATGTTTTTCAGATATTAAGAAAGAAATTTTAGAGATTCAAAAGGACGAATCGTTGATCATTTATTGTTCAATGATATTGAGAATAATGCTTGCGTGGCCTGGTTGCCTGGAAAACCTACTGGGGATGATAAAAAAACTCAATCCATCAATCATGGTCGTGGTTGAAATCGAAGCAAATCATAATTCACCCTCGTTTGTGACTCGTTTCACTGAGGCCTTGTTCTTCTACAGTGCATTTTTCGATGCCTTAGAAACTTGTCTGGATCAGGATATTGGAACAAGAACAATTATAGAGGAAACTTTGAACAAAGGGATCCGCAGCATTGTCGCGTTGGAGGGTGCTGATAGAACAGCTCGAAGCGTAAAGATTGATGTATGGAGAGCATTTTTTACAAGGTTTAGGATGGTGGAAATTGGATTCAGTGACTCTTGTTTGTACCAAGCTAATCTGCTTCTTACAAAGTTTCCTTGTGCCAGTTGTTGCAATTTTAAGAGGAATGGGAACAGTCTAATCATTGGCTGGAAGGGAACACCAATTCATTCGCTTTCGGTGTGGAAGTTCCCTCGAGAGAGAGGGAGATTGTTTCTAAACTCTAAATAA

>LcGRAS12

ATGTCAAGTGATTTGTTCTCTTTCACGCCGTTTGATTTCAATGAAATCCAAGGCGCTATTCTTGGAGATGTTAATAAAGAGCAAGAAGCGGCGCTGAAAGCAAGGCAACTGCAACATCAAATTTTTTCCGCTGAAGAGTTGGAACTGGGAGAAATTTCTTCTTCTGAACTTGGCAGCGCCTATCGAGAGAACAATGATGGCAATAAAGAATTCAATTTCTTCAACCATCAAAATCATATGATGCAGCAACAGCAACAACAGAAAAATCCTAGTGATATGATCTTAGATGGATTTCAACCTACTTTTGTTTTCCCTCCAGCGCAGTCTTTTCATGAGAGTCCAAAGCTGAAAAACATCCAAAGTAGCAGTGAAGAACTTGTCAAACCAGCCAAGGAAACGCCATCTCCGATTTCGTTATCTTCTCTAGAACTCCTCAGTAACTATGCAAGCGGATTCAAGAAATTGAAGAGAAAACAGTCCAACCACCCAACAAATGATGAAACGAATGAGGGGTGTCGCCAGAACCTATCGACAGAAGAAATCATGAGGGTGGCTGGTGCAAGGTACCTACATTTCTCTGCCCAGAGTTTCGATGATTTTTCAATGGTCATGCACCCTTTTGGTTATGCTCTCTCGGGCCTGTCCGAAGAAGAAACCAGGGACGTTGAATTAGTCCACCTTCTTCTAGCCGCAGCAGAGAAGGTAGGGTACCAACAATATGAGCGTGCAAGCAGACTGCTCTCACGTTGTGATTGGACTGCAGCAGAGAGAGCTAATTCTTTGCAAAGGGTCGTTTACTATTTCGCTGAAGCTCTTCGAGGGAGGATTGATAGAGAAACAGGAAAGATCGCAGCCCAGGAATTCGCATCTGGAACTGCTTTAACTGATCATGGGTTAAGTTACAGTGTTACATCCCTTAAATGCTACCAAAAGTTGCCATTTAATCAGGTACTCTATTACGCAGCAATCCAAACCATAAACGAAAATGTTCGAAATGCAAGTAAGATCCACGTGATTGATTTTGAAATCAGGAGTGGAGTTCAGTGGACAACCTTCATGCTGGTTCTTGCAGAGCATGAACAGCCTGTTGAGCTTCTTAAGATAACTGCTGTTGGGTTACAAATTCAAGAAAACGTTTTAGAGGAGGTAGGCAAGAAATTGTCAAGCTTCGCTGAGTCTTTGAACATTCCTTTTTCATTCAACATTGTTTGTGTCGCATGTTTCTTGGACATTAAGCACGAACTTTTCCAGACTCGAAACGATGAGTCATTAGTTGTTTATTGTGCAATGATACTGAGGACGATGCTCTCGCGGTCTAAATGCTTGGAGAATCTATTGTCGGTGATAAAAAAACTCAATCCATTGATCATGGTTGTTTGTGAAATTGAAGCAAATCACAATTCGCCCTCGTTCGTGAATCGTTTCACCGAGGCCTTGTTCTTCTATGGTGCTTATTTTGATAGCTTGGAAACTTGCTTGGACCAGGATATTGAGACAAGAACAGCCACGGAAGAAGTTTTAAACAAAGGGATTCGAAACGTTGTAGCGATGGAGGGTGCTGATAGAATTACCCGAAATGTAAAGATTGATGTGTGGAGAGCATTTTTCACAAGGTTTAGAATGGTGGAAATGGGATTCACTGGCTGCTGTTTGTACCAGGCTAATCAGTTACTTAATAGGTTTCCTTGCGCAAGTTGTTGCAAACTTGACATGAATGGGAAAAGCCTGATTATTGGGTGGAAGGGAACTCCAATTCTTTCTCTTTCTGTGTGGAAGTTCCCTATAGAGAGAAGGAGGTTCCTAAATTATAGGTTTGAAAATTAG

>LcGRAS1

ATGGCCAACGGGGTGTTCTCCTTGGAGGGGTTTAATTTTGGTGAGGTTCCTGATAAGCTTAGTTCTACTGATAACTATGGTTGTTCTAGTGTTGGGGGAGGGAAAAAAGGGAGGCAGTTTGGTTCTTATGCAGGAGAAGAGTGGGGCAAGTCTGGAGCTAATGGTTCAATGTATTGTGATTATGGTAGTTTTTATCAAGATGTGTCATCGGAAGAGGATATCGTGTGTTCTAAGTACAATCAACAGCAGACATATTCGGATTATGGTCTTTTGGACAATTTAAGGTTTGATAAGGCGTCTCCACCAGTACAAACATGTTTGGAGGAGATTTCAAAGCTTAGTGAAGTCCAAAGTGGGACTGACCAAGATGTGGTGGTAGATAACACCAAGAAAGAAAAGCAGGTTCCTTTCTCTTTAGCATCCTTGGAGCTGTTGAAGAGCTATGGAAACGGATTCAAAAGATTGAATGGAGAAAGAAGGATTGAGTCTAGTAATGATATAATGTTGACAAAGGACCCCAATCGAAAGCTGTCCACCGAAGAGATCATGAGGATTGCTGGAGAGAGGTTTATACGATCCTCCTTGCGAACGGTTGAGGATGTCGTGTCCATGCTAGGCAACCCTTTTGATCTCTCTTTCTCTGGCCTATCTGATGAGGAGATCAGAGATATTGAGCTTGCGGAGATTCTCTTGGCATCTGCTGAGAAAGTAGGTGATCAGCAGTATGAGCGCGCCACTAGGCTGCTCAACCAGTGTGATTACTTGTCTTCCCCGAAAGGTAATCCTGTTCAAAGGGTGGTTTACTATTTCTCTGAAGCTCTTCGGGACAGAATTAATCGAGAAACCGGCAGGGTCACATCGAAGGAGATGGGAAAAAGGGAGTCATTTGATATGGATGTGGCAATGATGAAGCCGAACCCTACTAGCCTGGCGTGTCATGCAGAACTTCCCTTTGGAATGGTTGGACAATTGGCTGGGATCCAAGCAATTGTAGAAAATGTGGCTGAGGCAAAGAAGATTCACATAGTTGATCTTGCTATCAGGAGTGGGGTGCAATGGACAGCATTGATACAAGCTCTTGCTTCTAGAATCGAATGTCCCGTTGAGCTTCTCAAGATTACTGCTATTGGAGACGCTGCAAGACATTTGATTGAGAATACAGGTAAGTGGTTAACGAACTTTGCGCAGAGCATGAACCTACCCTTTTCGTTTAATATCGTTATGGTAGCAGACATGTTAGATGTTAAAGAAGATATGCTTGAGATTGATGCTGAGGAAAGATTGGCTATCTTTGCTCACTACTATTTAAGAACCTTGATTGGGCTCCCTGATCGTCTGGAGAACATAATGAGAGTGATCAGGAACAGCAGTCCATGTGTAATGGTGGTCGCTGAAGTTGAGGCCAATCACAATTCACCGATATTTGTGAACCGTTTCATTGAAGCTCTTTTCTTCTTCAGTGCATATTTCGATTGCATAGATTCATGCATGAAACACGATGATACTCAGAGGATGATATTAGAATCAGTGTATTTTGCTGAGGGGATTAGAAGCATTGTGGCAAATGAGGGAGAGGAAAGGAAGATTCGAAGTGTGAAGCTTGATGTTTGGAGGGCATTCCTTGTGCGTTATGGAATGGAGGAGGCAGAACTGAGCATGTCATCCTTATATCAAGCAGACCTTGTGTTGCAGAAACTAGAAAGTTGGAAATCCTGTACAATAAATTTGGATGGAAAAAGCCTAATGTGTAGGTGGAAGGGTACGCCGATGCATTCGCTTTCAGTTTGGAAGTTCATATGA

>LcGRAS7

ATGGCAACAGCTGCAATTGTGAAAGCAAATTCACTGCTTTTGTCGAAGCGGAATGAAAGCCCTCATATCAACCTTTTTTACAATAACTCTGTCAAATTTAGCAGATGCTTTGATGGTAGGCTGAATACTTGGACTTTAGGTGCAACAAGCAAGAATAAGATTGTTGCTCCTTTAATAAAAATAGGGAACGAGATTGCTGCTCTCACTAGTTCAGAACAAAATCTAAAGGCAGAGATGTCGGATTCTCTCCATTCTAGGAGTGATTGTATCCTTACTAGGTGTGATTGTTTTTACTGTGCACATGCAAAGTCGAAAATTAAGACGGATGCTGTAGGAACGGCATTCGTAGCTACTTTGTTGACTGCTGTGGCTGTGCTATACATAGTGCATAAGATAGTGGAAGGGACAAAAGCATGCCTCTTCAAGATCAAGCGGCTTTTTTATCATCTCGTGATGAAATTAAAGCAGTTATGCGGGATTGTTCCACCTGTTGATGAAACTCTTAAGGAAACTATAGAGACTATTCCAGAACGCCATGTTGAAAATAACTCCAACGAAGAGGATAATTATCGTAAAGCAGAACTGGAGCAGTCCAATGATTTGATACATAAGCTTGGTCAGCAAATCAAAAATCATAACCAGCTCTCACCTATTGGTAAGCCTTTTCAAAGGGCTGCTTTTTACTTCAAGGAGGCCTTGCAGTCACTTCTCCCCATCAACACCACTAATTCTGCTAGTGCAGCTTTCTCTAGTTTCAGTGTTATTTTCAAGATTGGTGCTTATAAGTCTTTCTCTGAAATATCGCCTATACTTCAATTTGCTAATTTCACTTGTAACCAAGCACTTCTTGAGGCATTTGAAGGGTGTGATAGAATTCACATTATAGATTTCGATATTGGTTATGGAGGGCAGTGGGCTTCTCTTATGCAAGAGCTTGTTTTGAGGAACGAGGGTCCTCCTTCGCTTAGGATCACAGCATTTGCTTCCCCGTCCACACATGACGAGCTCGAGCTCTTTACACAGGAGAATCTTAAACACATGCCTCCCCGCGAATTGGAAAATGAGGTGCTTGCTGTGAATCTCCCTGTTGGGGCGTTTTCGAATTACCCACCAACCCTTCCTTTGGTTCTTCGGTTTGTAAAGCAACTCTCGCCCAAGATCATTGTTTCATTGGATAGAGGATGTGATCGTGCTGATCTTTCGTTTCCTCACCACATGATTCAAGCCCTGTTATCTTACTCAGGTCTGCTTGAATCGCTGGATGCTGTGAATGTGAATTTGGATGCCCTGCAAAAGATTGAGAGGTTCCTGATTCATCCTTCCATTGAAAAGATTGTTTTGGGTCGCCATCGGACTCCTGAAAGATTGCCTCCTTGGAAGAGTCTATTTATGCAAGCCGGGTTCTCTCCATTGACATTTAGTAACTTCACCGAGTCTCAAGCTGATTGTCTGGTGCAGCGTACGCCGGTTAGGGGTTTACGCGCAGAAAAGAGACAGTCCTCGCTTGTTCTCTGCTGGCAGCGAAAAGACCTTATCTCGGCTTCAGCTTGGAGGTGCTAA
